# Orchestrating Multi‐Ångstrom Spaced Cu─Ni Dual‐Atom Pair for Synergistic C─H Activation in Direct Methane Oxidation to Methanol

**DOI:** 10.1002/advs.202511661

**Published:** 2025-08-11

**Authors:** Jingting Jin, Wenzhi Li, Liqun Wang, Lulu Zhang, Xia Zhang

**Affiliations:** ^1^ Laboratory of Clean Low‐Carbon Energy University of Science and Technology of China Hefei 230023 P. R. China; ^2^ Institute of Energy Hefei Comprehensive National Science Center Hefei 230031 P. R. China; ^3^ School of Chemical and Blasting Engineering Anhui University of Science and Technology Huainan 232001 P.R. China

**Keywords:** C1 oxygenates, dual‐atom pair, methane oxidation, methanol, synergistic effect

## Abstract

Direct and efficient methane oxidation to methanol is an appealing route for upgrading abundant methane resources while acquiring building blocks of clean fuels and chemicals. However, owing to its highly symmetrical nature imparted chemical stability and steric hindrance, the design of multi‐ångstrom (<3.0 Å) spaced active species capable of activating its first C−H bond remains a fundamental challenge. Herein, Cu−Ni dual‐atom Pair is constructed using defect engineering and a stepwise deposition method over indium oxide to precisely modulate the C−H polarization with the Cu atom showing affinity to H end and Ni anchoring the C side. The optimal CuNi/InNT achieves an oxygenates (CH_3_OH and CH_3_OOH) productivity of 106 mmol g_cat_ h^−1^, surpassing reported systems. Theoretical calculations validate the dominating role of interatomic distance for methane activation. Specifically, the dual‐atom orbital coupling effect in the minimally spaced Cu−Ni pair up‐shifts the overall d‐band center, significantly enhancing its hybridization with C/O 2p. Further modification through macroscopic reactor design boosts CH_3_OH yield to 36818.84 µmol g_cat_ h^−1^ with 79.37% selectivity in a 1000 mL semi‐industrial prototype. This work provides a comprehensive explanation of the Cu−Ni synergy, bridging atomic‐scale catalysis with reactor design, and establishes a common design principle for binary catalysts at the electron and orbital level.

## Graphical abstract

This work constructs a sub‐ångstrom‐spaced Cu−Ni dual‐atom pair over defect‐rich indium oxide support for methane oxidation into methanol. With Cu/Ni showing affinity to C−H bond's H/C end, an outstanding primary oxygenates (CH_3_OH and CH_3_OOH) productivity of 106 mmol g_cat_ h^−1^ and a high oxygenates selectivity of 84.55% is achieved under mild conditions.

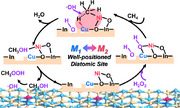

## 1 Introduction

With the continuous discovery of abundant methane (CH_4_), its functionalization could be a promising stepping stone towards a future clean energy system.^[^ ^1^, ^2^, ^3^ ^]^ Its oxidation into value‐added chemicals or liquid fuels, especially the direct and selective transformation of CH_4_ to CH_3_OH (MTM) is even viewed as the “Holy Grail” as it is an appreciable energy storage strategy that could simultaneously solve the energy crisis and mitigate the progressive climate deterioration.^[^ ^4^, ^5^, ^6^, ^7^, ^8^ ^]^ However, the activation of the first C─H bond (with an overwhelmingly high bond energy of 439 kJ mol^−1^) is intensely challenging, causing the existing route to be energy‐intensive with a huge carbon footprint,^[^ ^9^, ^10^, ^11^, ^12^ ^]^ which draws further away from the original intention of realizing carbon neutralization. Ergo, inspired by methane monooxygenases (MMOs)’ amazing ability for CH_3_OH production via natural biosynthesis,^[^ ^13^, ^14^, ^15^ ^]^ tremendous efforts have focused on the active species engineering at the atomic level to realize C−H bond polarization under mild conditions.

In the hope of that, dual or tri‐nuclear enzyme‐mimicking Fe/Cu sites have been constructed.^[^ ^16^, ^17^, ^18^ ^]^ Among those attempts, catalysts with diatomic active sites (homo‐nuclear or hetero‐nuclear, M_1_‐M_1_ or M_1_‐M_2_) have flourished considerably, appealing prospects for their possession of multiple adsorption/activation sites and dual‐atom synergy.^[^ ^19^, ^20^, ^21^, ^22^, ^23^ ^]^ However, the activation of CH_4_’s C−H bond is extremely sensitive to the spatial arrangement of active species. While overly large diatomic distances would hinder their synergistic effects, excessively small distances would lead to insufficient CH_4_ adsorption due to steric hindrance. Whereas this critical challenge of precise control of sub‐ångstrom (<3.0 Å) inter‐distance atomic spacing remains unrealized. Nevertheless, in homo‐nuclear species, the electronic structure of the M_1_‐M_1_ configuration would remain symmetrical to a certain extent, which may weaken their directional substrate polarization ability.^[^ ^21^, ^22^, ^23^ ^]^ Therefore, the rational design of hetero‐nuclear M_1_‐M_2_ dual‐atom sites with appropriate spacing that matches CH_4_’s C−H bond at the atomic precision is in keen demand,^[^ ^24^, ^25^, ^26^, ^27^, ^28^ ^]^ as such configurations enable the stretching of the two ends of the C−H bond and thus provide targetable weak points accessible to indiscriminate active oxygen species.

Posing to achieve such challenges, focusing on orchestrating a microenvironment with well‐positioned binary sites with scale‐up potentials by transition metals seems economically viable. Copper (Cu) possesses MMOs‐like activity,^[^ ^6^, ^29^, ^30^ ^]^ while nickel (Ni) exhibits excellent Carbon (C) affinity,^[^ ^31^, ^32^, ^33^ ^]^ making the Cu−Ni dual‐atom sites capable of synergistically polarizing the C−H bond in theory. Coupled with the factors of coordination situation, electronic state, ionic radius constitution, metal–oxygen bond (M‐O) length matching, indium‐based carrier with excellent compatibility with copper and nickel species was identified.^[^ ^34^, ^35^, ^36^, ^37^ ^]^ However, the synergistic effect between metals is critically governed by charge redistribution, effective atomic orbital overlap, and bonding strength. Consequently, atomic‐scale resolution of the local asymmetric configuration at Cu−Ni sites and elucidation of its structure‐performance relationship are imperative.

In this work, we constructed Cu−Ni dual‐atom pairs over indium oxide nanotube via defect engineering and photo‐deposition sequence modulation. The interatomic distance between Cu−Ni was calculated to be 2.438 Å, which was slightly longer than C−H bond. And with Cu/Ni's affinity to C/H atom, together, they facilitated the stretching of the C−H bond in joined hands, attaining the polarization effect of “1+1>2”. The optimal CuNi/InNT has been orchestrated with an attractive CH_3_‐R yield (106 mmol g_cat_ h^−1^, or 0.306 s^−1^ in the turnover frequency), a high oxygenates selectivity (84.55%), and a competent CH_4_ conversion rate (9.77‰), surpassing previously reported values. In addition, the structure‐performance relationship between the Cu‐M (M = Pd, Zn, Cu, and Ni) diatomic spacing and catalytic performance was systematically analyzed through DFT calculation, confirming that the interatomic spacing is an important indicator of C−H activation. Further operando analysis and calculations elucidated the mechanistic origin of superior performance in CuNi/InNT systems. And a semi‐industrial reactor was designed, remarkably boosting the CH_3_OH yield and selectivity to 36818.84 µmol g_cat_ h^−1^ and 79.37% at reduced energy input than laboratory benchmark.

## 2 Results and Discussion

### 2.1 Materials Design and Catalytic Activity

**Figure 1:**
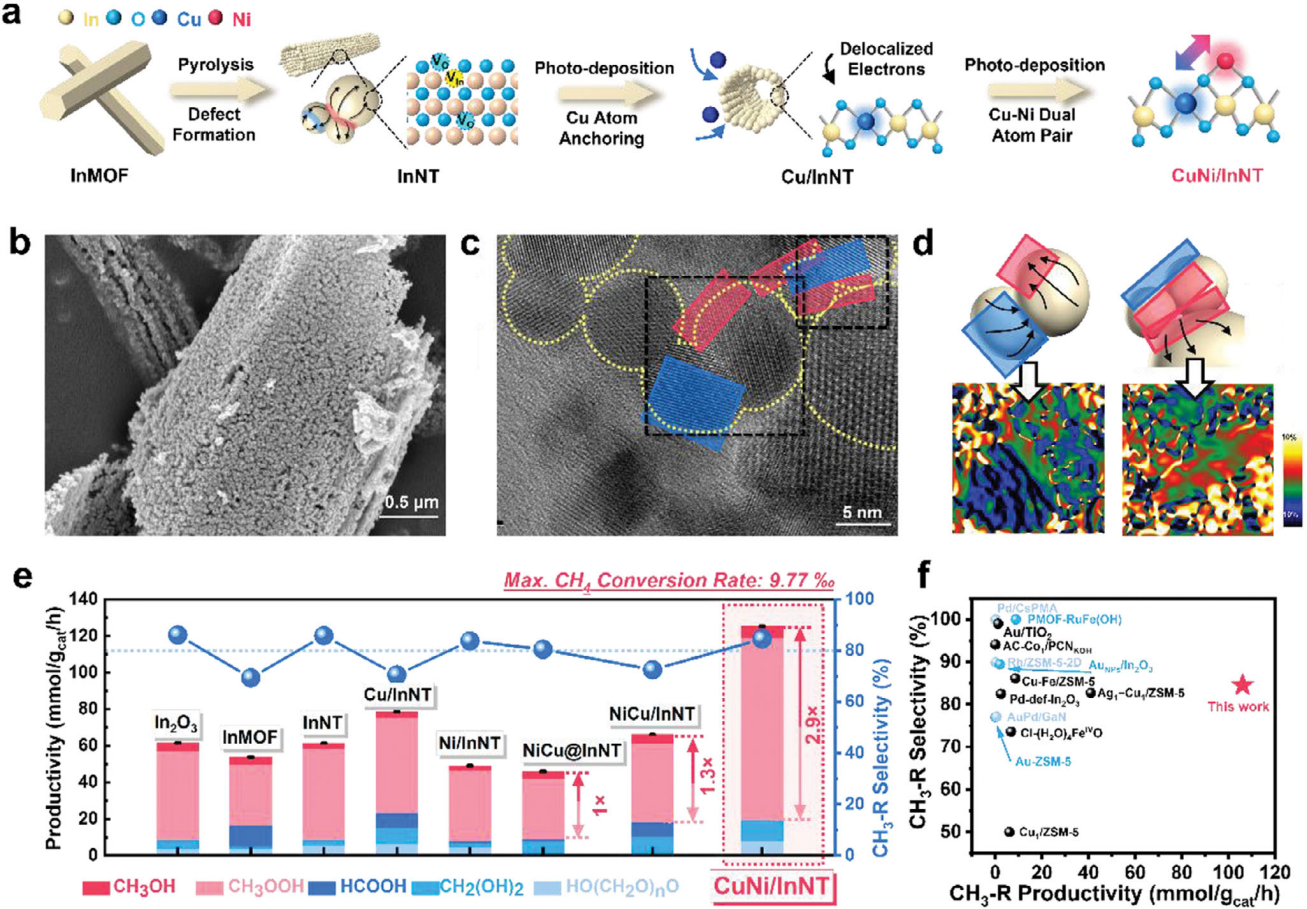
Defect engineering of InNT and CH_4_ oxidation experiments over CuNi/InNT. a) Schematic illustration of catalyst preparation. b) SEM and c, HRTEM images of InNT, the red box indicated the stretched strain area, while the blue box stood for compressed strain area. d) The GPA results corresponded to the figure 1c's black dots box areas. e) The CH_4_ oxidation performance of commercial In_2_O_3_ (Shanghai Aladdin Biochemical Technology Co., Ltd), InMOF, InNT, Cu/InNT, Ni/InNT, NiCu@InNT, NiCu/InNT, and the optimal CuNi/InNT. f) Comparisons with the representative catalysts on the yield and selectivity of CH_3_‐R.

The strategy of tuning the diatomic spacing follows steps of defect induction, metal anchoring, and diatomic generation which could be fulfilled by defect engineering and stepwise photo‐deposition. Firstly, following the principle of matching the length of metal oxygen bonds (M‐O) between various metals, identify carriers with excellent compatibility with copper and nickel species. Construct defects over the support to accommodate the first Cu or Ni species, and then the second metal is precisely positioned near the previous metal through photo deposition. based on this strategy, indium, recognized for its capability to facilitate hydrogen removal through interactions with active metals, has been extensively utilized as a support in heterogeneous catalysis, including carbon dioxide hydrogenation to methanol and other important reactions and its In‐O bond length is similar to Cu‐O and Ni‐O, providing potentials in semi‐doping anchoring of Cu or Ni species3. The Metal–Organic Framework (MOF) template method was employed to synthesize an InNT support with expanded surface area for active metal loading and enhanced mass transfer efficiency (**Figure** **1**a, Figures S1–S4, Note S1, Supporting Information).^[^ ^38^, ^39^, ^40^ ^]^ Interestingly, it was also characterized by significant tensile and compressive strains over the mainly exposed (222) facet. These strong strains were the macroscopic manifestation of numerous defective sites (both In and O defects) resulting from stacking errors or mismatches that occur during crystal growth, which was visualized by geometric phase analysis (GPA) (Figure 1b–d, Figure S5, Supporting Information), which in turn altered photo‐response behavior (Figure S6a, Supporting Information) and d‐band center.^[^ ^41^, ^42^, ^43^, ^44^ ^]^ The generation of additional In defects in InNT, evidenced by the pronounced envelope signal at g = 2.353 from in situ ESR tests, was particularly valuable (Figure S6b, Supporting Information).^[^ ^45^, ^46^ ^]^ They broadened the capacity for active metal loading by alleviating the steric hindrance associated with surface doping during the non‐synthetic process, allowing active metals like Cu to be not only on the surface but also embedded in the InNT carrier.

Once Cu was successfully embedded, the second photo‐excitation process would be affected by the differing electron delocalization status of Cu and In atoms, which could conveniently leverage the second metal with a strong C affinity, such as Ni, to accurately construct the diatomic sites. Given that, a series of MTM experiments was conducted on the aforementioned catalysts to verify the reliability of this core hypothesis for orchestrating Cu−Ni sites (Figure 1e). In addition to In_2_O_3_ and InNT pure supports, single metal modified Cu/InNT and Ni/InNT were investigated as precursors of binary catalysts, and most importantly, the CH_4_ oxidation behaviors of the Cu and Ni species simultaneously loaded NiCu@InNT sample, as well as the step‐wise loaded NiCu/InNT and CuNi/InNT were also examined in detail. The main products observed across all catalysts included CH_3_OH, CH_3_OOH, POM, HCOOH, and CH_2_(OH)_2_ (Figure S7, Table S1, Supporting Information entries 1‐8). Notably, the general CH_4_ conversion rate and C1 oxygenate (CH_3_OH and CH_3_OOH, abbreviated as CH_3_‐R) selectivity were improved over InNT compared to In_2_O_3_, which could be attributed to the unique morphology of InNT increased the solid‐liquid contact area, thereby in return enhanced the efficiency of heterogeneous catalysis.

Accordingly, the NiCu/InNT sample was prepared through a two‐step photo‐deposition method in which Ni was attached before Cu. The positive effect of Cu was greatly retained (Note S3, Supporting Information), in particular, this elevation effect was monotonically amplified to 3–7 times (Figure S8, Supporting Information), indicating that Cu and Ni have generated some kind of binary site. However, NiCu/InNT's reactivity was still lower than pure InNT, illustrating Ni's obstinate inhibitory effect for CH_4_ activation still exists. In order to suppress the negative effect of Ni and unwanted HCOOH generation while boosting the thriving influence of Cu on CH_3_‐R, it seems feasible to load Ni onto Cu/InNT, which might achieve Cu−Ni interaction while reducing the influence between Ni and InNT support, thereby reducing the negative impact of Ni. Experiments found that this CuNi/InNT showed an excellent CH_3_OH and CH_3_OOH productivity of 6500.68 and 99499.48 µmol g_cat_ h^−1^, respectively, with a total CH_3_‐R selectivity of 84.55% and overall CH_4_ conversion rate of 9.77‰, outperforming most catalysts reported under comparable conditions (Figure 1f, Table S2, Supporting Information). Its highest reactivity indicated that this modification route would allow Ni to grow near Cu to reduce Ni‐In electron transfer, ergo bypassing its diminished effect. On the other hand, no HCOOH was detected, suggesting the situation of Cu doping alone in InNT was also avoided. Therefore, the rational design of heteronuclear Cu−Ni binary active sites for the selective oxidation of CH_4_ was achieved with atomistic precision. Similar phenomena were observed in other materials prepared by this method of adjusting the loading sequence (Figure S9, Supporting Information). The influence of CuM/InNT (M = Pd, Zn, Cu, and Ni) interatomic spacing on C−H activation was discussed in detail in the following 3.3 section.

Based on the initial hypotheses of constructing a dual‐atom structure with adjacent positions, the simultaneous loading of Cu−Ni would lead to the formation of diatomic sites and foster synergistic interactions that improve catalytic performance. But when Cu and Ni were simultaneously introduced (NiCu@InNT), their reactivity was unexpectedly poor. This reduction in performance was attributed to the competitive adsorption between the two metals and the diminished capacity to generate free radicals from H_2_O_2_ (Note S2, Supporting Information). This outcome suggested that directly introducing two precursor salts limited viable diatomic site generation. Given the metal‐support interaction (MSI) between these metals and InNT, the step‐wise immobilization strategy to circumvent competitive adsorption and enrich the total number of competent active sites, once again proving the feasibility of the strategy, which sheds light on the design of noble‐metal‐free materials.

### 2.2 Structure Recognition of Dual‐Atom Species

**Figure 2:**
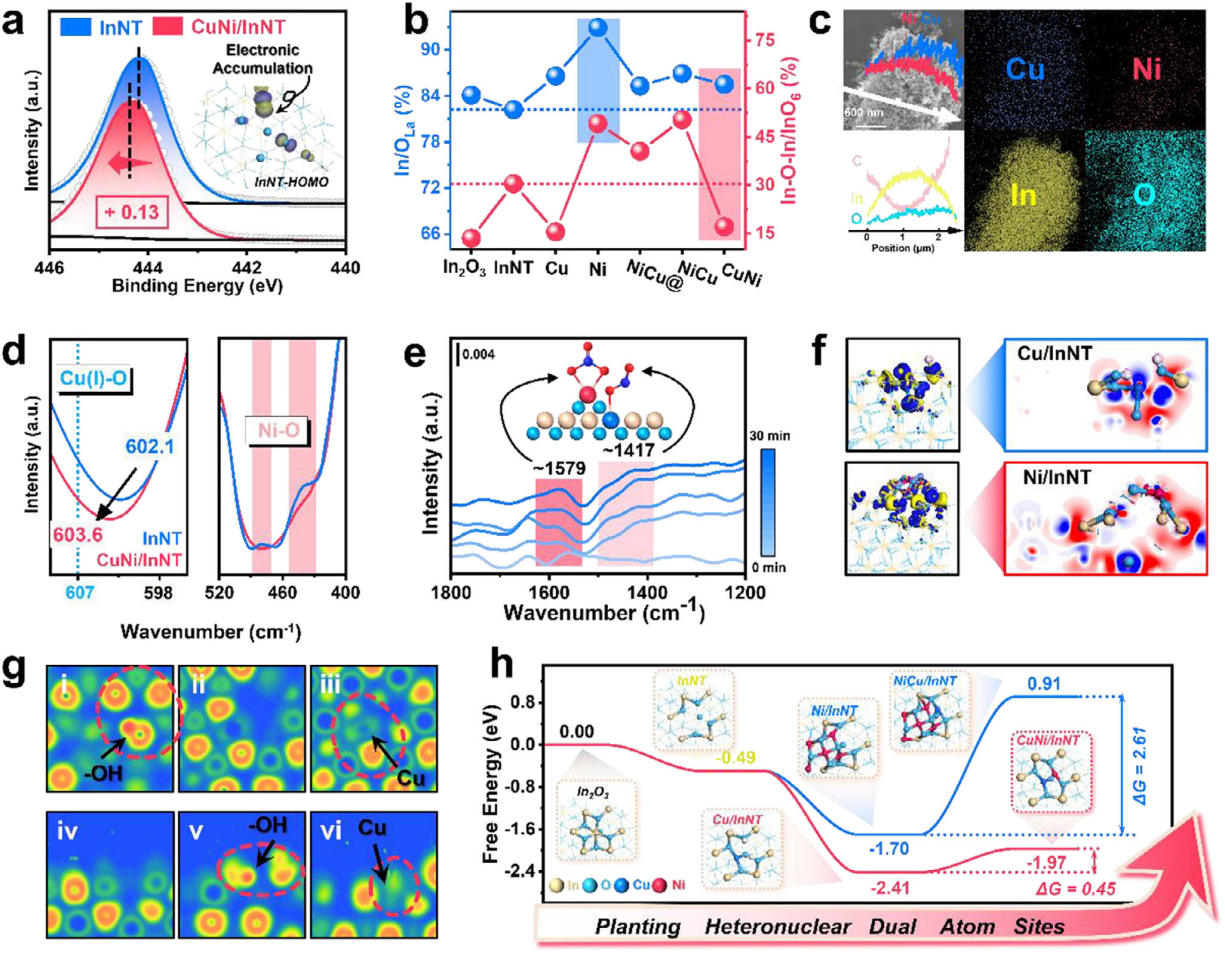
Structural characterization for active sites determination. a) In 3d XPS of InNT and CuNi/InNT, the insert image was calculated HOMO orbital of InNT. b) the atomic ratio of Indium and lattice determined by XPS, and the In‐O‐In/InO_6_ ratio quantified by Raman spectrum, the reference lines were the corresponding values of InNT. c) SEM, EDS, and line scanning results of CuNi/InNT. d) FT‐IR spectrum of InNT and CuNi/InNT. e) NO‐DRIFTS signals of CuNi/InNT. f) charge density difference diagram of Cu/InNT and Ni/InNT, taking Cu atom and Ni_5_O_4_ as sets, respectively. The isosurface value was set as 0.05, the blue section in the left 3D images was obtaining electrons while the yellow section was donating electrons. The blue section on the right 2D surface was obtaining electrons and the red section was donating electrons. g) calculated electron localization function results of Cu/InNT. h, the overall formation energy of all catalysts.

The structural characteristics of as‐prepared samples were thoroughly investigated, with extensive emphasis on the optimized CuNi/InNT to correlate the performance with structure. The XRD patterns were used to elucidate the crystal structure (Figure S10, Supporting Information). The variation in relative crystallinity was prominent in which InNT being the lowest (53.83%) among all samples, which originated from its crystal stacking error and disordered defects (Table S3, Supporting Information). Fitting results of XRD patterns revealed that Cu induces compressive strain, whereas Ni oppositely causes tensile strain (Figure S11, Supporting Information).^[^ ^47^ ^]^ Combining the optimal performance of CuNi/InNT, a conclusion of compressive strain (−0.115%) would induce the electrochemical transformation of metal species which affected the H_2_O_2_ and CH_4_ activation could be drawn. Compared to the In 3d 5/2 peak in InNT, a moderate shift (+0.13 eV) in CuNi/InNT indicated the electron donation effect of In to the Cu and Ni (**Figure** **2**a, Figures S12a, and S13, Supporting Information). In addition, the Highest Occupied Molecular Orbital (HOMO) of intrinsic InNT revealed a wide range of electron accumulation appearing on the defect sites, which was likely to trigger metal precursor adsorption during the photo‐deposition process. O 2p XPS demonstrated that Cu‐promoted surface −OH groups and Ni might induce the lattice oxygen out‐diffusion to form NiO clusters and O defects (Figure S12b, Table S4, Supporting Information). Notably, the ratio of In to lattice oxygen and the Raman deconvolution could reflect the In‐O coordination, providing information on defect situations (Figure 2b and Figures S14 and S15, Supporting Information). In decent agreement with the photo‐response EPR, more bulk V_O_ was illustrated on InNT compared to In_2_O_3_, which would gradually migrate to the surface during the photo‐deposition to form surface V_O_. Concurrently, abundant In defect (V_In_) and V_O_ sites existed in InNT, altering its photo‐response behavior and reserving adequate space for active metal cultivation. Cu species were likely to be shaped into tetrahedrally coordinated structures similar to Cu_2_O embedded in V_In_, with neighboring O atoms transforming into dangling ‐OH bonds. While Ni species in Ni/InNT and NiCu/InNT would agglomerate. Furthermore, EDS mapping found that both metals were uniformly loaded (Figure 2c and Figure S12c–e, Supporting Information). The normalized FT‐IR signal of CuNi/InNT displayed clues of Cu‐O and Ni‐O bonds, depicting that the connection mode of active metal on the InNT support was through a metal–oxygen (M‐O) bond (Figure 2d and Figure S16, Supporting Information). Thus, the microstructure of InNT with V_In_ and V_O_, the configurations of Cu's preferential embedment into In defects, and the preemptive agglomeration of NiO have been validated.

The adsorption configurations in NO‐DRIFTS are primarily associated with the surface defects and transition metals’ bonding modes to the support, thereby enabling the distinguishable identification of active sites (Figure S17, Supporting Information). Two unmistakable signals were observed over CuNi/InNT (Figure 2e, Table S5, Supporting Information). Integrating the aforementioned characterization analyses, the monodentate nitrite at 1579 cm^−1^ could originate from the surface‐isolated Ni atoms, while the bidentate chelating nitrate at 1417 cm^−1^ stood for embedded Cu single atoms, delineating a plausible Cu−Ni binary atom configuration over the premier CuNi/InNT sample. Consequently, a holistic overview of the crystal structure, surface morphology, chemical state, and adsorption characteristics of the aforementioned materials has been obtained. Leveraging the identified critical features, theoretical models encapsulating surface vacancies, MSI, and the electronic properties of active metals were constructed to deepen the understanding of the subsequent adsorption and activation of reactants. A 6‐coordinated In_2_O_3_ model was used as the foundation, and the (222) plane was designated as the active adsorption surface. The resulting unit model comprised 160 atoms with 96 oxygen and 64 indium atoms (Figure S18a, Supporting Information). Computations on multiple defect sites within a standard unit (In_1_O_6_) determined that the InNT configuration with neighboring V_In_ and V_O_ has the lowest formation energy (Figure S18b,c, Supporting Information).

In the context of Cu was embedded into cationic defect sites, and −OH was abundant on the Cu/InNT surface, a theoretical model in which Cu ions preferentially occupy V_In_, engaging in tetrahedral coordination with the surrounding oxygen atoms (Cu_1_O_4_) and featuring two dangling ‐OH bonds was cultivated (Figure S19, Supporting Information). In parallel, Ni/InNT was more conjectured to form an irregular, oxygen‐deficient, five‐atom cluster (Ni_5_O_4_) proximate to V_In_ (Figure S20a,b, Supporting Information). Prior experiments showed the opposite modification effect of Cu and Ni as single‐metal dopants, and the reactivity of CuNi/InNT and NiCu/InNT also exhibited divergent activities. This observation might be attributed to the disparities at the Fermi level between metal species and the support, leading to varying degrees of charge transfer at the metal‐support interfaces that manifest as unique interplays, thereby influencing the catalytic activity and the energy barrier for the subsequent deposition of a second metal species. Differential charge density analysis over monometallic Cu and Ni samples discriminated charge transfer from In to Cu and Ni (Figure 2f and Figure S21a,b, Supporting Information). Notably, Cu exhibited less pronounced charge transfer than Ni, which was consistent with XPS results. Upon quantification of the charge transfer via Mulliken population analysis, Cu was discerned to be +0.27 e. In contrast, the NiO cluster was +3.26 e collectively, equating to an average of 0.65 e per Ni atom (Table S6, Supporting Information, entries 4‐5). This indicated that an excessively robust MSI between the NiO and InNT impaired their ability to adsorb and activate CH_4_ and H_2_O_2_. To this extent, the configurations of Cu embedding in Cu/InNT and Ni forming NiO clusters on the surface in Ni/InNT have been confirmed by low‐temperature EPR and UV‐Vis‐EDR, once again verifying the validity of the theoretical models (Figure S21c,d, Supporting Information).

The Electron Localization Function (ELF) has been employed as a diagnostic metric for characterizing the distribution of delocalized electrons, and it also delineates electron transfer pathways and potential deposition sites for metal ions.^[^ ^48^ ^]^ It was ascertained that there was a pronounced degree of electron localization near −OH groups in Cu/InNT, which facilitated the subsequent adsorption of Ni^3+^ precursors, allowing Ni to be preferentially adsorbed in the vicinity of ‐OH within a triangular domain to construct a heteronuclear Cu−Ni binary active site (Figure 2g and Figure S22, Supporting Information). The aforementioned analysis of Ni/InNT discovered that Ni triggered the migration of oxygen atoms. This observation led to the consideration that there might be an oxygen atom bridging between Ni and Cu, forming an M‐O‐M structure. Subsequently, the possibility of Cu‐O‐Ni diatomic configuration in CuNi/InNT was explored. Computational results showed that the formation energy of the Cu‐O‐Ni configuration was elevated (2.02 eV), diminishing its likelihood. It is therefore posited that the Cu−Ni was directly connected as a highly asymmetric hetero‐nuclear structure, aligning with the initial anticipated (Figure S23a,b). For the alternative bimetallic loading sequence, ELF analysis identified stronger electron localization near the surface of the NiO c, Supporting Informationlusters in Ni/InNT, improving Cu adsorption. Stemming from steric hindrance effects, it is inferred that the proximity of In_2_ and In_7_ was more favorable for Cu adsorption (Figure S20c, Supporting Information), thus promoting the formation of single‐atom copper adjacent to NiO clusters in NiCu/InNT (Figure S23c, Supporting Information).

Up to this point, theoretical models for a variety of InNT‐based catalysts have been unraveled (Figure S24, Supporting Information). The formation energies for the overall support‐metal A‐metal B pathway have been scrutinized to be 0.45 eV for CuNi/InNT and 2.61 eV for NiCu/InNT (Figure 2h, Table S7, Supporting Information). This is likely due to NiO clusters obstructed Cu loading, impeding the dispersion and anchoring of Cu atoms in these areas, ergo repressing the production of effective bimetallic active sites. On the contrary, when Cu was loaded first, the emerged ‐OH would in turn offer preferential adsorption of Ni, promoting the migration and redispersion of Ni species, ergo effectively enabling the Cu−Ni dual‐atom generation.

### 2.3 Interaction Between Dual‐Atom Interatomic Spacing and Reactivity

**Figure 3:**
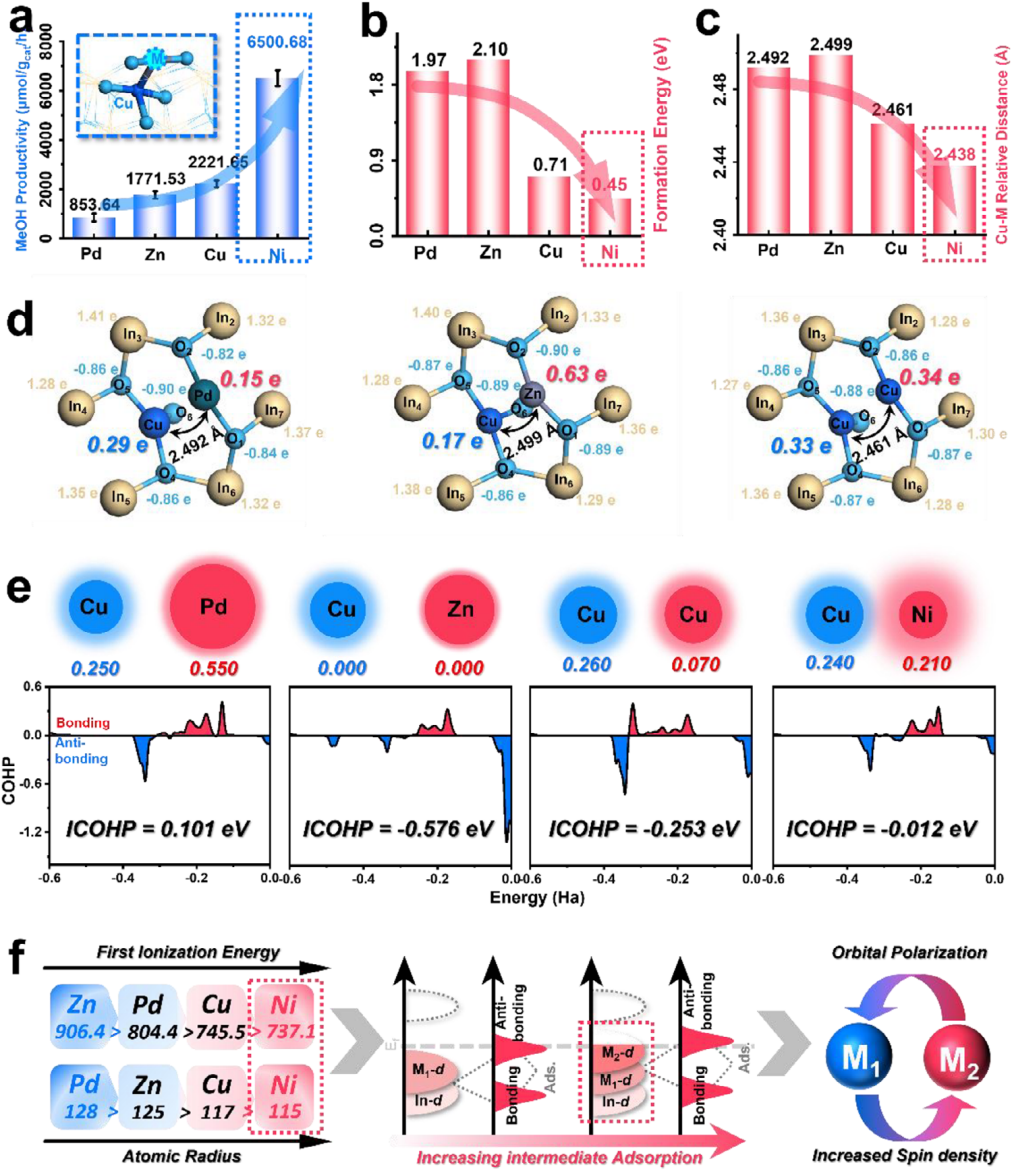
Constructing diatomic sites over InNT with Pd, Zn, and Cu. a) CH_3_OH productivity, b) formation energy, c) Cu‐M relative distance, and d) detailed Mulliken charges of CuPd/InNT, CuZn/InNT, and CuCu/InNT. e) dual‐atom interaction illustration with circle radius as ion radius, the size of the circular halo as the first ionization energy (the numbers under the schematic diagram were the spin density of the corresponding atoms), and the COHP results of above‐mentioned catalysts. f) illustration of factors affecting binary atomic sites’ catalytic reactivity.

As the process of preparing Cu‐M diatomic configurations through the stepwise photo‐deposition method was reliable, it is worthwhile to expand the exploration to other transition metals in order to reveal more general insights into the fine‐tuning of diatomic spatial distance and reactivity. Therefore, a series of Cu‐M (M = Pd, Zn, Cu) samples were prepared (named as CuPd/InNT, CuZn/InNT, and CuCu/InNT, respectively). Using the MTM reaction as a probe reaction, it was found that the CH_3_OH yield order followed a tendency of Pd<Zn<Cu<Ni (**Figure** **3**a). Drawing from previous analyses of NiCu/InNT, this may be due to the higher formation energies of these samples, preventing the formation of adequate active dual‐atom sites. Yet this was insufficient for the dramatic activity drop, for CuCu/InNT's formation energy was only 0.71 eV (Figure 3b), which only presented merely a third of CuNi/InNT's productivity (2221.65 µmol g_cat_ h^−1^). In addition, CO_2_+H_2_O_2_ experiments ruled out the possibility of activity differences caused solely by the H_2_O_2_ ‐ ·OH radical step, proving that the activation of C−H in CH_4_ by diatomic sites in the Cu‐M series samples was the key factor (Figure S25, Supporting Information). Therefore, in the conversion reaction of small molecule substrates, the number of effective active sites was certainly important, and the specific geometric configuration of their active sites played the leading role to effectively stretch and activate the target bond. And ergo, as an important geometric parameter, the interatomic spacing was analyzed as an excellent indicator for determining the activity of diatomic catalysts. DFT calculations revealed the variation in the Cu‐M spacing and found that the CH_3_OH yield was indeed positively correlated with the interatomic distance of heteronuclear sites (Figure 3c).

A universal analysis was carried out through electron distribution, d‐orbital coupling/polarization, spin density, and COHP (Crystal Orbital Hamilton Population) methods, to give a detailed interpretation upon the influence factors of different interatomic distances and the reasons for the ultimately catalytic behavior differences in MTM reactions. The d‐DOS diagrams revealed that the overall trend of the d‐band center (Zn > Pd > Cu > Ni) aligned well with the spacing trend as CuNi/InNT sample process with the shortest interatomic spacing, the highest d‐band center, and the most excellent reactivity. Particularly, there was almost no interaction between Zn‐In, implying that Zn‐d orbital hardly interacts with In‐d orbital, leading to poor stability, which was consistent with the formation energy of 2.10 eV (Figure S26, Supporting Information). Cu‐Zn was also poorly coupled (10.8%), indicating a potentially repulsive interaction between them, which hinders the formation of effective heteronuclear dual‐atom configurations. Additionally, despite Cu_1_ and Cu_2_ both having high coupling degrees with In at 65.2% and 88.5%, the homo‐nuclear Cu‐Cu structure would reduce the electronic structure asymmetry, making it difficult for their orbitals to be polarized, thus reducing the reactivity (Figure S27, Supporting Information). Therefore, selecting appropriate metals to shift the d‐band center upwards and modulate the orbital coupling and polarization between M_1_‐M_2_ is key to generating effective dual‐atom sites for CH_4_ and H_2_O_2_ activation. Nevertheless, the inferior reactivity of Pd was not entirely attributed to d‐orbital hybridization, prompting the investigation from the perspective of charge transfer. Pd has a relatively low electron density (Mulliken charge of 0.15 e), which was inadequate for CH_4_ and H_2_O_2_ activation. Hence, CuPd/InNT would fail to stabilize intermediates and facilitate the necessary bond formation (Figure 3d). While Pd did have a higher spin density of 0.55, providing a denser electron cloud around the Pd site. But it would enhance steric effects and hinder reactant molecules from accessing the active site, ultimately leading to the poorest catalytic performance (Figure 3e and Figure S28, Supporting Information). In contrast, Zn has a spin density of 0.00, and Cu_2_ in CuCu/InNT has a spin density of 0.07, which was not enough for ^*^OH and ^*^CH_3_ generation. Only the Cu−Ni dual‐atom site possessed the appropriate spin density to act as two anchor points for the efficient activation of CH_4_ and H_2_O_2_. In the view of bonding strength, the ICOHP (integrated COHP) of Cu‐Zn was ‐0.576, evidencing the strongest bonding tendency among all samples, which could originate from the intensified s‐p interaction (Zn‐4s with Cu‐4s and 3p) to provide a covalency and stable bonding, despite the limited d‐d coupling. However, this tendency would result in structural rigidity and inhibit the dynamic interaction between active sites and reactants. On the contrary, the Cu‐Pd bonding was extremely weak (ICOHP = 0.101), limiting the directional electron transport ability and structural stability. The Cu−Ni bimetallic catalyst achieves moderate d‐orbital coupling and M_1_‐M_2_ bonding strength with minimal atomic spacing, combined with moderate spin density and flexible charge distribution, making it an ideal design for efficient catalytic active sites.

Consequently, the regulatory mechanism of synergistic optimization of geometric structure and electronic situations in breaking through the catalytic activity bottleneck was highlighted, and the universal pivotal law of diatomic catalyst modification was obtained. Hetero‐nuclear sites surpass homo‐nuclear sites: hetero‐nuclear species would break electronic symmetry to induce directional charge transfer, and are more prone to form multifunctional active sites; Geometric structure optimization to reduce the diatomic spacing: screening secondary metals with larger atomic radius and lower first ionization energies to minimize the initial spacing between these atoms to stretch the targeted key bonds of substrate; Bonding strength adaptation to the reaction kinetics: d‐orbital coupling can elevate the d‐band center to adsorb the reaction substrate, while the strong covalent‐like bonding between M_1_‐M_2_ leads to structural rigidity, while weak interaction reduces electron transfer efficiency. Moderate M_1_‐M_2_ synergy balances the bonding strength and structural flexibility, achieving the optimal solution for intermediate adsorption‐desorption; Spin density matches the oxidation‐reduction demands: diatom centers need to maintain a certain level of unpaired electrons within the target reaction range. As if it is too high, excessive electron localization would hinder the reactants from contacting the active sites. While an inadequate situation will deprive the free radicals mediated activation pathways.

This series of discoveries establishes a cascade regulation framework that links intrinsic atomic properties (atom radius, first ionization energy) to modulated interatomic distance and charge transfer propensity, d‐orbital coupling/M_1_‐M_2_ bonding strength, orbital polarization degree, spin density distribution, and eventually to catalytic performance (Figure 3f), providing common design principles for expanding the application of dual‐atom catalysts engineering in C−H bond activation and other small‐molecule conversion processes.

### 2.4 Operando Analysis of C−H Activation Ability

**Figure 4:**
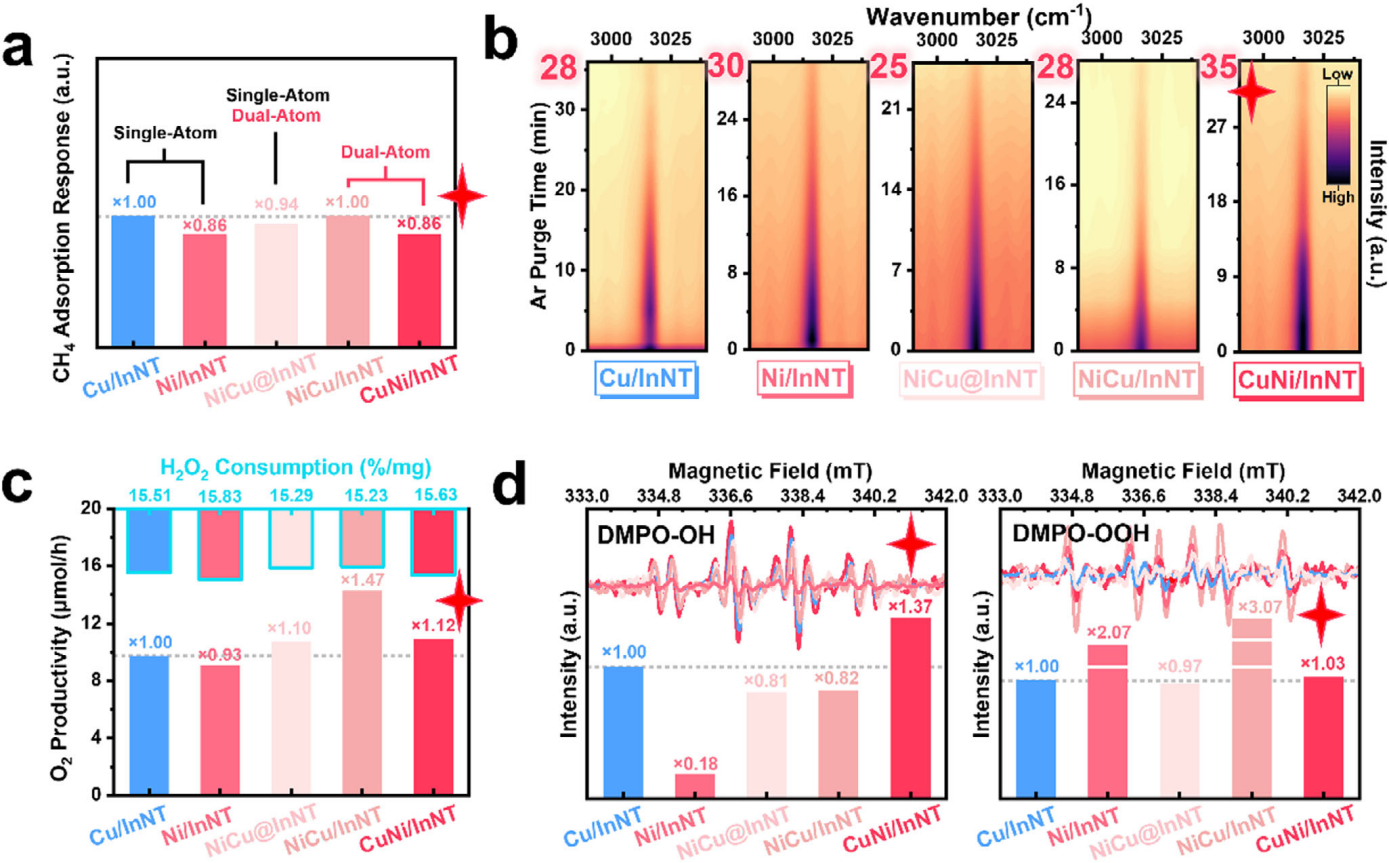
Reaction mechanism exploration through experimental and characterization methods. a) CH_4_ adsorption response (semi‐quantified by the fully adsorbed time of CH_4_‐DRIFTS), b) CH_4_ adsorption intensity using Ar purge time as the descriptor, c) O_2_ productivity and H_2_O_2_ consumption, and d) radical productivity of Cu/InNT, Ni/InNT, NiCu@InNT, NiCu/InNT, and CuNi/InNT. Note that in order to analyze the effect of active metals on reactant molecules more clearly, a semi‐quantitative analysis was conducted utilizing Cu/InNT’ properties as a standard (×1.0).

For the supreme CuNi/InNT, the identification of key influencing factors, reasons for by‐product formation, and the specific pathways for CH_3_OH and CH_3_OOH generation remained unclear, necessitating further in‐depth elucidation. DRIFTS analysis was conducted to clarify the CH_4_ adsorption behaviors. The lattice distortion and ample defect sites in InNT diminished its adsorption response time than In_2_O_3_ (Figure S29a, Supporting Information). Cu has a beneficial impact on this factor, promoting it to match the pristine In_2_O_3_ support, indicating that Cu may compensate for the In defects in InNT (**Figure** **4**a). Similarly, Ni also played a positive role in CH_4_ adsorption response, and its effect was more pronounced compared to Cu. Whereas for the three bimetallic catalysts, the adsorption response and metal loading were not simply a multiple relationship, once again pinpointing the formation of binary active sites over those samples. CH_4_ adsorption intensity directly influences their activation on the catalyst surface, which in turn affects the productivity and selectivity of CH_3_‐R as weak adsorption might render inadequate activation, and excessively strong adsorption could result in over‐oxidation. Ar purge experiments revealed that CuNi/InNT has the strongest adsorption intensity, proving its superior catalytic efficiency, which also benefited the propensity for subsequent oxidation (Figure 4b and S29b,c, Supporting Information). Only ^*^CH_3_ species was detected while commonly encountered intermediates such as ^*^CH_3_O, ^*^CH_2_, and ^*^CH_2_O in MTM reaction were absent (Figure S30, Table S8, Supporting Information), indicating a more singular surface pathway for CH_4_ on this catalyst.

H_2_O_2_ primarily participates in oxidation reactions through decomposition into ·OH and ·OOH radicals. But when decomposed into O_2_, it would indirectly engage in reactions with limited oxidizing ability. Therefore, the increased production of O_2_ on the NiCu/InNT catalyst corresponded to its lowered CH_3_‐R yield (Figure 4c, Table S1, Supporting Information). ·OH and ·OOH trapping experiments found that the optimal CuNi/InNT catalyst exhibited the highest intensity of ·OH (Figure 4d). In contrast, the minimal ·OH generation by Ni/InNT suggested overly strong MSI interaction would notably hinder the adsorption and activation of H_2_O_2_, consistent with the previous discussion. Recalling the CH_3_OH/CH_3_OOH ratio, a reduction was found in InNT compared to In_2_O_3_, indicating that the adjacent V_In_ and V_O_ favor ·OOH generation (Table S1, Supporting Information). Interestingly, CuNi/InNT only produced a moderate amount of ·OOH, which contradicted the instinctive assumption that CH_3_OOH formed through the combination of CH_4_ and ·OOH. In CuNi samples, the formation of CH_3_OH and CH_3_OOH did not follow a seesaw pattern like ·OH and ·OOH, implying that their generation may not be governed by parallel pathways. Quenching experiments were performed on InNT and CuNi/InNT to delve deeper into the reaction mechanism. The total CH_3_‐R yield of CuNi/InNT post ·OH quenching merely remained (12%), signifying ·OH as the dominant radical (Figure S31a, Supporting Information). Oddly, the CH_3_OOH ratio showed an increase when ·OH was removed (Figure S31b, Supporting Information). Hence, CH_3_OH and ·OH were deduced to be the intermediates for CH_3_OOH. Also, despite the quenching of ·OH there was still 20.3% of CH3OH remained, suggesting a potential conversion of CH_3_OOH to CH_3_OH.

In summary, the reaction pathways could include parallel pathways from ·OH to CH_3_OH and ·OOH to CH_3_OOH, as well as secondary radical reaction pathways from CH_3_OH and ·OH to CH_3_OOH, and the conversion of CH_3_OOH to CH_3_OH. This explains the significant increase in CH_3_‐R yields originated from the exceptional production of ·OH radicals in CuNi/InNT.

(1) $$*CH_{4}\to \;*CH_{3}+*H$$

(2)

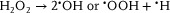



(3)

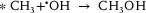



(4)

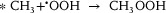



(5)

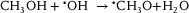



(6)

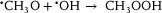



(7)

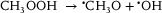



(8)

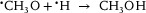




Notably, the optimal CuNi/InNT catalyst did not excel in all features. Therefore, correlation analysis was performed to explore the structure‐activity relationships and reaction pathways. It revealed that Cu−Ni bimetallic modification enhanced CH_4_ adsorption and ·OH generation. Moreover, CH_4_ adsorption and H_2_O_2_ decomposition may not only be independent processes. They were also likely to occur sequentially at the same active site, with H_2_O_2_ adsorption before the activation of CH_4_ (Figure S31c, Note S4, Supporting Information) as evidenced by the following DFT calculation results.

### 2.5 Theoretical Investigation of Cu−Ni Synergistic Effect and Overall Reaction Mechanism

**Figure 5:**
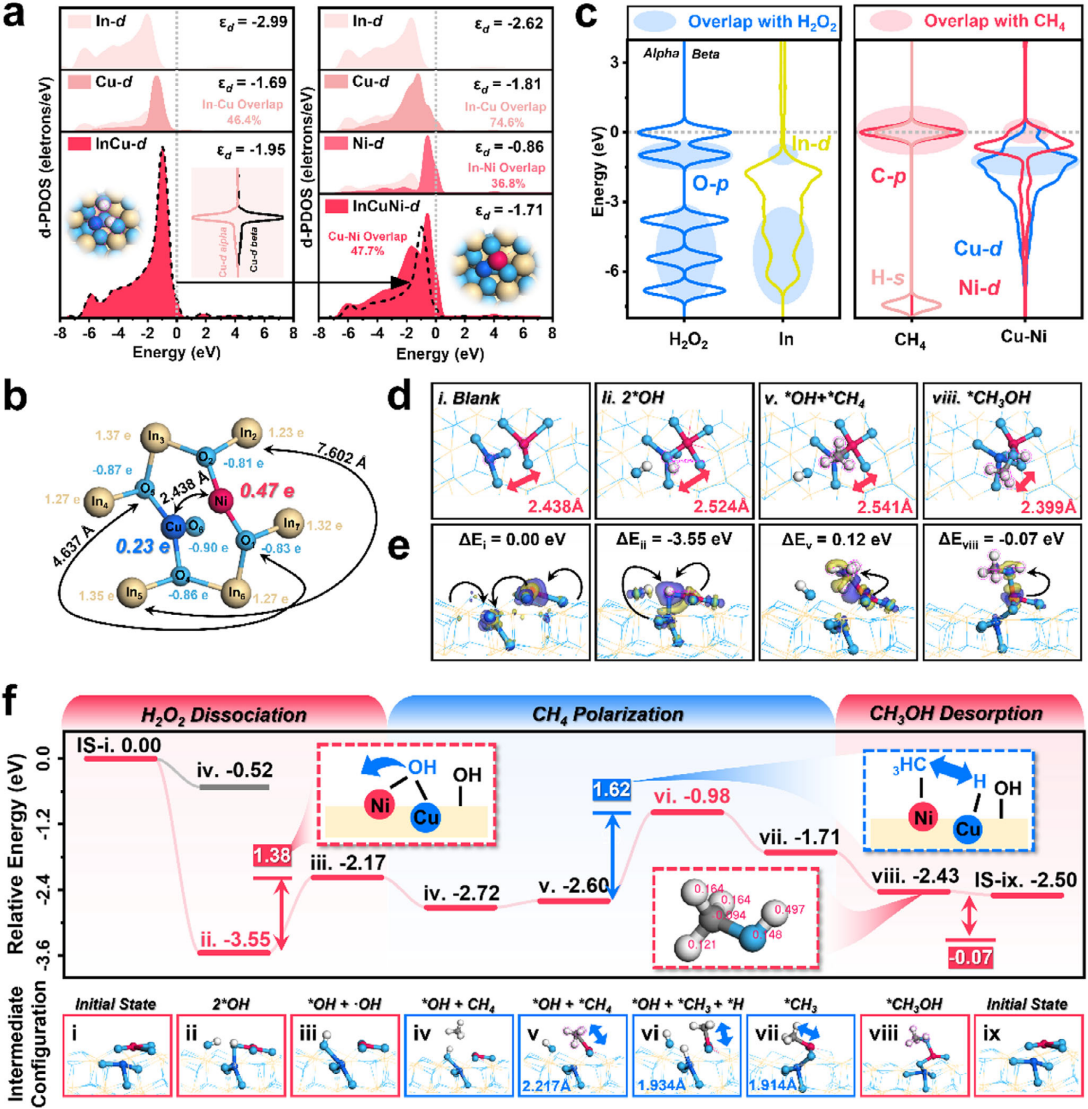
Reaction mechanism exploration through DFT calculation. a) projected density of states (PDOS) for the total d orbitals of Cu/InNT and CuNi/InNT. b) detailed Mulliken charges and relative distances of CuNi/InNT. c) calculated O‐2p orbitals in H_2_O_2_, C‐2p orbitals and H‐1s orbitals in CH_4_, and In‐3d, Cu‐3d, Ni‐3d orbitals in CuNi/InNT. d) Cu−Ni distance variations in 4 important intermediates. e) calculated charge density difference of configurations with step energy barriers noted above (blue for obtaining electrons, yellow for donating electrons, black arrows for possible charge transfer tendency). f) the overall energy barriers of H_2_O_2_ dissociation, CH_4_ polarization, and CH_3_OH desorption over CuNi/InNT.

The detailed reaction pathways have been ascertained, yet the origins of CuNi/InNT's high reactivity warrant further elucidation. Substrate binding strength was evaluated by the d‐band center and charge transfer of Cu and Ni (Figure S32, Supporting Information). In Cu/InNT, where Cu was embedded within InNT, its d‐band center shifted the overall d‐band center upwards, yet the minimal alpha/beta orbital asymmetry evidenced low Cu‐3d polarization (**Figure** **5**a).^[^ ^49^ ^]^ While in CuNi/InNT, Cu−Ni d‐d orbital overlap reached 47.7% (I_Cu‐d_/I _Ni‐d_, using the overlap of d‐orbital areas under the Fermi level as an identifier for identifying d‐d coupling). This hybridization of Cu‐3d, Ni‐3d, and the interaction between In‐Cu resulted in an even higher d‐band center, ergo optimized adsorption of intermediates. Additionally, compressive strain from V_In_ and Cu/Ni loading also contributed to the d‐band center's upper shift (Figure S33, Tables S9 and S10, Supporting Information). Ni disrupted Cu‐In symmetry, and polarized the Cu−Ni d‐orbitals, creating asymmetric overlap orbitals with H_2_O_2_ and CH_4_. The Cu−Ni distance in this unique heteronuclear diatomic site also eliminated adsorption steric hindrance for its distance (2.438 Å) was only slightly larger than CH_4_ (1.862 Å) (Figure 5b).

The partial overlap of O‐2p in H_2_O_2_ with In‐4d and Cu‐3d demonstrated CuNi/InNT's strong coupling affinity to H_2_O_2_, suggesting that H_2_O_2_ dissociation primarily occurred at these two metal sites, consistent with EPR trapping results. Likewise, CH_4_ would adsorb on the Ni site, specifically, it also effectively overlapped with Cu 3d‐beta orbital due to Cu's polarization (Figure 5c). Considering the experimental evidence of H_2_O_2_ dissociation first, followed by CH_4_ activation over the same site, a comprehensive reaction pathway including H_2_O_2_ dissociation (step ii), CH_4_ adsorption polarization (step v), and CH_3_OH desorption (step viii) was outlined (Figure 5d,e).

CuNi/InNT exhibited an exceptionally low barrier of −3.55 eV for H_2_O_2_ adsorption and dissociation (step ii). This ability came from the charge transfer at Cu−Ni sites, with In to Cu−Ni and Ni to Cu, and the presence of V_O_ also facilitated O‐O bond cleavage (Figure S34, Supporting Information). After O‐O breakage, the 3d orbitals of Cu and Ni were further polarized with a Cu‐OH‐Ni configuration (Figure S35a, Supporting Information). Concurrently, under the influence of this bridged O, the d‐band center of Cu shifts from −1.81 to −2.08, Ni from −0.86 to −1.31 (Figure S35b, Supporting Information), and Cu−Ni distance extends to 2.524 Å, resembling the unstable configuration of CuNi/InNT‐2. The differential charge density results using bridged ‐OH in Cu‐OH‐Ni as set revealed that this ‐OH species would obtain abundant electrons from the connecting Cu and Ni atoms (Figure S36a, Supporting Information). And this electron‐enriched surface ‐OH species with O's spin density to be 0.12, had a bigger possibility to desorb as ·OH radical (Figure S36b, Supporting Information). In addition, this other O 2p orbital presented a higher density near the Fermi level than O in Cu‐OH‐Ni, implying stronger bonding with surrounding In atoms, favoring its retention as surface *OH groups (Figure S36c, Supporting Information). Therefore, the primary reason for CuNi/InNT's high activity was that the Cu−Ni site significantly benefited H_2_O_2_ dissociation into ·OH+^*^OH.

Interestingly, the adsorption energy barrier was −3.55 eV for H_2_O_2_ and −0.52 eV for CH_4_, demonstrating a priority for H_2_O_2_ (Figure 5f and S37a, Supporting Information). Despite the 1.38 eV barrier at steps iii, there was an overall downward trend from the initial state to ·OH desorption. Besides, the remained non‐desorbed ^*^OH boosted CH_4_ affinity (step iv), making it more inclined to spontaneously adsorb CH_4_ after H_2_O_2_ dissociation (−0.54 eV). The second critical step was CH_4_ polarization (step v). In the ^*^OH+^*^CH_4_ configuration, Cu's Mulliken charge dropped to 0.410 e while Ni's rose to 0.83 e, and the ^*^CH_4_ motif obtained electrons to ‐0.330 e (Table S11, Supporting Information). The overlap point between the 1s orbital of H_4_ and C‐2p was the lowest, suggesting this C−H_4_ bond was the most vulnerable, making it more susceptible to dissociation (Figure S37b, Supporting Information). Ample space was prepared for Cu‐H adsorption as the Cu−Ni distance stretched to 2.541 Å. The energy barrier for CH_4_ to ^*^CH_3_ + ^*^H was 1.62 eV (step vi). The released ^*^H would combine with surface ^*^OH to form H_2_O and desorb spontaneously, leaving a stable ^*^CH_3_ intermediate (step vii). The shortening C‐Ni bond (2.217 Å → 1.934 Å → 1.914 Å) also validated this tendency. Abundant charge density difference and strong Cu−Ni 3d orbital overlap signified strong C‐Ni interaction and stable CH_3_ adsorption as well (Figures S38 and S39a, Supporting Information). Especially, the formed ^*^CH_3_ intermediate showed merely any spin density accumulation, suggesting it might be difficult to desorb as ·CH_3_ radicals. Consequently, the formation of ^*^CH_3_OH became a highly favorable process with an energy barrier of ‐0.72 eV.

The desorption of CH_3_OH marked the selectivity‐determining final step. In step viii, Ni's d‐band center moved from −1.07 to −1.15, signaling reduced Ni‐O interaction. Cu's d‐band center descended from −1.56 to −1.80, reflecting the weakening of excessive d‐d coupling between Cu−Ni that was enhanced by ^*^CH_3_ (from 67.6% to 57.9%) (Figure S39b, Supporting Information). Meanwhile, the Cu−Ni distance narrowed to 2.399 Å. These changes showed the Cu−Ni active site's tendency to revert to its initial configuration. ^*^CH_3_ could react with both ·OH and ·OOH radicals to produce CH_3_OH and CH_3_OOH, respectively. Yet the desorption energy of CH_3_OOH was 0.70 eV, whereas CH_3_OH desorbed spontaneously (−0.07 eV) (Figure S40a–c, Supporting Information). This is attributed to stronger charge transfer between ^*^CH_3_OOH and the catalyst (Figure S41, Supporting Information). The lower position of O 2p signal in the ^*^CH_3_OOH DOS diagram suggested that those electrons were more stable (Figure S42, Supporting Information). On top of that, the Ni‐O bond in CuNi/InNT‐^*^CH_3_OOH was shorter than the ^*^CH_3_OH configuration (2.005 < 2.127 Å), while its Cu−Ni bond length showed a significant extension (2.005 < 2.127 Å), evidencing the strong adsorption of ^*^CH_3_OOH to a point that even might uncouple the Cu−Ni interaction, ergo hindering its desorption (Figure S43, Supporting Information). With CH_3_OH identified as a precursor to CH_3_OOH from experimental findings, we proposed that the CuNi/InNT catalyst primarily followed the CH_3_OH pathway known as pathway (1). The H at the OH end in CH_3_OH, with a radical Fukui function value of 0.497, was most likely to be targeted by excess ·OH radicals, leading to the conversion of CH_3_OH to CH_3_O· and then CH_3_OOH via pathway (3) in the solution (Figure S40d,e, Supporting Information).

Thereby, all intermediate conformations and their conversion processes were comprehensively elucidated (Figure S44, Supporting Information). CuNi/InNT's core competence was its precisely tunable local coordination environment through d‐d orbital coupling favored H_2_O_2_ dissociation into ·OH. And the remaining adsorbed ^*^OH would improve CH_4_ affinity. With C bonded to Ni, and H_4_ connected to embedded Cu, the C−H bond was cleaved to ^*^CH_3_. ^*^CH_3_ Preferentially coupled with ·OH to generate ^*^CH_3_OH, which desorbed spontaneously and converted to CH_3_OOH with supernumerary ·OH (Figure S4, Supporting Information5). Nevertheless, the CH_4_ dissociation was markedly lower at −1.63 eV over InNT, but it only embodied limited CH_3_‐R yield with a higher proportion of CH_3_OOH (Figure S46, Supporting Information). This was attributed to V_In_‐V_O_ sites being more conducive to ·OOH radicals, making its product generation mainly follow pathway (2) (Figure S47, Note S5, Supporting Information).

### 2.6 Scale‐up Explorations of Methane to Methanol

**Figure 6:**
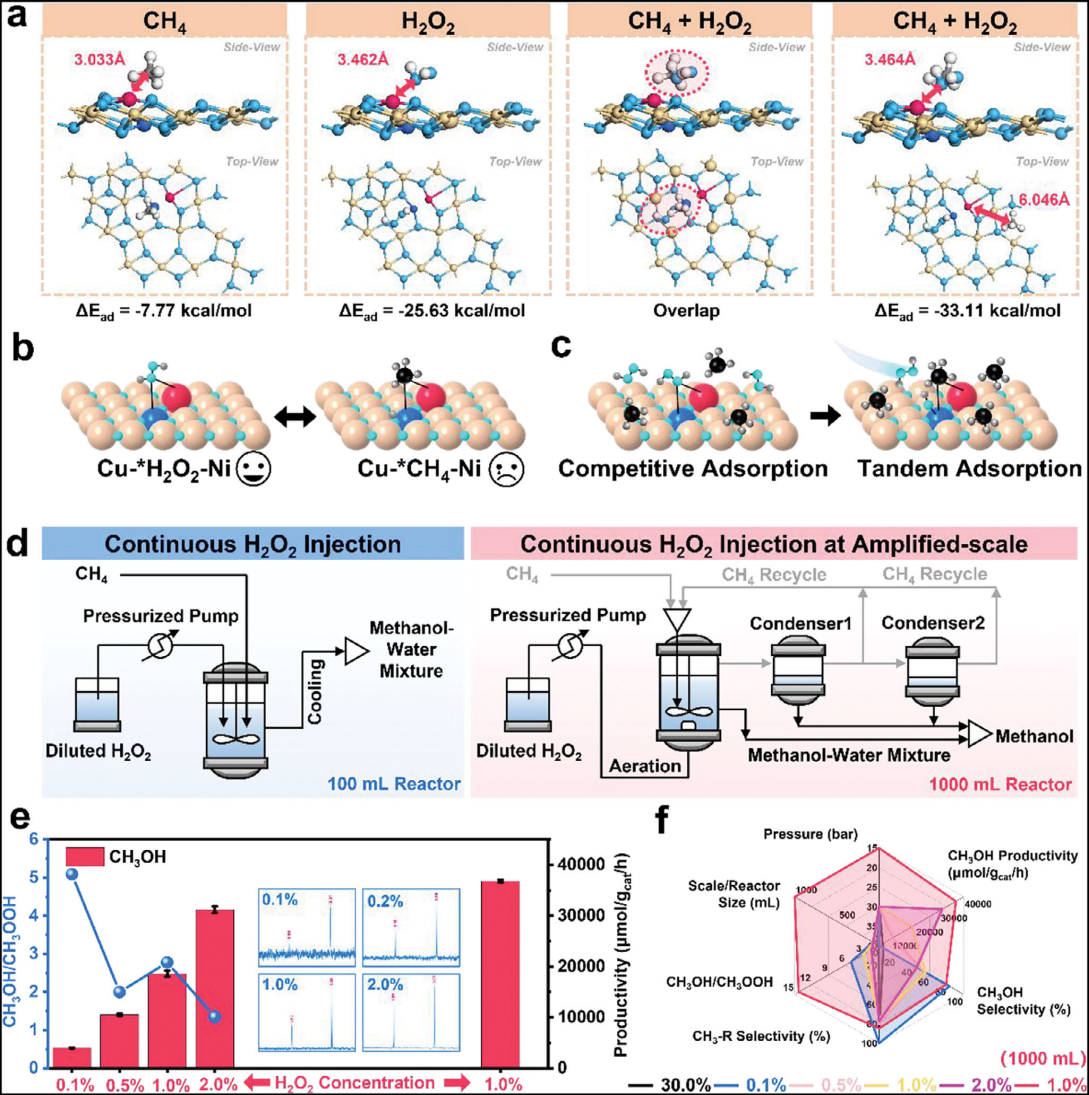
Scale‐up experiments with continuous H_2_O_2_ injection. a) CH_4_ adsorption, H_2_O_2_ adsorption, CH_4_ and H_2_O_2_ adsorption location overlap, and CH_4_+H_2_O_2_ simultaneously adsorption situations over CuNi/InNT. Scheme of b) CH_4_ and H_2_O_2_ adsorption over Cu−Ni sites, and c) altering adsorption routes of CH_4_ and H_2_O_2_. d) Technological process of continuous H_2_O_2_ injection reaction in 100 mL reactor and 1000 mL reactor. e) CH_3_OH and CH_3_OOH productivity over a continuous reactor with different H_2_O_2_ concentrations, and the insert figures were the corresponding NMR results zooming on the CH_3_OH/CH_3_OOH ratios. f) Comparative illustration of factors affecting MTM reaction.

The catalytic superiority of the CuNi/InNT system stemmed from its unparalleled ·OH radical generation capacity and optimized CH_4_ adsorption strength. Crucially, under the premise of controlling the singularity of active sites to impose inherent product selectivity, the Cu−Ni species must orchestrate as the dual‐functional sites for H_2_O_2_ dissociation and CH_4_ activation. Surface adsorption predictions indeed identified primary accumulation of both reactants (H_2_O_2_ and CH_4_) at Cu−Ni sites, with H_2_O_2_ exhibiting a markedly 3.3‐fold stronger affinity (−25.63 vs. −7.77 kcal mol^−1^) than CH_4_ (**Figure** **6**a). Optimal adsorption configurations demonstrated spatial competition between H_2_O_2_ and CH_4_ at Cu−Ni sites (Figure S48, Supporting Information). Such a pronounced adsorption preferential landscape would lead to CH_4_ being displaced to peripheral regions when simultaneously adsorbed, which was corroborated by the increased Ni‐CH_4_ distance in co‐adsorption systems (6.046 Å) versus single‐component adsorption (3.033 Å). These observations unveil a scaling relationship between ·OH generation and ^*^CH_4_ adsorption, creating a self‐limiting mechanism wherein H_2_O_2_'s dominance puts CH_4_ activation at a disadvantage (Figure 6b). The resultant ·OH surplus would drive over‐oxidation of the CH_3_OH to CH_3_OOH, significantly compromising CH_3_OH selectivity.

Building on these mechanistic insights, the strategy of maintaining Cu−Ni sites' exceptional ·OH productivity and CH_4_ activation capacity using engineering means to redirect the MTM pathway from competitive to sequential adsorption dominance was proposed (Figure 6c). The engineered continuous‐flow reactor employed a pressurized microfluidic pump to achieve precision injection of H_2_O_2_ (Figure 6d left and Figure S49, Supporting Information). Unlike conventional batch‐mode systems with bulk H_2_O_2_ dosing, this continuous design enabled steady‐state ·OH concentrations that match ^*^CH_4_ adsorption kinetics, suppressing the over‐oxidation pathways to CH_3_OOH. The 100 mL prototype achieved CH_3_OH selectivity of 83.33% at 0.1% H_2_O_2_, outperforming batch systems by 10‐fold, validating the system's exceptional capacity to suppress byproduct formation through kinetic control (Table S12, Supporting Information, entry 1). Further optimization to 1.0% H_2_O_2_ achieved an optimal balance between productivity (CH_3_OH: 18628.43 µmol g_cat_ h^−1^) and selectivity (CH_3_OH/CH_3_OOH = 2.78) (Table S12, Supporting Information, entries 2‐4), establishing these parameters as robust baselines for industrial‐scale replication.

To validate the feasibility of upgrading MTM from lab‐scale to semi‐industrial implementation, the continuous reactor was scaled up to 1000 mL with an H_2_O_2_ aeration device, a closed‐loop CH_4_ recycling system, and a cascaded methanol condensation unit (Figure 6d, right and Figure S50, Supporting Information). Through dynamic balancing of ·OH generation and ^*^CH_4_ surface coverage, the reaction pathway was redirected toward selective CH_3_OH generation instead of CH_3_OOH (Figure 6e). This semi‐industrial prototype (1000 mL) achieved a CH_3_OH yield of 36818.84 µmol g_cat_ h^−1^ with 79.37% selectivity and a CH_3_OH/CH_3_OOH ratio of 14.29, remarkably outperforming lab‐scale benchmarks at reduced energy inputs (15 bar, ΔP = ‐50%, 500 rpm) (Figure 6f, Table S12, Supporting Information, entry 5). This mechanistic‐reactor engineering hybrid approach successfully decoupled the competitive substrate adsorption, bridging atomic‐scale catalysis with macroscopic reactor design, and hence provided an operational blueprint for industrial‐scale MTM reaction.

## 3 Conclusion

In summary, Cu−Ni heteronuclear sites on defect‐abundant InNT have been prepared for MTM reaction under mild conditions, breaking through the dilemma of C−H bond polarization. Through DFT calculation analysis for spatial charge redistribution and effective overlap of atomic orbitals, copper was found to play the leading role in favoring the dissociation of H_2_O_2_ to hydroxyl radicals (·OH) and adsorbing the H in the C−H bond. While Nickel served as the electrophilic center, attracting the C end. Detailed exploration was conducted on its key advantages in appropriate orbital polarization, moderate charge transfer, and suitable Cu−Ni distance. Through the evaluation of different binary samples, the common principle of dual‐atom pair synergy was explained. And a continuous phase reactor was designed to address the disadvantage of competitive adsorption between H_2_O_2_ and CH_4_ and redirect the reaction tendency towards the tandem pathway to boost CH_3_OH yield and selectivity in an approximate industrial scenario. This work delved into the universal relevance of the catalytic reactivity of M_1_‐M_2_ binary active species with intrinsic atomic properties, charge transfer tendency, d‐orbital coupling, M_1_‐M_2_ bond strength, orbital polarization, and spin density distribution, providing a fundamental understanding for designing spatial appropriate diatomic species for efficient MTM reaction, contributing to a more sustainable and carbon‐neutral society.

## Conflict of Interest

The authors declare no conflict of interest.

## Supporting information



Supporting Information

## Data Availability

All relevant data are available from the authors on reasonable request.
