# Supplementary material for: Orchestrating Multi‐Ångstrom Spaced Cu─Ni Dual‐Atom Pair for Synergistic C─H Activation in Direct Methane Oxidation to Methanol
Source: Adv Sci (Weinh). 2025 Aug 11;12(41):e11661. doi: 10.1002/advs.202511661 (PMC12591176; doi:10.1002/advs.202511661)
Supplement: Supplementary file 1 — Supporting Information [file ADVS-12-e11661-s001.docx]

Supporting Information

Orchestrating Sub-ångstrom Spaced Cu-Ni Dual-atom Pair for Synergistic C-H Activation in Direct Methane Oxidation to Methanol

*Jingting Jin^a^, Wenzhi Li^a,b*^, Liqun Wang^a^, Lulu Zhang^c^, Xia Zhang^a^*

[Catalyst Preparation 5](#_Toc203557292)

[Catalyst Tests 5](#_Toc203557293)

[Statistical Analysis 6](#_Toc203557294)

[Characterization 7](#_Toc203557295)

[Electron Spin Resonance (ESR) spectroscopy 7](#_Toc203557296)

[In situ DRIFTS measurements 8](#_Toc203557297)

[Density functional theory (DFT) calculations 8](#_Toc203557298)

[Supplementary Note 1: Synthesis and defect-strain effect. 10](#_Toc203557299)

[Supplementary Note 2: Reasons for limited activity over NiCu@InNT. 11](#_Toc203557300)

[Supplementary Note 3: Ni’ influence on the Cu’s promotion effect for CH_3_OH and CH_3_OOH generation. 12](#_Toc203557301)

[Supplementary Note 4: Correlation analysis for experimental indicators of MTM reaction. 13](#_Toc203557302)

[Supplementary Note 5: Advantages of InNT support. 15](#_Toc203557303)

[Supplementary Figure 1: Defect engineering of InNT. 17](#_Toc203557304)

[Supplementary Figure 2: SEM images of indium supports. 18](#_Toc203557305)

[Supplementary Figure 3: Contact Angle of Solid-liquid Gas Interface and results of N_2_-adsorption-desorption experiments over In_2_O_3_ and InNT. 20](#_Toc203557306)

[Supplementary Figure 4: Morphology of InNT. 21](#_Toc203557307)

[Supplementary Figure 5: GPA analysis for InNT. 23](#_Toc203557308)

[Supplementary Figure 6: In situ ESR spectrum of In_2_O_3_ and InNT. 24](#_Toc203557309)

[Supplementary Figure 7: liquid phase products analysis of InMOF. 26](#_Toc203557310)

[Supplementary Figure 8: Overall comparison of different catalysts. 27](#_Toc203557311)

[Supplementary Figure 9: Altering loading order for different reactions. 28](#_Toc203557312)

[Supplementary Figure 10: The XRD patterns of metal catalysts. 30](#_Toc203557313)

[Supplementary Figure 11: The In_2_O_3_ (222) peak deviation of catalysts. 31](#_Toc203557314)

[Supplementary Figure 12: Active metals loading evidence of In catalysts.. 32](#_Toc203557315)

[Supplementary Figure 13: Cu and Ni 2p 3/2 spectrum of InNT catalysts. 34](#_Toc203557316)

[Supplementary Figure 14: Raman data of InNT catalysts. 35](#_Toc203557317)

[Supplementary Figure 15: Raman proof for the existence of In^x+^. 37](#_Toc203557318)

[Supplementary Figure 16: FT-IR spectrum of In_2_O_3_ InNT and CuNi/InNT. 38](#_Toc203557319)

[Supplementary Figure 17: NO-DRIFTS results of catalysts. 39](#_Toc203557320)

[Supplementary Figure 18: Theoretical structure diagram of In_2_O_3_ and InNT. 41](#_Toc203557321)

[Supplementary Figure 19: Theoretical structure diagram of Cu/InNT. 43](#_Toc203557322)

[Supplementary Figure 20: Theoretical structure diagram of Ni/InNT. 44](#_Toc203557323)

[Supplementary Figure 21: Charge density difference diagram, EPR, and UV-Vis-DRS differences of Cu/InNT and Ni/InNT. 45](#_Toc203557324)

[Supplementary Figure 22: EIF results of Cu/InNT. 48](#_Toc203557325)

[Supplementary Figure 23: Possible theoretical structure diagram of CuNi/InNT and NiCu/InNT. 49](#_Toc203557326)

[Supplementary Figure 24: Theoretical structure diagram of InNT-based catalysts. 50](#_Toc203557327)

[Supplementary Figure 25: The O_2_ generation ability of CuPd/InNT, CuZn/InNT, and CuCu/InNT. 51](#_Toc203557328)

[Supplementary Figure 26: The d-orbital hybridization results of CuPd/InNT, CuZn/InNT, and CuCu/InNT. 52](#_Toc203557329)

[Supplementary Figure 27: The 3d-orbital situations of CuPd/InNT, CuZn/InNT, and CuCu/InNT. 53](#_Toc203557330)

[Supplementary Figure 28: Calculated spin density results of CuPd/InNT, CuZn/InNT, CuZn/InNT, and CuNi/InNT. 54](#_Toc203557331)

[Supplementary Figure 29: The partial CH_4_-DRIFTS results of samples. 55](#_Toc203557332)

[Supplementary Figure 30: The overall CH_4_-DRIFTS results of samples. 57](#_Toc203557333)

[Supplementary Figure 31: Free radical quenching experiments. a, yield comparison of free radical quenching experiments on InNT and CuNi/InNT. 58](#_Toc203557334)

[Supplementary Figure 32: The d-band centers of InNT catalysts. 60](#_Toc203557335)

[Supplementary Figure 33: In-depth correction analysis. 61](#_Toc203557336)

[Supplementary Figure 34: Charge density difference diagram of CuNi/InNT. 62](#_Toc203557337)

[Supplementary Figure 35: The orbital situations of CuNi/InNT in step ii. 63](#_Toc203557338)

[Supplementary Figure 36: The calculated differential charge density, spin density and orbital difference of O in Cu-OH-Ni and surface adsorbed *OH in step ii. 64](#_Toc203557339)

[Supplementary Figure 37: The CH_4_ affinity with or without *OH from H_2_O_2_ decomposition and the overlap situation of C-2p and H-1s total DOS in step v. 65](#_Toc203557340)

[Supplementary Figure 38: a, the charge density difference using *CH_3_ as set in step vii. b, the spin density results of *CH_3_. Blue represents obtaining electrons, yellow represents donating electrons. 66](#_Toc203557341)

[Supplementary Figure 39: The d-band shift of Cu and Ni in steps vii and viii. 67](#_Toc203557342)

[Supplementary Figure 40: The following reaction steps of CH_3_OH and CH_3_OOH.. 68](#_Toc203557343)

[Supplementary Figure 41: The charge density difference using *CH_3_OOH as set. 69](#_Toc203557344)

[Supplementary Figure 42: O-2p orbital situations of *CH_3_OH and *CH_3_OOH. 70](#_Toc203557345)

[Supplementary Figure 43: Calculated bond length of *CH_3_OH and *CH_3_OOH. 71](#_Toc203557346)

[Supplementary Figure 44: Illustration of involved intermediates. 72](#_Toc203557347)

[Supplementary Figure 45: Illustration of all reaction pathways. 73](#_Toc203557348)

[Supplementary Figure 46: Illustration of CH_4_ dissociation over InNT. 74](#_Toc203557349)

[Supplementary Figure 47: Calculated dissociation of H_2_O_2_ over InNT and CuNi/InNT. 75](#_Toc203557350)

[Supplementary Figure 48: The adsorption location field of CH_4_, H_2_O_2_, and CH_4_+H_2_O_2_ over CuNi/InNT. 76](#_Toc203557351)

[Supplementary Figure 49: The photograph of 100 mL continuous H_2_O_2_ injection reactor. 77](#_Toc203557352)

[Supplementary Figure 50: The photograph of 1000 mL continuous H_2_O_2_ injection reactor. 78](#_Toc203557353)

[Supplementary Table 1. Detailed catalytic performance of In-based catalysts 79](#_Toc203557354)

[Supplementary Table 2. Catalytic performance of MTM conversion over recently reported promising catalysts in the liquid phase. 83](#_Toc203557355)

[Supplementary Table 3 Crystallinity and Strain of Various Catalysts 85](#_Toc203557356)

[Supplementary Table 4 Surface Concentrations of Elements on Various Catalysts 86](#_Toc203557357)

[Supplementary Table 5 Detailed NO-DRIFTS Peak Position of Indium catalysts 88](#_Toc203557358)

[Supplementary Table 6 Calculated Mulliken Charge of Different Configurations 89](#_Toc203557359)

[Supplementary Table 7 Calculated Formation Energy of Different Configurations 90](#_Toc203557360)

[Supplementary Table 8 Detailed In-situ DRIFTS Peak Position of Indium Catalysts 91](#_Toc203557361)

[Supplementary Table 9 Calculated Atom Distance of Different Configurations 92](#_Toc203557362)

[Supplementary Table 10 Stress Tensor of Different Configurations 93](#_Toc203557363)

[Supplementary Table 11 Calculated Mulliken Charge of Different Configurations during Reaction 94](#_Toc203557364)

[Supplementary Table 12. Detailed catalytic performance of Continuous H_2_O_2_ Injection Reactions 95](#_Toc203557365)

[Reference. 96](#_Toc203557366)

**Catalyst Preparation**

CuNi/InNT catalyst was prepared via a step-wise photo-deposition method. 100 mg of InNT support was suspended in 95 mL of distilled water with 5 mL of ethanol as sacrificial agent in a photochemical reactor with a quartz window. 382 μL of 50 mM CuCl2 was added into the solution. After light irradiation with 500 W ultraviolet mercury lamp (main wavelength range of 315-450nm and dominant wavelength of 365nm) for 3 hours, another 382 μL of 50 mM Ni(NO_3_)_3_ was also included in the reactor. Then, the slightly grayed sample was collected by suction filtration and dried at 80 °C overnight. During each step of the preparation, 250 rpm of stirring was need and the reactor chamber was sealed and certain amount of argon was introduced to evacuate the air. All procedures involving light irradiation were performed with circulating water to keep the reaction temperature at 25 °C to avoid premature aggregation and denaturation of precursor metal salts. The sample naming followed deposition order, as Cu/InNT and Ni/InNT were deposit only one metal, NiCu/InNT was to load Ni first and Cu later, NiCu@InNT was to put both Ni and Cu precursors at only one light irradiation procedure. Note that preparation processes for InMOF and InNT were given in supplementary note 1.

**Catalyst Tests**

The catalytic performance of as-synthesized samples was evaluated *via* a 45 mL four-station parallel reactor from Guizhou Shanli Experimental Instrument Co., Ltd. Typically, 5 mg catalyst was suspended in 19 mL H_2_O. Then 1 mL of 30 vol% H_2_O_2_ was also added. Subsequently, the reactor was sealed then purged for 3 times and charged to 3.0 MPa with 95% CH_4_ balanced with 5% Ar. The batch reactor was heated to 70 °C and maintained for 30 min under stirring (800 rpm). After reaction process, the reactor was instantly cooled with ice-bath to avoid the loss of CH_3_OH. In addition, the detailed parameters of continuous reaction in 100 mL reactor were described accordingly in the supplementary figure legends.

Gas-phase products were collected into an airbag to be further analyzed *via* a gas chromatograph (GC) equipped with a Thermal Conductivity Detector (TCD). The liquid phase products were determined on a Bruker JNM-ECZ600R/S1 600 MHz NMR instrument. Specifically, the CH_3_OH was quantified *via* GC 5190PLUS with HP-PLOT/Q column and flame ionization detector (FID) from Anhui Chromatography Instrument Co., Ltd. CH_3_OH yield and other indicators were calculated using the following equations.

${CH}_{3}OH yield (\mu mol/g_{cat}/h)=\frac{{CH}_{3}OH Amount (\mu mol)}{Catalyst Amount \left( g \right)\times Reaction Time (h)}$ …… [1]*,*

$Methyl Oxygenates Selectivity \left( \% \right)=\frac{n\left[ {CH}_{3}OH+{CH}_{3}OOH \right]}{n\left[ all liquid products \right]} \times100\%$ …… [2],

${CH}_{4}Conversion Rate (\%)=\frac{{n\left[ {CH}_{4} \right]}_{Begin}- {n\left[ {CH}_{4} \right]}_{End}}{{n\left[ {CH}_{4} \right]}_{Begin}} \times100\%$ …… [3].

$Turnover frequency (TOF)=\frac{CH3-R yield (\mu mol/gcat/h)}{Density of Cu-Ni dual atom pair}$ …… [4].

**Statistical Analysis**

Experimental data was used without pre-processing. The presentation of the experimental data is to average two or more sets of data and the variance was reflected in the form of error bars. In correlation analysis, Cu/InNT, Ni/InNT, NiCu@InNT, NiCu/InNT and CuNi/InNT’s relevant experimental data, characterization results, and theoretical calculation conclusions were analyzed, with a p-value of 0.05, and the software used was Origin.

**Characterization**

Images of sample morphology were captured by ZEISS Gemini SEM 500 scanning electron microscope (SEM) and JEM 2100F field emission transmission electron microscope (TEM). Element mapping images and EDS energy analysis results were obtained. Surface crystal structures were obtained through high-resolution TEM (HRTEM) by JEM-F200. X-ray diffraction patterns (XRD) were tested on a Rigaku TTP-III power X-ray diffractometer in the range of 5 °~80 ° with a step size of 0.02 °. Surface metal species were characterized based on UV-visible diffuse reflectance (UV-vis DRS) spectra performed on SOLID 3700 in the range of 190-1100 nm with BaSO_4_ as a reference. Electron Spin Resonance (ESR) Spectra of JEOL JES-FA200 ESR was employed to detect defect situations of the samples. X-ray photoelectric spectroscopy (XPS) was conducted on a Thermo ESCALAB250Xi spectrometer using an Al-Kα source. And the values of binding energies of desired elements were calibrated with the adventitious C 1s peak at 284.80 eV. Raman spectrum was detected by LabRAM HR Evolution. Fourier transform infrared reflection (FT-IR) were applied on a Niolet iS50 FT-IR spectrometer with a DTGS KBr detector. Nitrogen adsorption-desorption results were collected over a Micromeritics ASAP 2460 instrument.

**Electron Spin Resonance (ESR) spectroscopy**

Photo response of samples was tested by in situ ESR with xenon lamp. During the experiments, signals of narrow and wide spectrum were collected under dark condition, 30 seconds, 120 seconds and 240 seconds, respectively. ESR was also employed for the detection of free radicals. Typically, 1 mL 100 mmol/L 5, 5′-dimethyl-1-pyrroline-N-oxide (DMPO) solution was mixed with 1 mL reaction solution. Then the mixture was immediately frozen by liquid nitrogen to prolong the transient lifetime of DMPO-OH and DMPO-OOH for transportation. The frozen sample was defrosted to RT and rapidly transferred into a capillary tube for testing. The detection parameters were controlled to be the same over all samples to avoid any non-experimental deviations.

**In situ DRIFTS measurements**

The in situ diffuse reflectance infrared Fourier transform spectroscopy was carried out using the same instrument with FT-IR equipped with a MCT detector. Constant amount of sample powder was packed into the reaction chamber to avoid non-experimental deviations. Then the background spectrum was collected for 32 scans at a resolution of 4 cm^-1^. Spectrums with gas infeed of 15 % CH_4_ balanced with N_2_ were recorded at temperatures from 25 to 70 °C (or 10% NO balanced with N_2_ at 25 °C for NO-DRIFTS). In addition, Ar purge experiment was conducted with fully CH_4_ adsorbed samples to assess the adsorption strength of CH_4_ on catalysts. All gas infeed was kept at 2 mL/min.

**Density functional theory (DFT) calculations**

Catalyst’s first-principle density functional theory (DFT) calculations were conducted within the CASTEP software package^1^. The slab model was separated from adjacent layers of In_2_O_3_ (222) by a vacuum with a thickness of 15 Å. For geometry optimization, we employed The Perdew–Burke–Ernzerhof method (PBE) within generalized gradient approximation (GGA) functional^2^. The cut-off energy for plane wave expansion is 570 eV. An energy convergence threshold of 1.0 × 10^-5^ eV and a force configuration tolerance of 0.03 eV/Å were used to obtain the optimum geometry configuration. Dmol3 software package was used for the analysis of COOP and COHP with PBE and GGA. Dmol3 was also used to calculate the Fukui function with a 1 × 1 × 1 Gamma k-point mesh with Becke-Lee-Yang-Parr (BLYP) within generalized gradient approximation (GGA) functional. Adsorption locater software package was used for the analyzing the competitive adsorption situation of CH_4_ and H_2_O_2_ molecular over CuNi/InNT surface (define the outer surface layer atoms as the adsorption target) with the universal forcefield type, Ewald & Group electrostatic and atom-based van der waals.

**Supplementary Note 1: Synthesis and defect-strain effect.**

A MOF-driven template method to achieve defect engineering of indium oxide. MIL-68(In) (InMOF) was prepared inspired by formally reported methods. Typically, 7.6 mmol of In(NO_3_)_3_·xH_2_O was dissolved in 12.4 mL of N, N-Dimethylformamide (DMF), noted as solution A, and 2.6 mmol of 1,4-dicarboxybenzene (TPA) was also dissolved in 12.4 mL of DMF note as solution B. After the precursors were fully dissolved, the two solutions were mixed together and thoroughly stirred again, then the mixed solution was put into a 50 mL autoclave and kept at 100 °C for 24 hours. After centrifugation and washed repeatedly with deionized water and methanol, the insoluble white solid was dried in an oven at 60 °C for 24 hours to remove remaining water and organic solvent impurities. Indium oxide nanotube (InNT) was synthesized by directly calculating InMOF materials. Firstly, it was put in a vacuum at 150 °C for 2 hours to remove guest molecules within the channels, then the sample was calcined at 500 °C for 2 hours at a ramping speed of 5 °C/min.

During calculation, the organic part of MOF will be evaporated leaving only metal and oxygen atoms (and a trace amount of C). During the formation of the In_2_O_3_ crystal structure, there will be some overlapping or fusing among small particles for their growth is always irregular which would cause both stretched and compressed straining over crystals. Eventually, the presence of irregular growth and strain promoted defect formation (including oxygen defects and metal cation defects over the surface and bulk phase), affecting the light absorption range and altering the light excitation response behavior.

**Supplementary Note 2: Reasons for limited activity over NiCu@InNT.**

It was found that, unlike the common bimetallic modification that improves reaction performance, the overall performance of the simultaneously loaded NiCu@InNT sample was the least active, and even more sluggish than pure carriers. However, its CH_3_OH yield was higher than InNT, Cu/InNT, and Ni/InNT, indicating that Cu and Ni still had some interaction with each other. Especially the significant increase in the CH_3_OH/CH_3_OOH ratio suggested that the active sites might still be bimetallic and those sites can also lead to the generation of ·OH and ·OOH.

This phenomenon can be attributed to the reduction of effective sites caused by competitive adsorption of Cu and Ni during photo-deposition. The calculated formation energy concluded that the loading of Cu and Ni single metal species in the first step was relatively spontaneous. Therefore, when both precursor salt solutions were present simultaneously, Cu and Ni would compete with each other, resulting in the difficulty in the formation of key Cu species that facilitate the dissociation of H_2_O_2_ into ·OH, consisting with XPS’s lowest Cu loading in NiCu@InNT. Moreover, the energy barrier of loading single Cu followed by loading Ni was significantly lower than the route of loading Ni first. When both salts were added, the loading of Cu would be hindered, resulting in fewer Cu-Ni diatomic sites like in CuNi/InNT with more Ni clusters like in Ni/InNT. Therefore, the catalyst may be coupled by Cu-Ni diatoms and Ni clusters, while Ni clusters were not conducive to the generation of ·OH. The experiment found that this sample produced the most O_2_, indicating that this configuration favored H_2_O_2_ decomposition to O_2_, thus resulting in the lowest CH_3_-R yield.

**Supplementary Note 3: Ni’ influence on the Cu’s promotion effect for CH_3_OH and CH_3_OOH generation.**

During experiments, it was found that single metal modifications have different impacts on CH_3_-R (CH_3_OH and CH_3_OOH) generation, which was that Cu showed a promotive effect while Ni presented inhibitory effect. It is reasonable to assume that if there were no or only merely interactions between Cu-Ni, the CH_3_-R yield in bimetallic samples will be similar to that of pure carriers (InNT), as the impact of Cu and Ni might cancel each other out. However, the actual situation is that NiCu/InNT and NiCu@InNT showed a further decrease, while CuNi/InNT showed a significant CH_3_-R productivity improvement. In order to further investigate the specific mechanism of Cu and Ni on the generation of CH_3_-R in their simultaneous presence, we compared the CH_3_-R yields of different catalysts as following.

$$Level of impact (\%)=\frac{{\left[ {CH}_{3}-R \right]_{Catalyst 2}-\left[ {CH}_{3}-R \right]}_{Catalyst 1}}{\left[ {CH}_{3}-R \right]_{Catalyst 1}} \times100\%$$

It could be concluded that the presence of Cu would suppress the inhibiting effect of Ni while Ni could enhance the promotion effect of Cu on CH_3_-R generation (Cu/InNT - InNT = 5.3%, Ni/InNT - InNT = -22.0%, CuNi/InNT - Cu/InNT = 91.3%, NiCu/InNT - Ni/InNT = 16.8%). It is once again confirmed that there may be special interactions between Cu-Ni that are favorable for CH_3_-R production, especially in CuNi/InNT where there might be a special configuration of Cu-Ni, which greatly enhances their synergistic effect.

**Supplementary Note 4: Correlation analysis for experimental indicators of MTM reaction.**

To elucidate the detailed MTM mechanism over indium-based catalysts, CH_4_-DRIFTS provided insights into the adsorption behavior of CH_4_ and related intermediates, and EPR offered information on free radical generation. However, upon analyzing these multiple indicators, it was found that the optimal CuNi/InNT catalyst did not excel in all aspects. Therefore, correlation analysis was performed to comprehensively analyze various indicators and integrate catalyst configuration information^3-5^. The study encompassed three sections: catalyst configuration (types of active metals, active metal ratios, and defect conditions), intermediate performance indicators (CH_4_ adsorption response, CH_4_ adsorption intensity, ·OH, ·OOH), and product outcomes (CH_3_OH, CH_3_OOH, by-products) to investigate the impact of catalyst configuration on the reaction pathways and provide a summary of the structure-activity relationships.

For the relationship between catalyst configuration and intermediates, it was found that Cu enhanced the adsorption strength of CH_4_, while Ni has the opposite effect, particularly showing a significant negative correlation with adsorption intensity. It could be inferred that the presence of both metals may complement each other. In terms of free radical generation, Cu favored the production of ·OH and ·OOH radicals, whereas Ni was detrimental to radical formation. Thus, Cu-Ni bimetallic modification may be beneficial for improving CH_4_ adsorption characteristics but not favorable for free radical generation. Interestingly, experiments revealed a high yield of ·OH radicals in CuNi/InNT. Subsequently, using XPS semi-quantitative results, a new indicator, the Cu/Ni ratio, was generated for analysis, showing that this ratio primarily benefited CH_4_ adsorption and ·OH production. That is, in the presence of Cu, the negative impact of Ni on free radical generation would be suppressed. Additionally, contrary to common understanding, the presence of O defects did not significantly correlate with free radical production, but the presence of In defects significantly favored the generation of ·OOH radicals, suggesting that ·OOH radicals may preferentially form on InNT.

Significantly, those intermediates also showed interlinkages. Judging from experimental results of the increase in H_2_O_2_ consumption in the N_2_ gas blank control, CH_4_ adsorption and H_2_O_2_ consumption were negatively correlated, indicating a shared active site for both processes. And the correction results also showed slight negative relationship of these factors. ·OOH and CH_4_ adsorption intensity were positively correlated, considering the defect-rich surface of InNT catalysts, suggesting that H_2_O_2_ may be adsorbed preferentially before CH_4_ adsorption. This implied that the presence of ·OOH enhanced the adsorption of CH_4_, and the processes of CH_4_ adsorption and H_2_O_2_ decomposition were likely to occur at the same site, with H_2_O_2_ adsorbed before CH_4_.

For intermediates and products, it was revealed that CH_3_OH and CH_2_(OH)_2_ were positively correlated, and so as CH_3_OOH and HO(CH_2_O)_n_, indicating that CH_2_(OH)_2_ was the over-oxidation product of CH_3_OH, and HO(CH_2_O)_n_ was that for CH_3_OOH. Additionally, an increase in ·OH radicals corresponded with higher CH_3_OOH levels, further suggesting that ·OH may be one of the precursors to CH_3_OOH.

**Supplementary Note 5: Advantages of InNT support.**

InNT exhibited multiple advantages compared to In_2_O_3_. When used as a carrier, InNT possessed a larger surface area which led to a more sufficient response to light irradiation. As evidenced by UV-Vis-DRS in Fig. S21, the bandgap of In_2_O_3_ was 2.53 eV, while the bandgap of InNT decreased to 2.24 eV. This was due to residual C during the InMOF calcination process^6, 7^.

When excited by light, electrons can transition to the conduction band through newly introduced impurity energy levels that act as a springboard and reduce the energy required to excite electron-hole pairs^8^. The defects over InNT also contributed to bandgap reduction. The generation of bulk phase V_O_ will cause electronic localization at their positions, which directly affects the electronic structure of the semiconductor. In addition, the generation of oxygen vacancies can also create donor energy levels near the bottom of the conduction band in the semiconductor bandgap, reducing the bandgap width. HOMO-LOMO calculations found that the V_In_ and V_O_ defect sites were electron-rich areas under light, providing precise anchoring points for active metals^9-11^. The neighboring V_In_ and V_O_ defects also provided wider space for constructing diatomic sites. In addition, the abundant metal vacancies and oxygen vacancies in InNT can cause changes in the electronic arrangement of adjacent metal elements.

When used as catalyst, InNT was superior due to its enhanced hydrophobicity that reduced the unwanted H_2_O occupation of active sites and facilitated the general contact with H_2_O_2_ and CH_4_. Meanwhile, InNT exhibited lattice strain which would affect the d-band center, thereby affecting the adsorption of substrates^12, 13^. The energy barrier for InNT to adsorb and dissociate H_2_O_2_ during the MTM reaction was -2.28 eV, indicating that the unique neighboring defect configuration of V_In_ and V_O_ was conducive to ·OOH production, thus enhancing the CH_3_-R yield of the MTM reaction.

In summary, InNT’s unique configuration of In-O defects and crystal mismatch would promote photo-response behavior, maximizes the synergistic effect between active metals and carriers, and eventually improving CH_4_ conversion efficiency. Therefore, InNT was a more suitable carrier for Cu-M heteronuclear sites construction than In_2_O_3_.


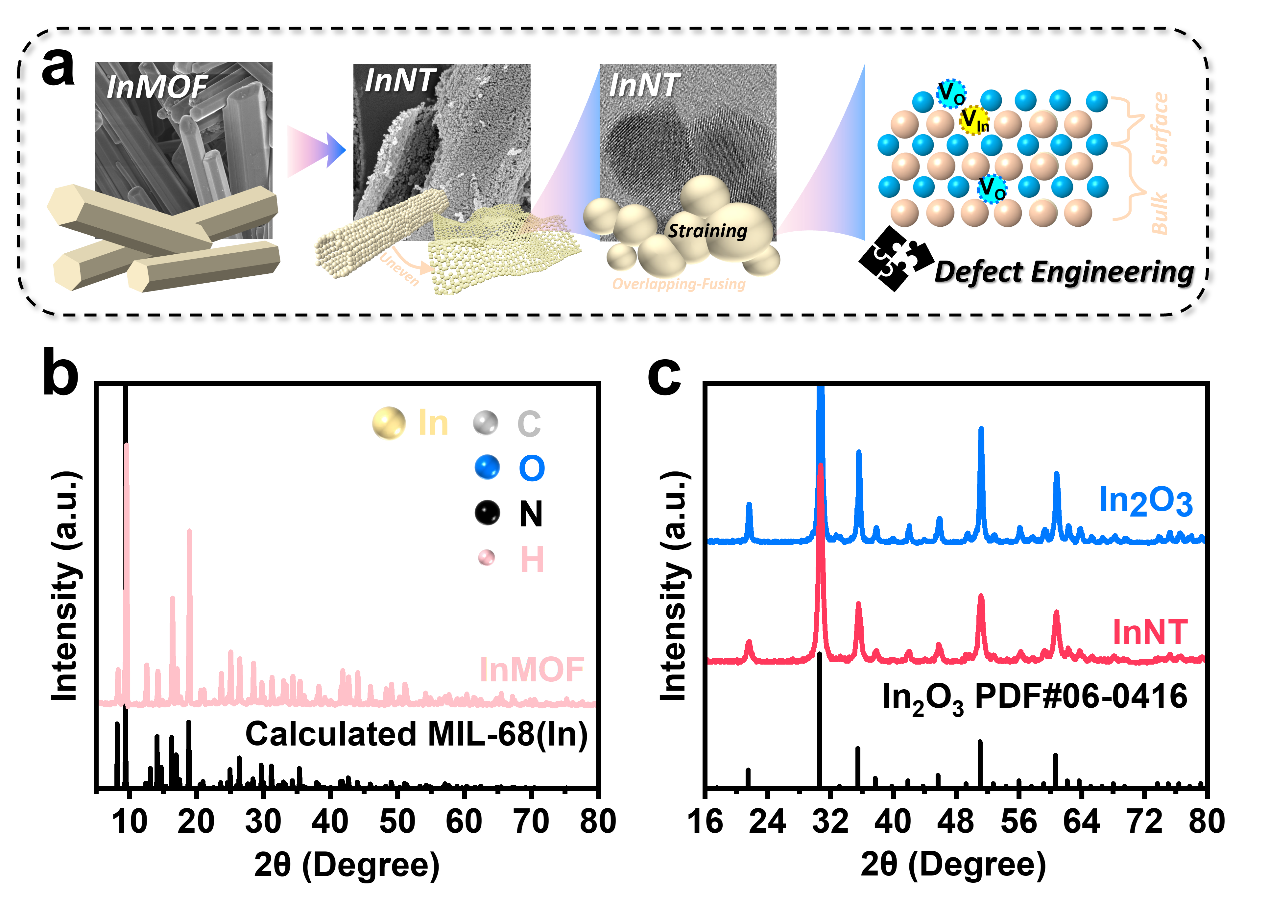


**Supplementary Figure 1: Defect engineering of InNT. a**, schematic process of defect engineering of InNT. **b**, XRD patterns of calculated MiL-68(In) and synthesized InMOF. **c**, XRD patterns of commercial In_2_O_3_ (Shanghai Aladdin Biochemical Technology Co., Ltd), synthesized InNT and a standard In_2_O_3_ PDF card data as reference.

The synthesized InMOF’s XRD pattern showed great consistency with the simulated MIL-68(In) (Fig. 1b). In addition, the prepared InNT’s XRD pattern was also in decent consistency with commercial In_2_O_3_ and the standard In_2_O_3_ PDF card data (Fig. 1c), suggesting InMOF and InNT was successfully obtained.


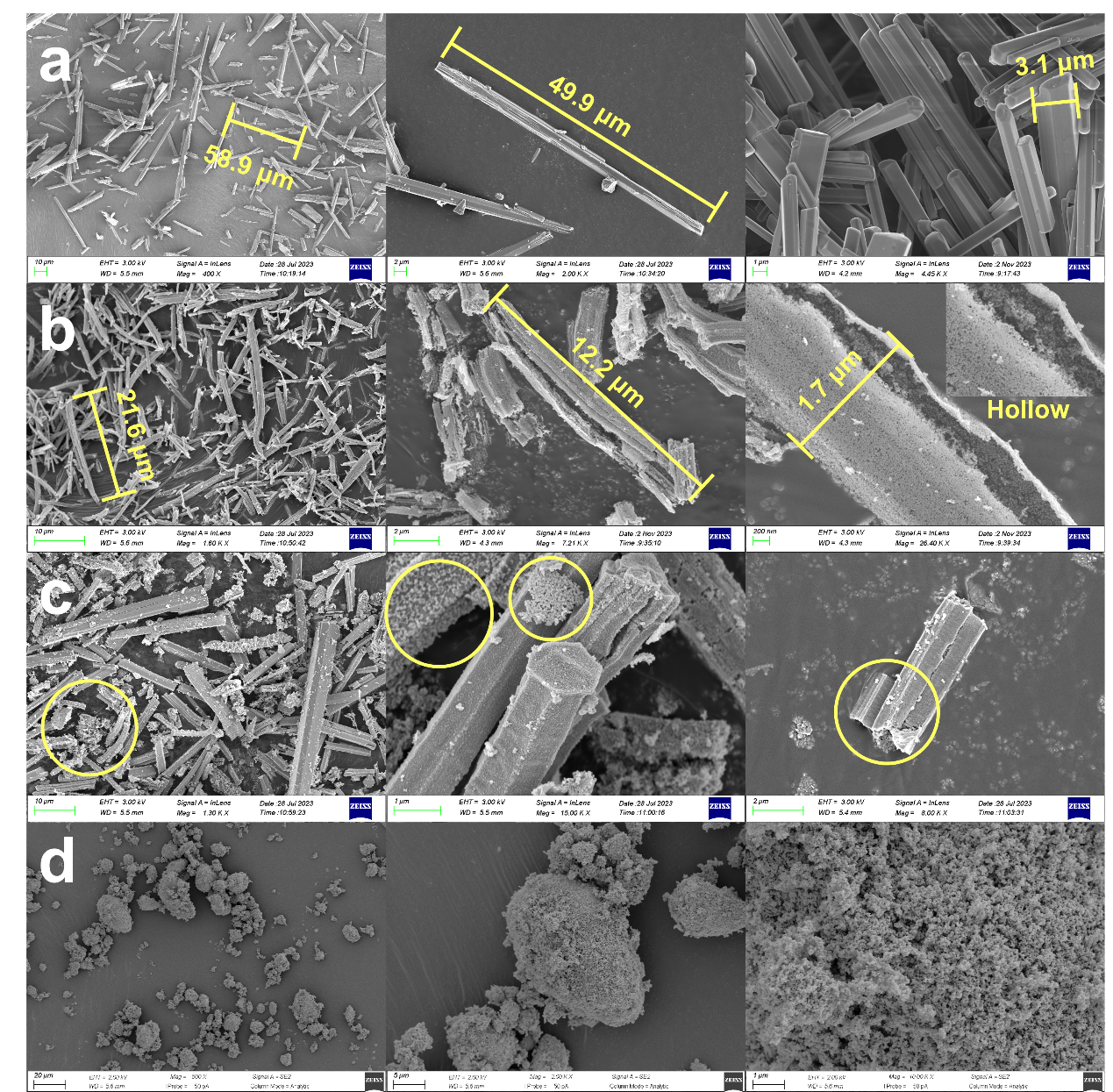


**Supplementary Figure 2: SEM images of indium supports. a**, InMOF. b, InNT. c, InNT-covered. d, Commercial In_2_O_3_.

The InMOF was in the prismatic structure, with hexagonal prisms accounting for the majority, ranging from 1 to 3 microns in length and around 20 to 50 microns in length despite being slightly fragmented. The In_2_O_3_ formed after calcination maintained its hexagonal prism morphology, but its diameter and length were both reduced (1-2 microns, 10-20 microns) due to the removal of organic ligands, resulting in the column body became rough from smooth and the center became hollow. When magnified, it could be seen that the sample has been converted into tube-like structure composed of multiple In_2_O_3_ microspheres. Therefore, it was named InNT (InMOF-based nanotube). To observe the process of transformation from InMOF to InNT more intuitively, we covered parts of the ceramic boat with tin foil during calcination (named as the InNT-covered sample), creating a temperature gradient inside the porcelain boat. As shown in the yellow circle in the figure, the transition from the smooth surface of InMOF to In_2_O_3_ microspheres could be seen. As to the commercial In_2_O_3_ sample, no obvious morphology can be spotted.


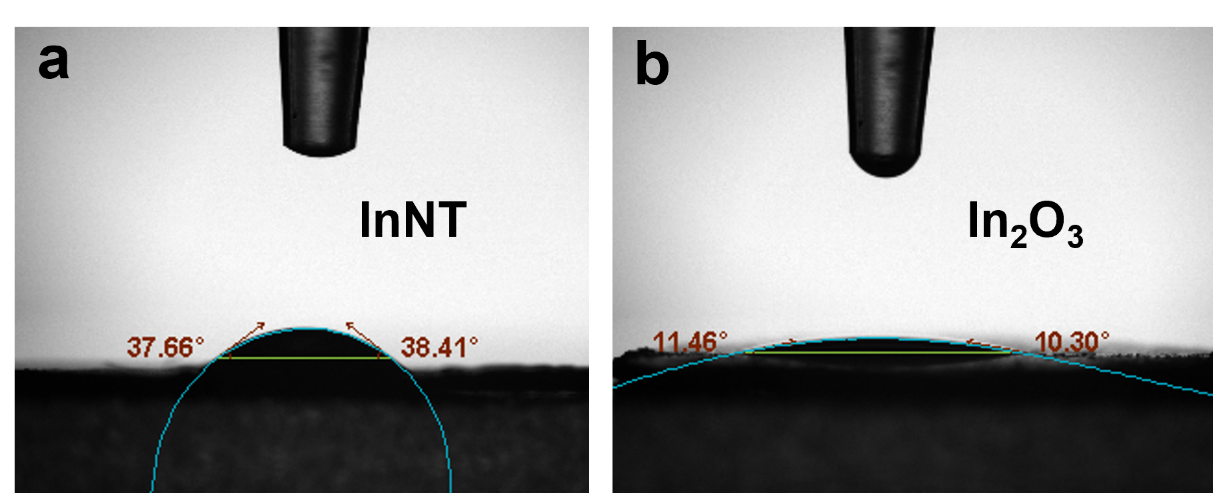


**Supplementary Figure 3:** Contact Angle of Solid-liquid Gas Interface a, InNT. b, In_2_O_3_ and results of N_2_-adsorption-desorption experiments over In_2_O_3_ and InNT.

The samples were compressed into thin sheets, and then H_2_O was dropped onto these sheets. The contact processes were filmed, and the two selected images were at the same time stamp. It is clear that InNT was more hydrophobic that is to say it has a lower affinity for water, which was beneficial for improving reaction efficiency in heterogeneous catalysis.

The N_2_-adsorption-desorption experiments showed the BET surface area of InNT was 39.8758 m^2^/g, which presented a 17% increase compared to the In_2_O_3_ sample (Figure 3c). This was due to MOF driven InNT has a microsphere structure that would increase surface area, different from the commercial In_2_O_3_ with no specific morphology. Analysis of the pore size revealed that InNT exhibited a specific peak signal around 7.2 nm, indicating the presence of certain microporous features, which was also attributed to the microsphere structure of InNT (Figure 3d).


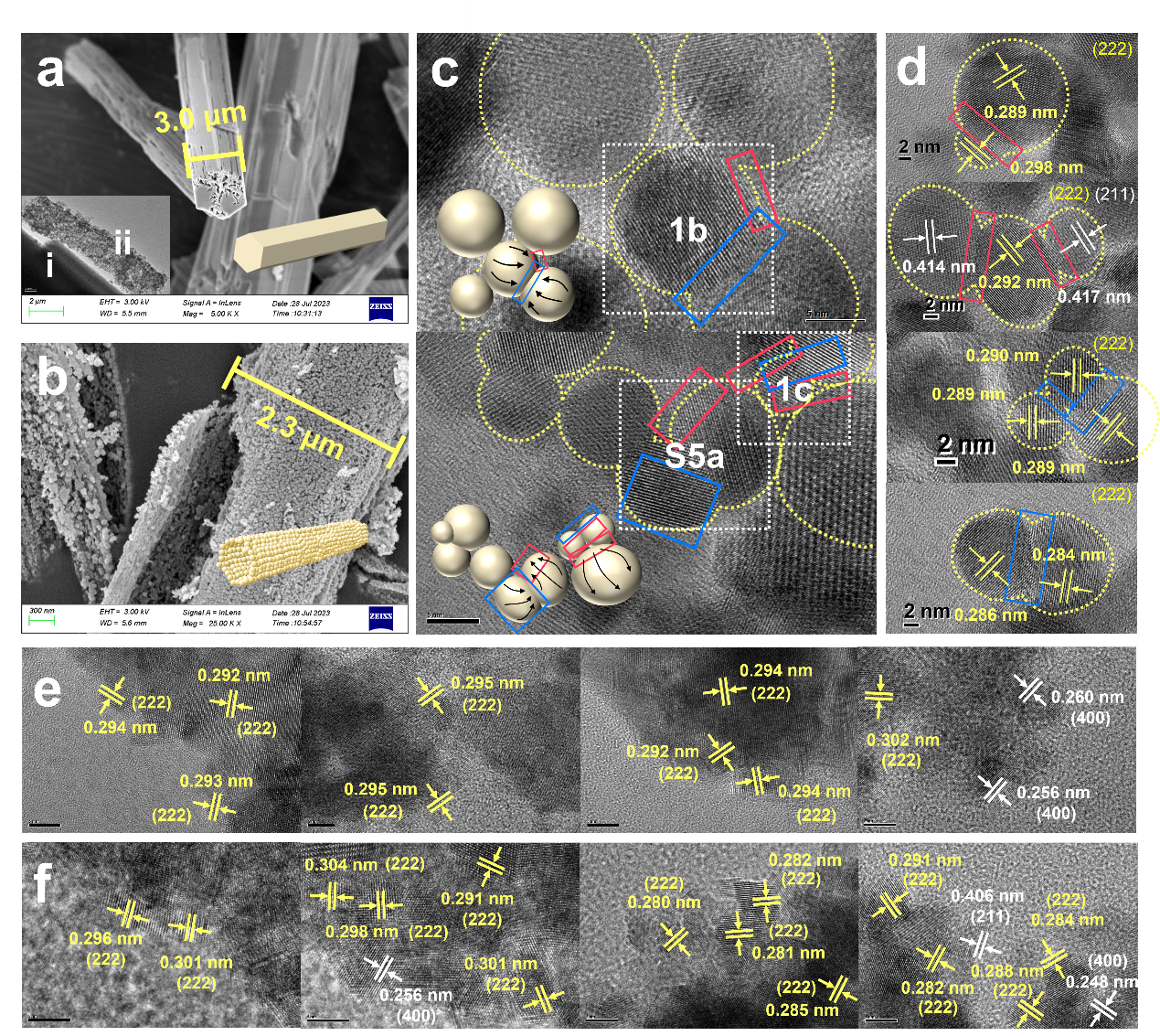


**Supplementary Figure 4: Morphology of InNT. a**, SEM of InMOF, the inserted figure was the HRTEM image of InNT-covered with i for InMOF and ii for InNT. **b**, SEM and of InNT. **c-f**, HRTEM of InNT. The white box framed 1b and 1c was also included in Figure 1 and Figure S5.

The general morphology of InMOF was a hexagonal prism and the nanotube structure could also be validated (Fig, S4a-b). The InNT sample mostly exposed the (222) facet with both compressed and stretched strain (especially at the junctions) between nanospheres, this is due to the stacking error during the transformation of InMOF to InNT which would produce more In and O defects (Fig, S4c-d)^14^. GPA analysis gave a more direct view of the stretching and compressed strain at the interface of nanospheres (Fig, S4e), and similar results are observed on multiple crystal planes (222), (400), and (440). According to PDF card data, the standard facet spacing of (222) is 0.292 nm, which has changed to 0.284-0.298 nm in InNT. Similarly, for the (400) facet, it changed from 0.253 nm to 0.242-0.254 nm, and for the (440) facet, it changed from 0.179 nm to 0.176-0.180 nm.


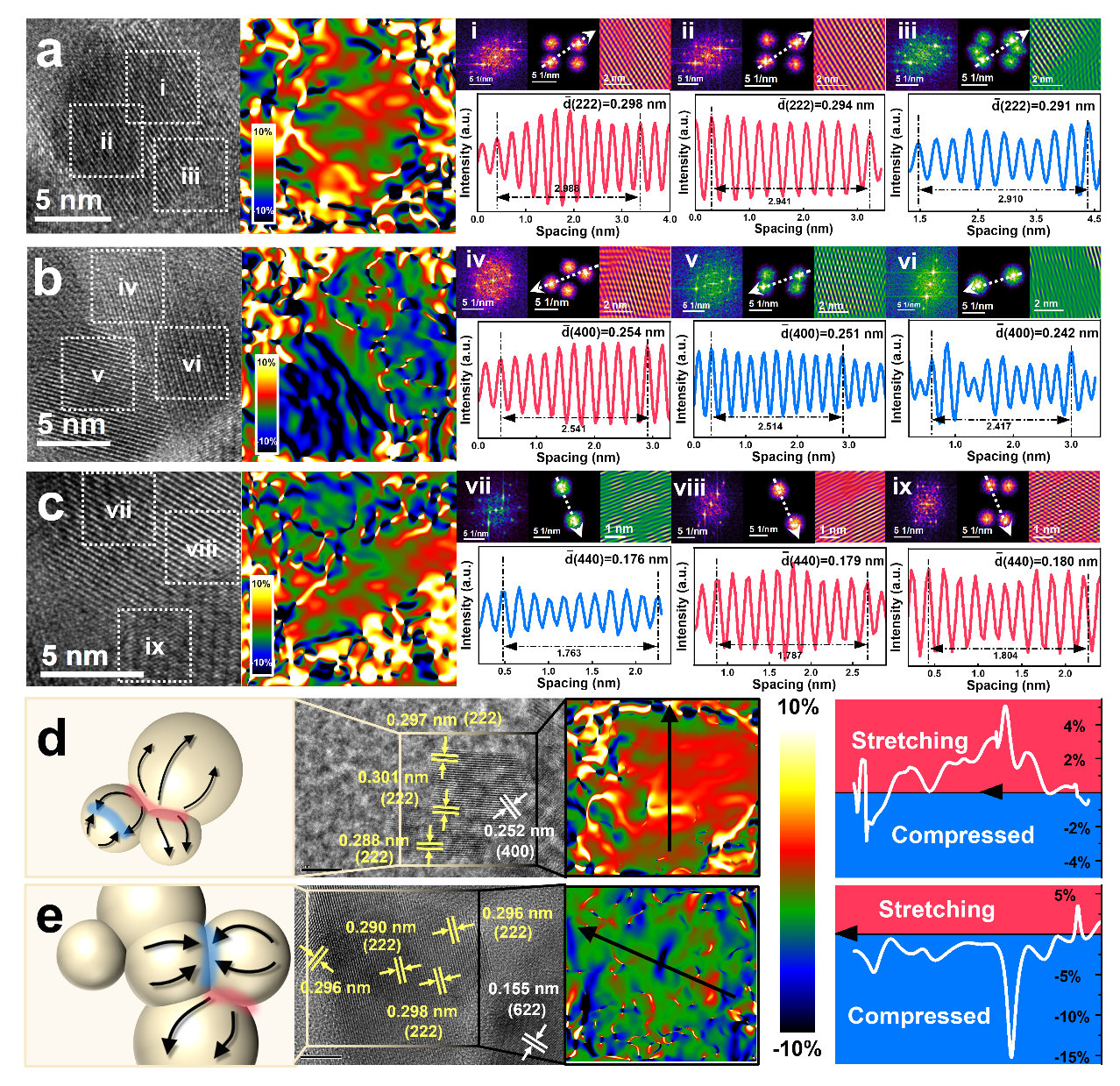


**Supplementary Figure 5: GPA analysis for InNT. a-c**, geometric phase analysis (GPA) of InNT, the section was introduced in Fig. S4. The following insert figures were the FFT, reverse FFT, and colorized images of different sections (i-iii were analyzed to be (222) facet, iv-vi were (400) facet and vii-ix were (440) facet). **d**, a section where stretching strain took the dominating state. **e**, a section of compressed strain took the dominating state.


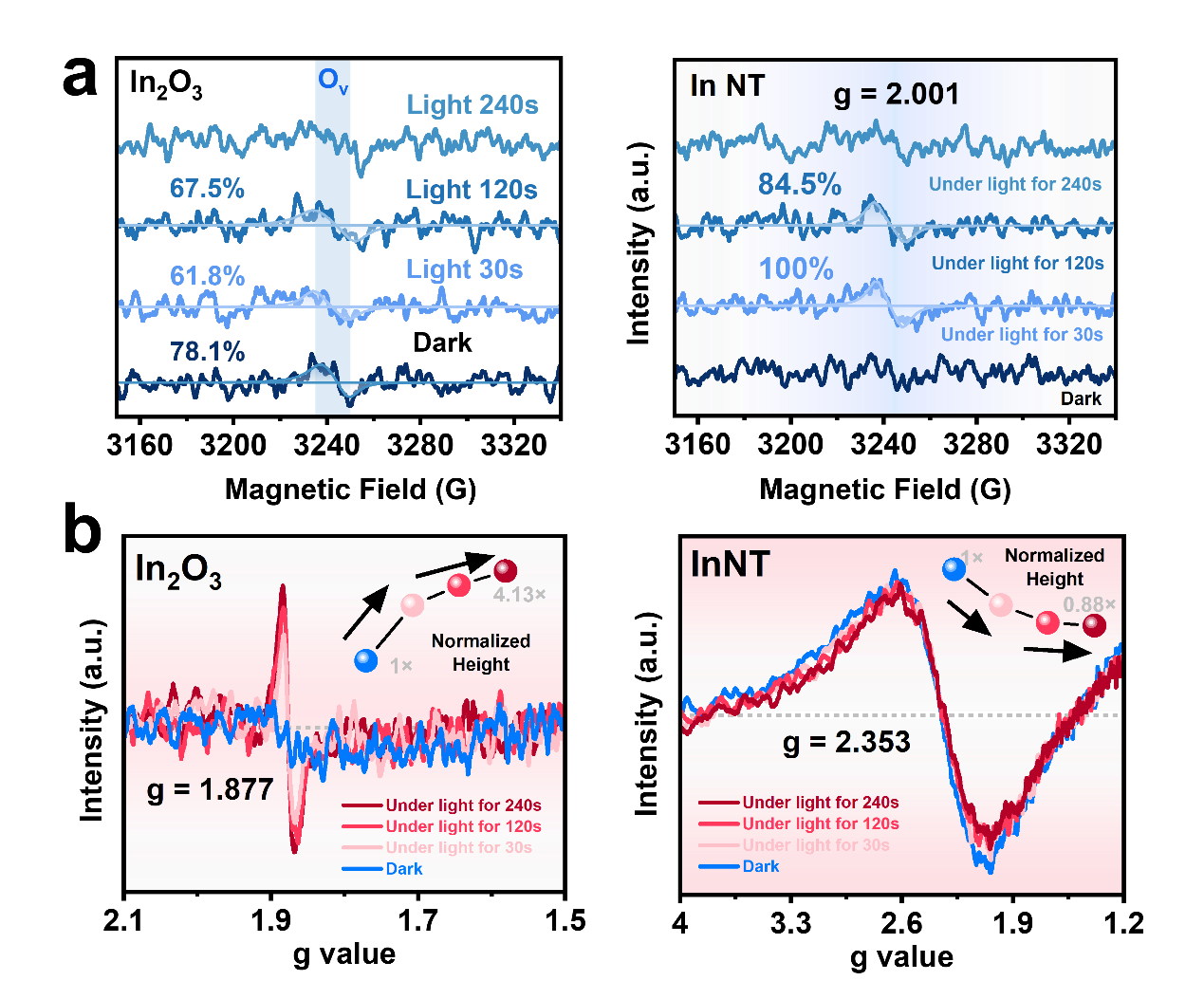


**Supplementary Figure 6: In situ ESR spectrum of In_2_O_3_ and InNT. a**, narrow spectrum, the mentioned number was obtained by normalization with the signal intensity of InNT-30 second. **b**, wide spectrum.

First, in situ narrow ESR spectrum was collected to analyze the Oxygen defects (g = 2.001) over In_2_O_3_ and InNT^15-17^. It was observed that the oxygen vacancy signal was not prominent in either sample. However, the signal in InNT became more pronounced as illuminated. This suggested that InNT could facilitate the formation of energy levels near oxygen vacancy, ergo, reducing the band gap energy, attracting electrons, inhibiting electron-hole recombination, and enhancing active metals' loading efficiency.

In the wide spectrum, it is observed that as the illumination time increased, In_2_O_3_ exhibited an increasing signal at g = 1.877 which corresponded to the electron transfer from the valence band (VB) to the conduction band (CB)^18^. Specifically, after light irradiation for 240 seconds, this signal was 5.23 times higher than observed in darkness. This signal continues to increase without showing any signs of plateauing, indicating that the charge transfer process from VB to CB is relatively slow. As to the InNT sample, a significant envelope peak is observed at g = 2.353. This phenomenon, similar to Fe_3_O_4_, may be attributed to the ferromagnetic coupling between In^3+^ and In^2+^, which suggested the potential interaction among numerous In atoms and defective sites which evidenced the presence of atom defects (both In and O defects) in InNT, leading to alterations in the paramagnetic properties of the sample. It is noteworthy that this signal at g = 2.353 diminished as irradiation time prolonged, suggesting that In^3+^ might act as an electron acceptor, resulting in the reduction of its valence state, leading to the formation of a new EPR-silenced In^x+^ species.


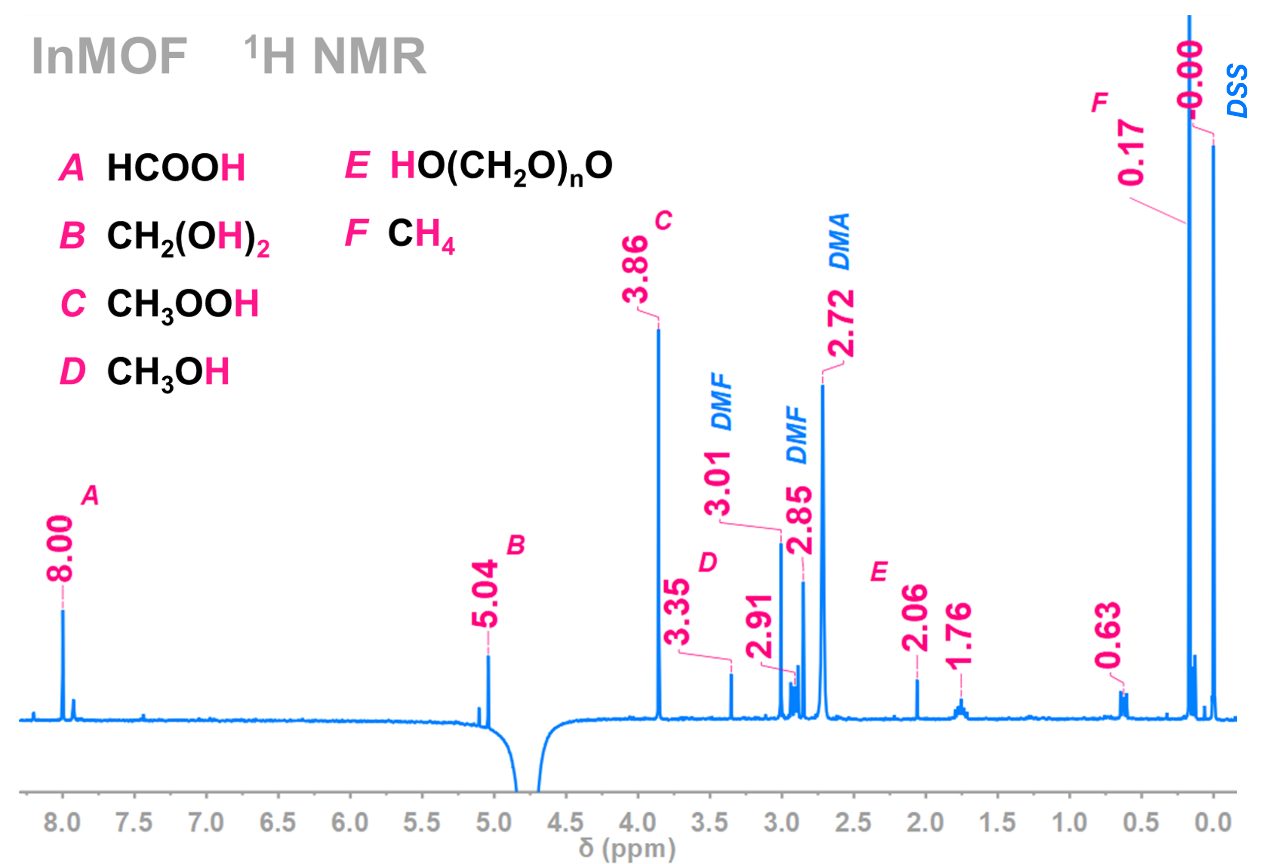


**Supplementary Figure 7: liquid phase products analysis of InMOF.**

Liquid phase products were measured by ^1^H NMR. 4,4-dimethyl-4-silapentane-1-sulfonic acid (DSS) was used as a correction for δ = 0.00. The detected products were formic acid (HCOOH, δ = 8.00), methylene glycol (CH_2_(OH)_2_, δ = 5.04), hydroperoxyl methane (CH_3_OOH, δ = 3.86), methanol (CH_3_OH, δ = 3.35), and paraformaldehyde (POM or HO(CH_2_O)_n_O, δ = 2.06)^19, 20^. Dissolved CH_4_ was also detected at δ = 0.17. In addition, there were signals at δ = 3.01 and 2.85 from the remaining DMF and a signal at δ = 2.72 for dimethylamine (DMA) from DMF decomposition.

The POM refers to polyformaldehyde with n<8. Since formaldehyde (H_2_CO) and water molecules (H_2_O) are in a chemical equilibrium state in an aqueous solution, and POM productivity was generally low, when calculating the C equilibrium here, the n in POM, that is, the number of C, was calculated as 1.


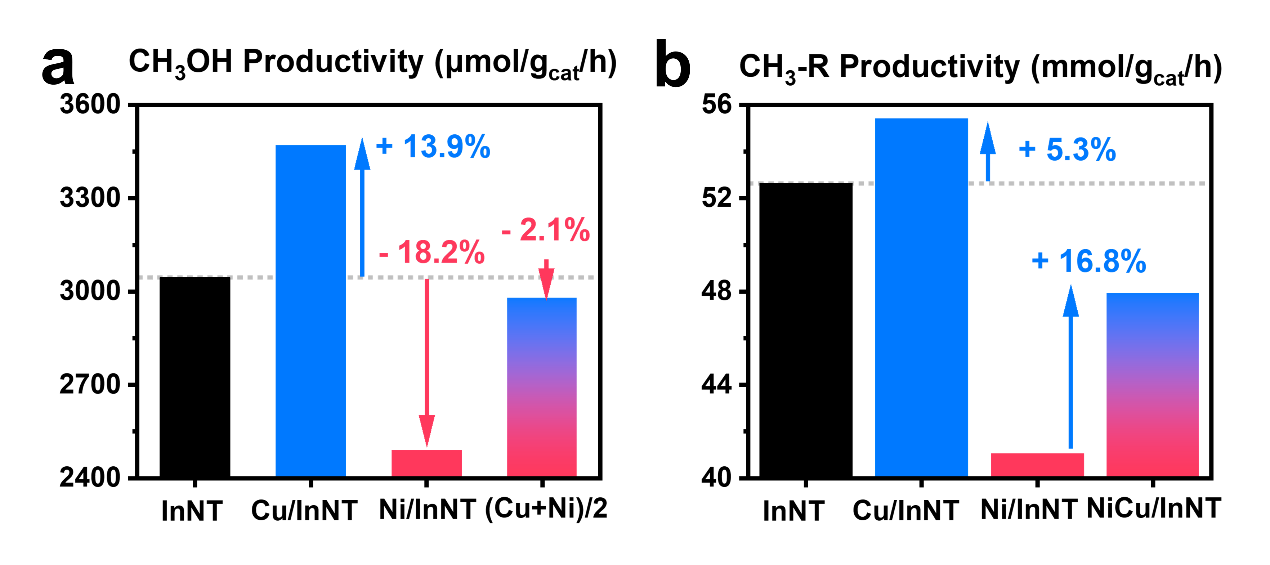


**Supplementary Figure 8: Overall comparison of different catalysts.**

(Cu+Ni)/2 stands for the average of Cu/InNT and Ni/InNT.

In single-metal samples, the impacts of Cu and Ni were measurably distinct, specifically, when only Cu was introduced, it boosted CH_4_ conversion reactivity (up to 55.42 mmol/g_cat_/h). Whereas when Ni was presented alone, an opposite trend was presented with the CH_3_-R yield being only 41.06 mmol/g_cat_/h, which was even lower than the pure InNT support. That is to say, the promotion effect of Cu itself is still insufficient for CH_3_OH production and the exclusive loading of Ni could even be inhibitive. Particularly, the combined average CH_3_OH yield of Ni/InNT and Cu/InNT was nearly identical to InNT, which raised the rational concern of when co-loading Cu and Ni will their effects compensate for each other.


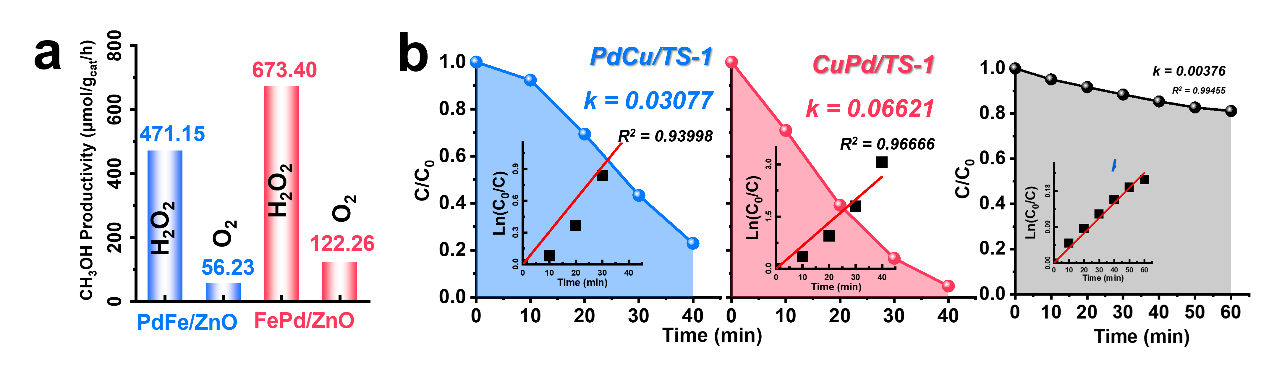


**Supplementary Figure 9: Altering loading order for different reactions. a**, Pd-Fe loaded on ZnO for methane conversion to methanol. **b**, Pd-Cu anchored over TS-1 for Methylene Blue adsorption.

PdFe/ZnO and FePd/ZnO were prepared by direct calcination of ZnO support and metal salts, about 0.25wt% of PdCl_2_ and FeCl_2_ was used. The reaction condition was room temperature, 30 bar CH_4_ (or 25 bar CH_4_ + 5 bar O_2_ without H_2_O_2_), 1 mL H_2_O_2_, 20 mg catalysts, 1000 rpm. It was observed that FePd/ZnO presented the highest catalytic activity for room temperature methane conversion, reaching a methanol yield of 673.40 μmol/g_cat_/h.

The preparation of PdCu/TS-1 and CuPd/TS-1 was similar to CuNi/InNT, about 0.25wt% of PdCl_2_ and CuCl_2_ was used. The reaction condition was room temperature with cycling cooling water, 500 W Xe lamp irradiation, Ar as protective gas, Methylene blue (MB) concentration: 20 ppm, 20 mg catalysts, 1000 rpm. The MB concentration was measured using a UV-vis spectrophotometer at 664 nm. Both PdCu/TS-1 and CuPd/TS-1 for MB adsorption followed first-order reaction kinetics. And CuPd/TS-1 gave the best adsorption rate and reaction kinetics constant.

This strategy of constructing different active sites by adjusting the loading sequence is generally feasible, and analysis was conducted on the combination of three elements (Cu-Ni-In, Fe-Pd-Zn and Cu-Pd-Ti). It was found that the ion radius and ionization energy actually have great impact on the loading process. In the process of photo deposition, it is better to load the larger ion first, and this sequence effect also plays a role in the direct calcination method.


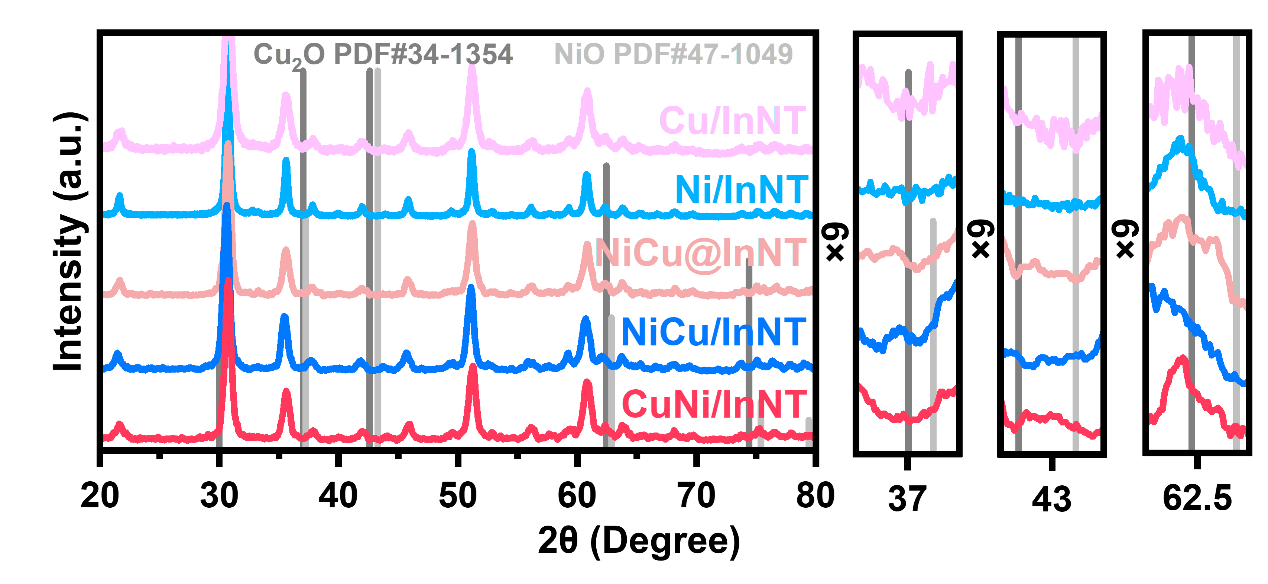


**Supplementary Figure 10: The XRD patterns of metal catalysts.**

All XRD patterns show the typical peaks assigned to the In_2_O_3_. No peaks assigned to Cu_2_O or NiO were observed, probably owing to their high dispersion as small nanoparticles.


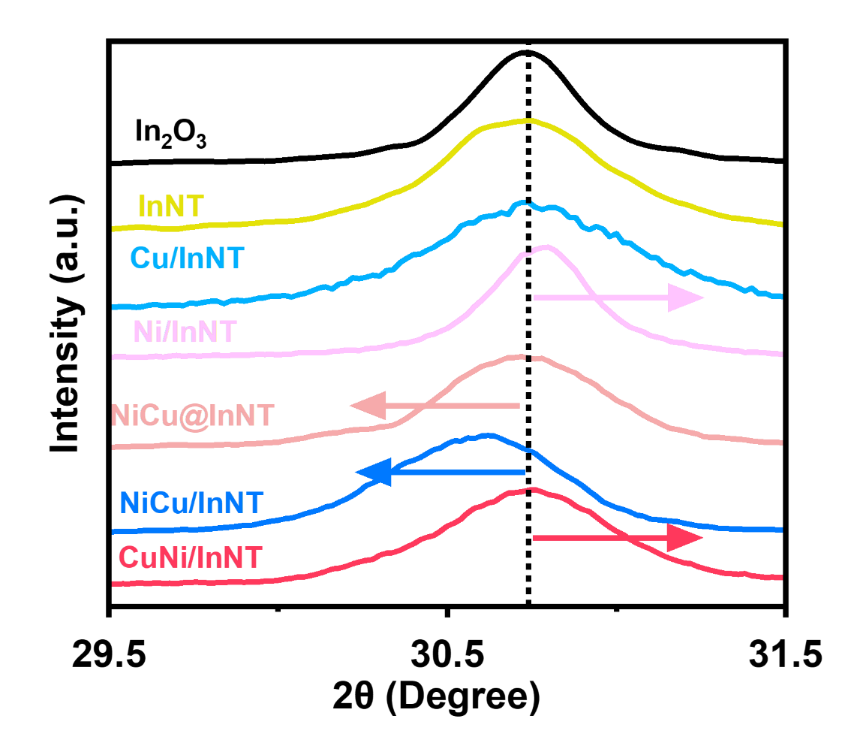


**Supplementary Figure 11: The In_2_O_3_ (222) peak deviation of catalysts.**

The bimetallic samples NiCu@InNT, NiCu/InNT was stretched, while CuNi/InNT was compressed, consistent with the XRD strain simulation results. However, Ni/InNT also exhibited compressed strain, which may be attributed to the presence of NiO clusters, as detected by FT-IR spectroscopy.

The Cu-O bond being longer than the In-O bond, leading to compression of the surrounding In-O space as Cu-O exceeds the cavity of the In_2_O_3_ framework, specifically the InO_6_ octahedra. Conversely, the Ni-O bond was shorter than the In-O bond, necessitating some degree of stretching to stabilize the Ni species thus Ni species might be extruded to the outer InNT surface. The strain effect also induced a notable change in the lattice parameter, as evidenced by the peak shift of In_2_O_3_ (222).


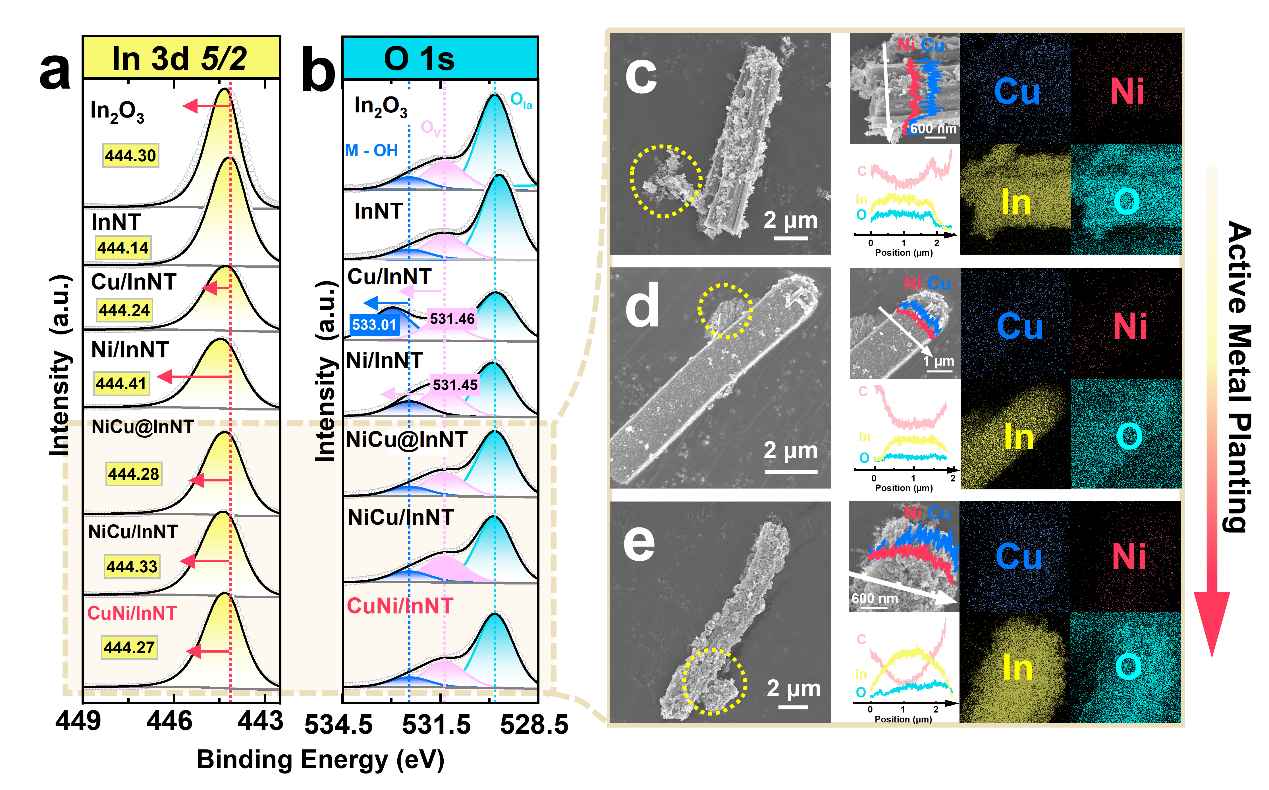


**Supplementary Figure 12: Active metals loading evidence of In catalysts. a**, In 3d 5/2 spectrum. **b**, O 1s spectrum. **c-e**, SEM images, line scanning and EDS mapping results of NiCu@InNT, NiCu/InNT and CuNi/InNT, respectively.

Compared to commercial In_2_O_3_, the binding energy of InNT shifted towards a lower direction, which was attributed to the generation of lattice mismatches during the calcination process, generating In vacancies and under-coordinated In^x+^ species at certain sites. Concurrently, due to the presence of carbon in the InMOF ligand, residual carbon after calcination would present an electron donation effect that also led to the lowering of the binding energy of In in InNT^21^. For monometallic samples, taking InNT as a reference, In acted as the electron donor, pushing electrons towards Cu and Ni, with Ni being more electronegative, causing a greater shift of In binding energy towards higher values^22^. Given the lower electronegativity of In, the trend of electron transfer from In to Cu and Ni should be consistent under similar coordination conditions. However, when compared with In_2_O_3_, Cu caused a decrease in binding energy, while Ni caused an increase, indicating that the active metal loading sites might be different, which was consistent with UV-DRS and subsequent DFT calculation results. For bimetallic samples, they all showed neutralized opposing trends of Cu and Ni, and compared to InNT, the binding energy of In in all samples increased, indicating that the trend of In pushing electrons towards active metals remains unchanged. while the different peak values still suggested that there were indeed configurational differences. Notably, the In peak of the NiCu sample shifted the most obverse compared to InNT, suggesting a significant impact on the structure of InNT which might suggest the highest surface nanoparticle density. Analysis of the O 2p revealed that, in comparison to In_2_O_3_, the O in InNT exhibited a lower shift which also indicated the presence of In vacancies and under-coordinated In^x+^ at certain sites. Given that oxygen has a higher electronegativity than carbon, it is not plausible for carbon to donate electrons to oxygen, confirming the existence of In defects. Upon analysis of the monometallic samples, a conclusion was drawn that aligns with the description for the In monometallic sample, indicating that the loading sites for the two metals were distinct. For the NiCu sample, once Ni was loaded the first, a certain degree of nucleation would occur, and subsequent addition of Cu would grow near the NiO clusters, resulting to the decrease in the binding energy of both indium and oxygen.

SEM images showed that the morphology of the nanotubes was maintained after loading metals. The line scanning and EDS mapping showed the presence of a small amount of N with some C attached to the outside of the nanotubes, and Cu and Ni species were evenly distributed without any obvious agglomeration.


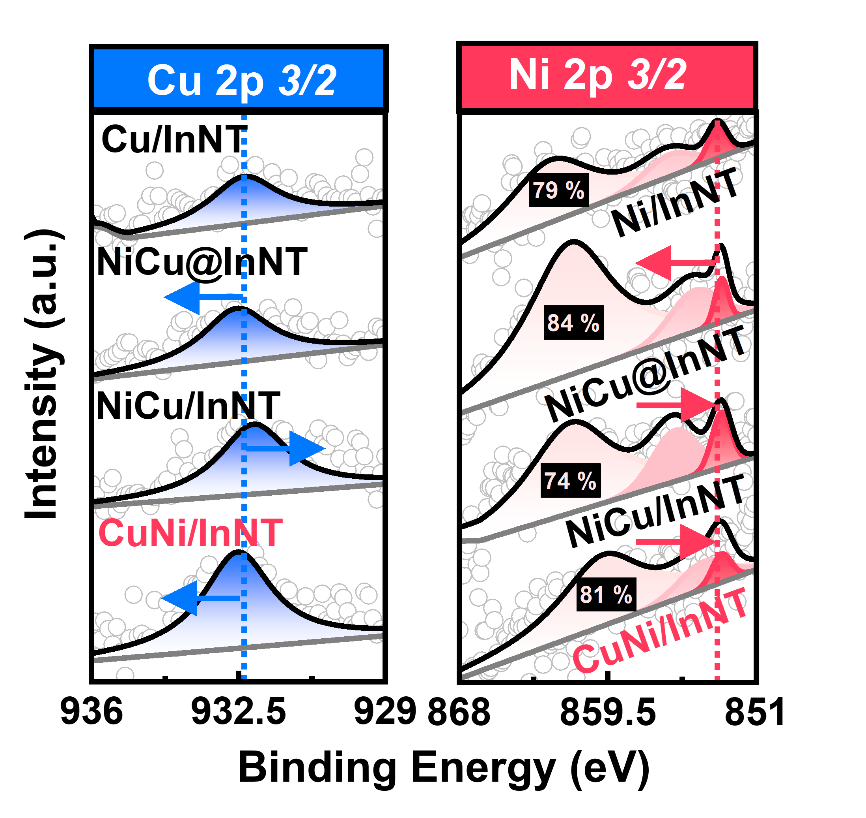


**Supplementary Figure 13: Cu and Ni 2p 3/2 spectrum of InNT catalysts**.

The Cu and Ni 2p 3/2 spectrums revealed that they were in Cu_2_O and NiO states in all samples. And apart from the monometallic counterpart, their peak appeared obvious shifts in the three bimetallic samples, further suggesting that the coordination environments of the three bimetallic systems were distinct.


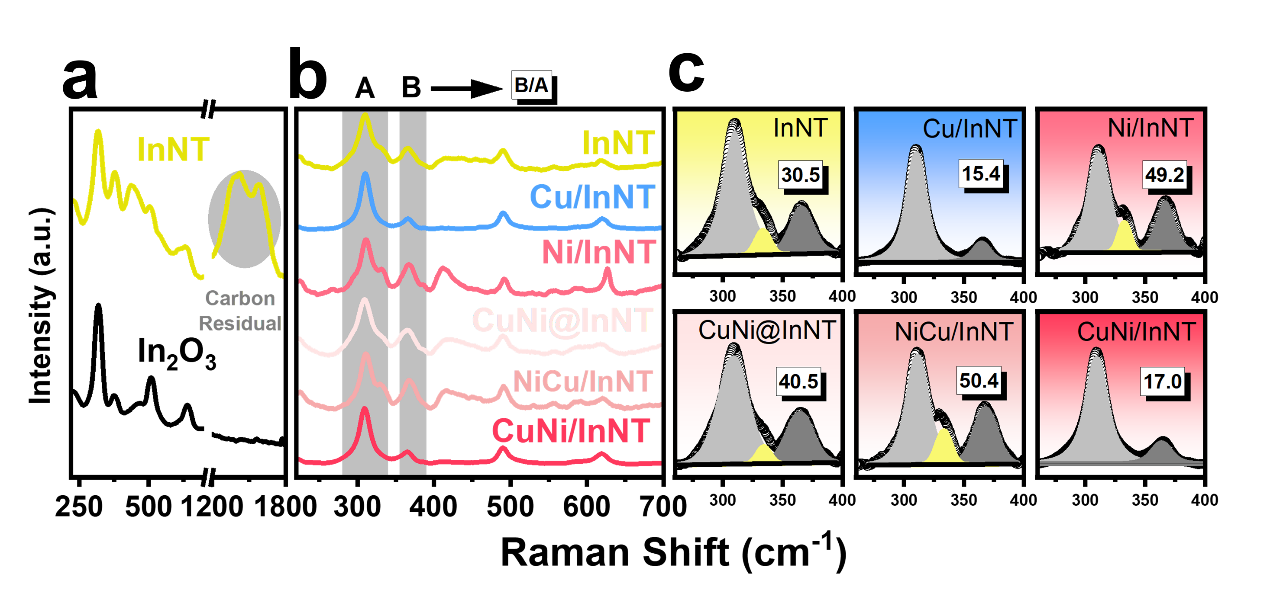


**Supplementary Figure 14: Raman data of InNT catalysts**. **a**, the wide range results of In_2_O_3_ and InNT. **b**, the Raman data in the range of 180-700 cm^-1^ of InNT catalysts, peak A referred to InO_6_, peak B referred to In-O-In. **c**, the peak deconvolution results of InNT catalysts.

Some residual carbon could be seen in the InNT sample. The InO_6_ octahedron was compressed at 305.9 cm⁻¹, the band at 363.8 cm⁻¹ corresponded to the In-O-In bending mode, and the bands at 491.1 cm⁻¹ and 618.3 cm⁻¹ were attributed to the stretching vibrations of InO_6_^23^. The InO_6_ stretching peak at 618 cm⁻¹ in the single Ni sample showed a significant shift compared to other samples. Combined with the XRD simulation results, this could be attributed to the presence of NiO causing lattice expansion in InNT.

No peaks corresponding to Cu-O or Cu-Cu were observed, indicating that copper was highly dispersed in all samples without any nucleation^24^. An increase in -OH groups was previously noted in the Cu/InNT sample, but no peaks assignable to In(OH)_3_ or InOOH were detected, suggesting that the indium oxide support itself remains unaltered. Bands around 411.5 cm⁻¹ and 559.3 cm⁻¹ could be attributed to NiO-related peaks, indicating the presence of NiO, especially in the Ni/InNT sample^17, 25, 26^.

Raman deconvolution fitting (area ratio of 363.8 cm⁻¹/305.9 cm⁻¹) was also used to elucidate the extent of defects as evidenced by the area ratio of B/A. Compared to the XPS results, Raman spectroscopy has a deeper analysis depth, providing data from both the surface and bulk simultaneously^27^.


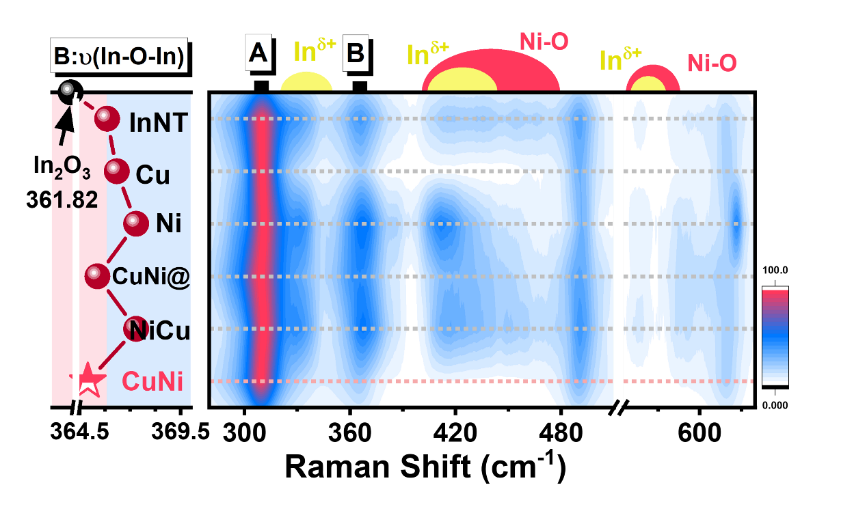


**Supplementary Figure 15: Raman proof for the existence of In^x+^.**

The In-O-In signal (363.8 cm⁻¹) shifted towards higher wavenumbers indicated a decrease in bond length and an increase in bond energy. Comparing the In-O-In bond of all metal-loaded catalysts with that of InNT, it was found that only the peaks for NiCu@InNT and CuNi/InNT shifted in the direction of decreasing bond energy and increasing bond length. This was inconsistent with the results of the GPA analysis and XRD simulations, suggesting that the peak shift was more likely related to the formation of a specific configuration, leading to changes in the In-O-In.

The peaks at 331.3, 411.5, and 559.3 cm⁻¹ correspond to In^x+^ with oxidation states lower than +3. Compared to In_2_O_3_, the emergence of these peaks on InNT indicated the presence of In with lower coordination situations and the existence of both O and In defect sites (note that the NiO peaks overlapped with In^x+^, but there was no overlap at 321.4 cm⁻¹, allowing for differentiation). These In^x+^ peaks did not appear on Cu/InNT and CuNi/InNT catalysts as Cu was firstly loaded on them, suggesting that Cu may fill the defect sites, making their crystalline structure more similar to that of complete In_2_O_3_. In contrast, all samples loaded with Ni first exhibited more prominently In^x+^ signals, indicating that the presence of Ni further intensified the defect situation in InNT.


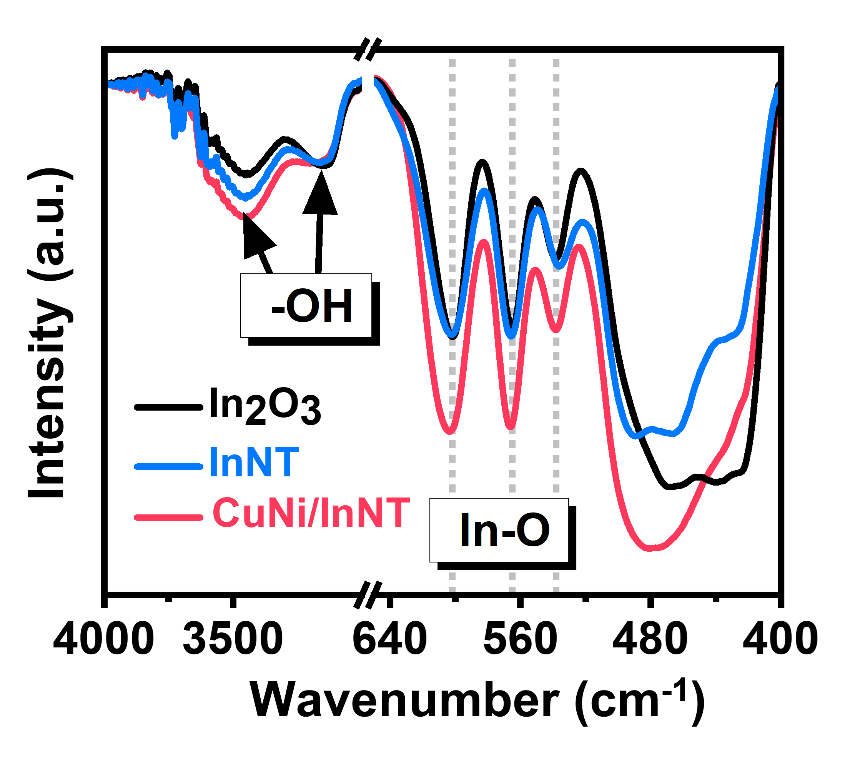


**Supplementary Figure 16: FT-IR spectrum of In_2_O_3_ InNT and CuNi/InNT.**

Metal vacancies are more sensitive to the presence of H_2_O, hence easier to depict -OH signals. Compared with In_2_O_3_, the -OH signal of InNT was stronger, indicating the presence of In vacancies in InNT^26^.

Cu(I)-O was located at 607 cm⁻¹. In the measured signals, the peak originally presented at 602.1 cm⁻¹ in InNT was shifted to 603.6 cm⁻¹ in CuNi/InNT, which can be attributed to the influence of Cu(I)-O^24^. Furthermore, the signals corresponding to Ni-O (near 480 cm⁻¹ and 440 cm⁻¹) were markedly more pronounced in CuNi/InNT^28, 29^. Collectively, these observations confirmed the presence of Cu(I)-O and Ni-O in the CuNi/InNT sample.


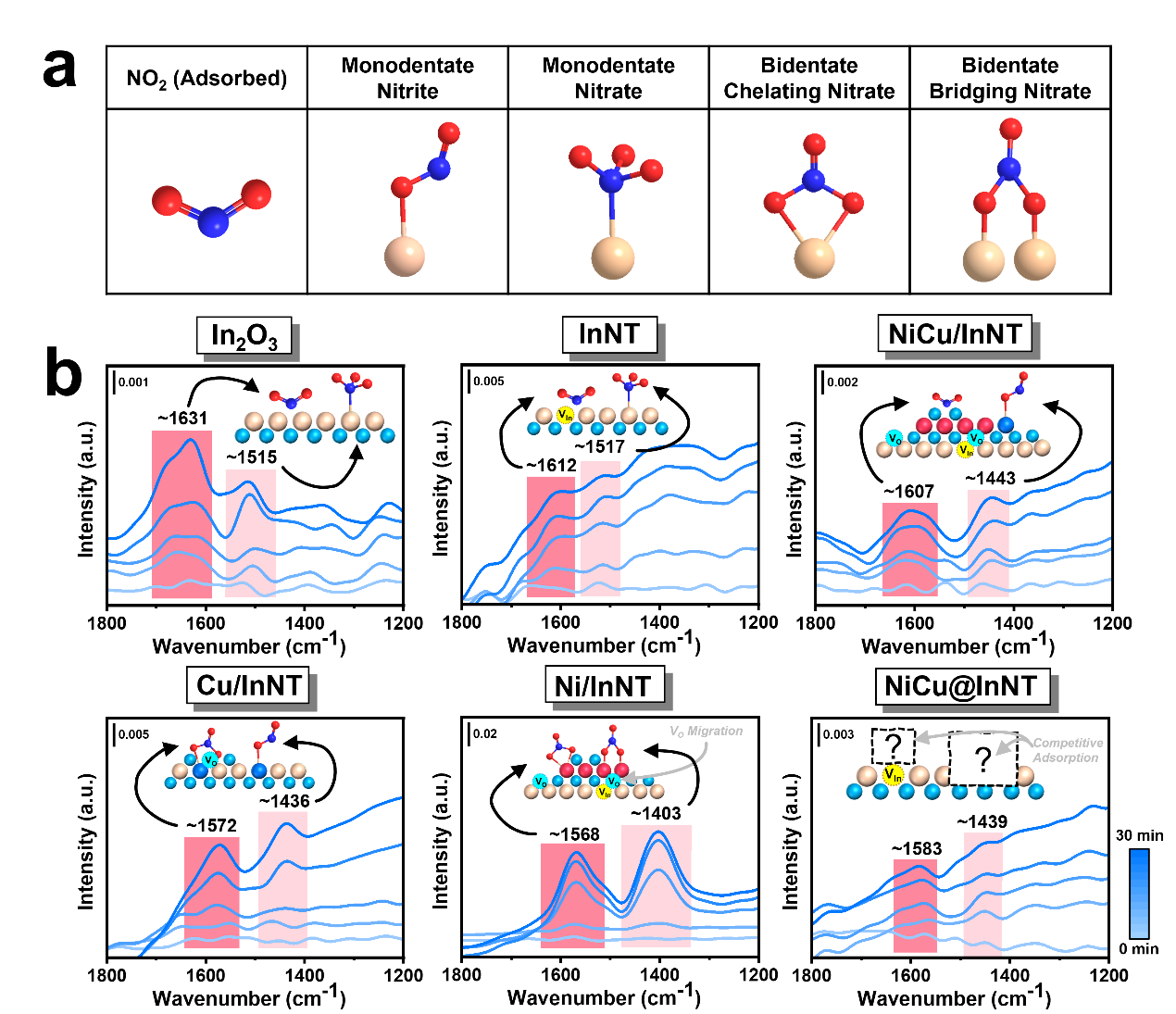


**Supplementary Figure 17: NO-DRIFTS results of catalysts**. **a**, schematic diagram of different NO adsorption configurations. **b**, NO-DRIFTS results of In_2_O_3_, InNT, Cu/InNT, Ni/InNT, NiCu/InNT and NiCu@InNT.

There were 5 main NO adsorption configurations (adsorbed NO_2_, monodentate nitrite, monodentate nitrate, bidentate chelating nitrate, and bridging nitrate)^30-33^.

The existence of adsorbed NO_2_ could only be seen over In_2_O_3_ and InNT, suggesting this signal was associated with NO surface adsorption over In-O. It was weakened over InNT also implied the presence of V_In_. Notably, it was also observed over NiCu/InNT sample with a slight decrease in wavenumber. This might be due to the adsorption of NO_2_ over NiO clusters. The monodentate nitrite mostly existed over Cu-based samples. The monodentate nitrate mostly existed over pure In samples. The bidentate chelating nitrate could appear over both Cu and Ni-based samples. But the wavenumbers upon two dominating active metals were different which was associated with higher bonding strength over Ni and relatively lower bonding strength over Cu. The bridging nitrate’s appearance suggested the existence of nano clusters. Consistent with other characterization results, it was only observed on Ni/InNT, proving the formation of NiO. At the same time, the deviation of this position from that reported in the literature indicates that the NiO cluster may not be a perfect crystal, which also indicates the existence of oxygen migration.

It is also worth noting that NiCu@InNT’ signal type is the same as CuNi/InNT, but the intensity was slightly lower which was in agreement with XPS and EDS mapping results, evidencing that simultaneous addition of Cu and Ni could form similar special active sites with CuNi/InNT. But Cu and Ni would undergo competitive adsorption, causing insufficient loading of both metals.


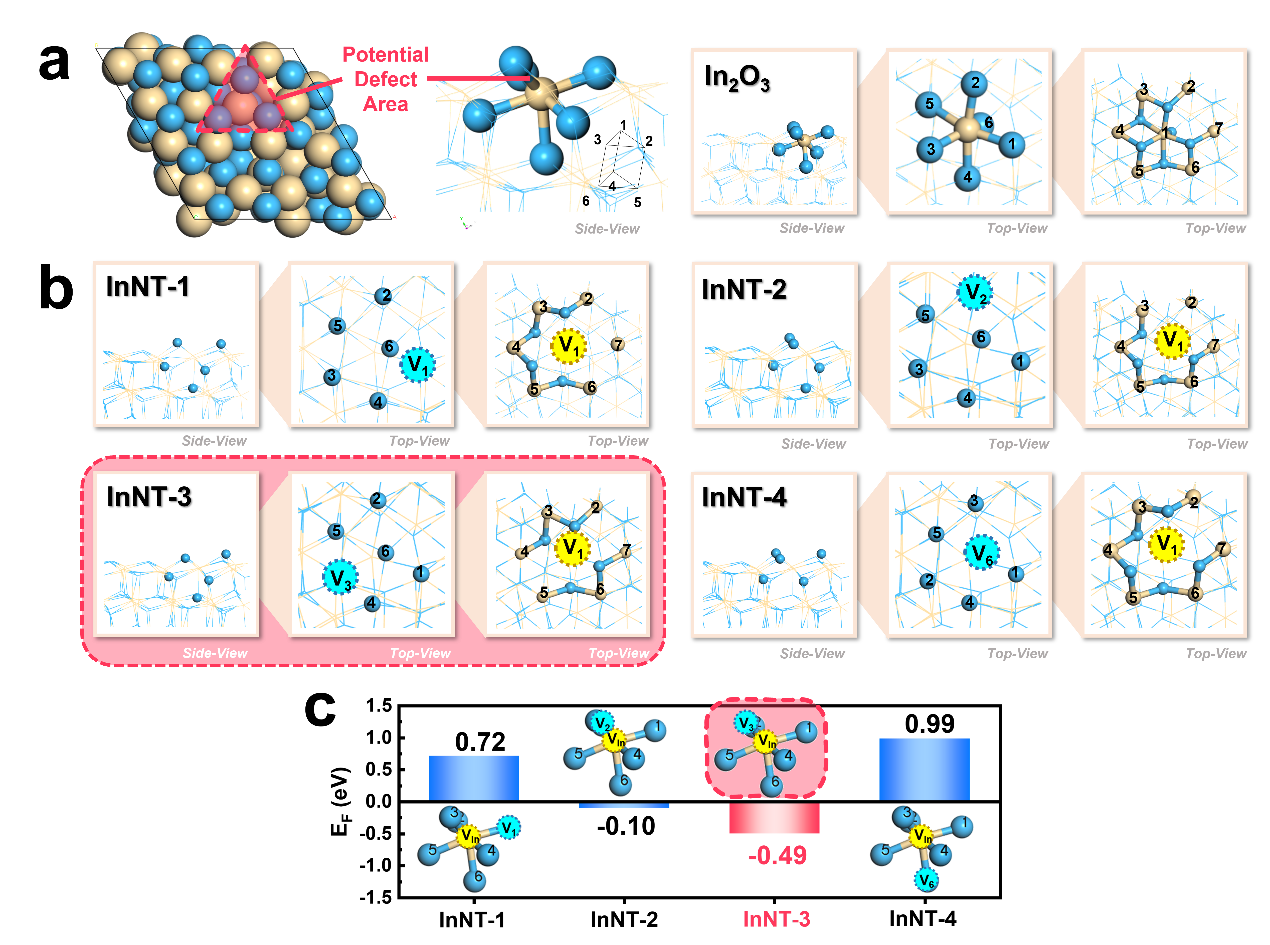


**Supplementary Figure 18: Theoretical structure diagram of In_2_O_3_ and InNT**. **a**, schematic diagram In_2_O_3_. **b**, several possible configurations of InNT. **c**, calculated formation energy of InNT-1 to InNT-4.

Incorporating previous characterizations such as UV-DRS and XPS, the defect scenarios within a standard unit (In_1_O_6_) were designed. With the confirmation of In defects were present over InNT, there was primarily two types of oxygen vacancies: three surface-layer O vacancies and three sub-layer O vacancies. Calculations of formation energies revealed that InNT-3, with a formation energy as low as -0.49 eV, wase most stable configuration. Subsequent calculations have opted for InNT-3 as the model for InNT, serving as the basis for subsequent configurations involving the active metals anchoring.

Particularly, it was found that the formation energy of defects at the surface 1-3 sites is lower than that of the sub-layer O_V_. This result was consistent with the observation of a higher number of O_V_ in InNT from Raman spectroscopy, while XPS peak fitting indicated higher number of O_V_ in In_2_O_3_. This reiterated that the majority of O_V_ formed in InNT during the calcination process were in the bulk phase which would transfer to the surface during the photo-deposition process.


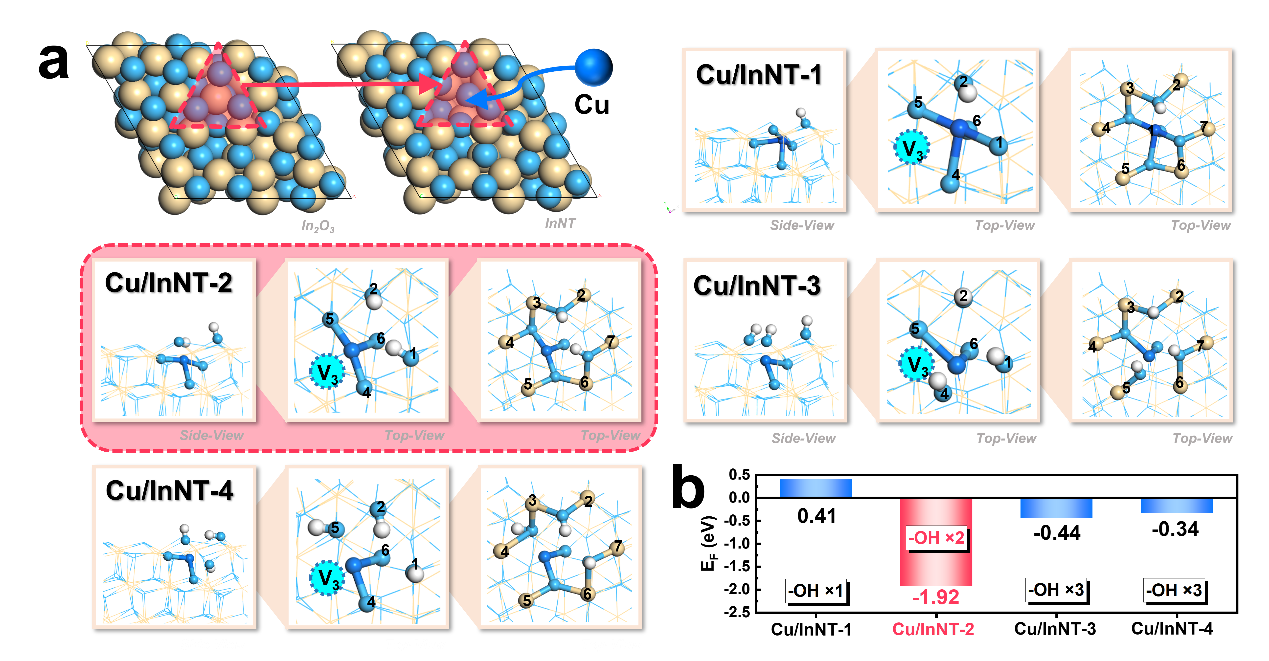


**Supplementary Figure 19: Theoretical structure diagram of Cu/InNT**. **a**, schematic diagram Cu/InNT and several possible configurations of InNT. **b**, calculated formation energy of Cu/InNT-1 to Cu/InNT-4.

In XPS characterization, it was observed that Cu/InNT contained an abundance of surface hydroxyl groups, which were likely to be present at coordinatively unsaturated oxygen sites. Integrating these phenomena, we proposed four possible configurations for the active copper sites and calculated their formation energies accordingly. Ultimately, it was determined that the configuration with two -OH groups has the lowest formation energy, reaching -1.92 eV, making it the most appropriate Cu/InNT configuration.


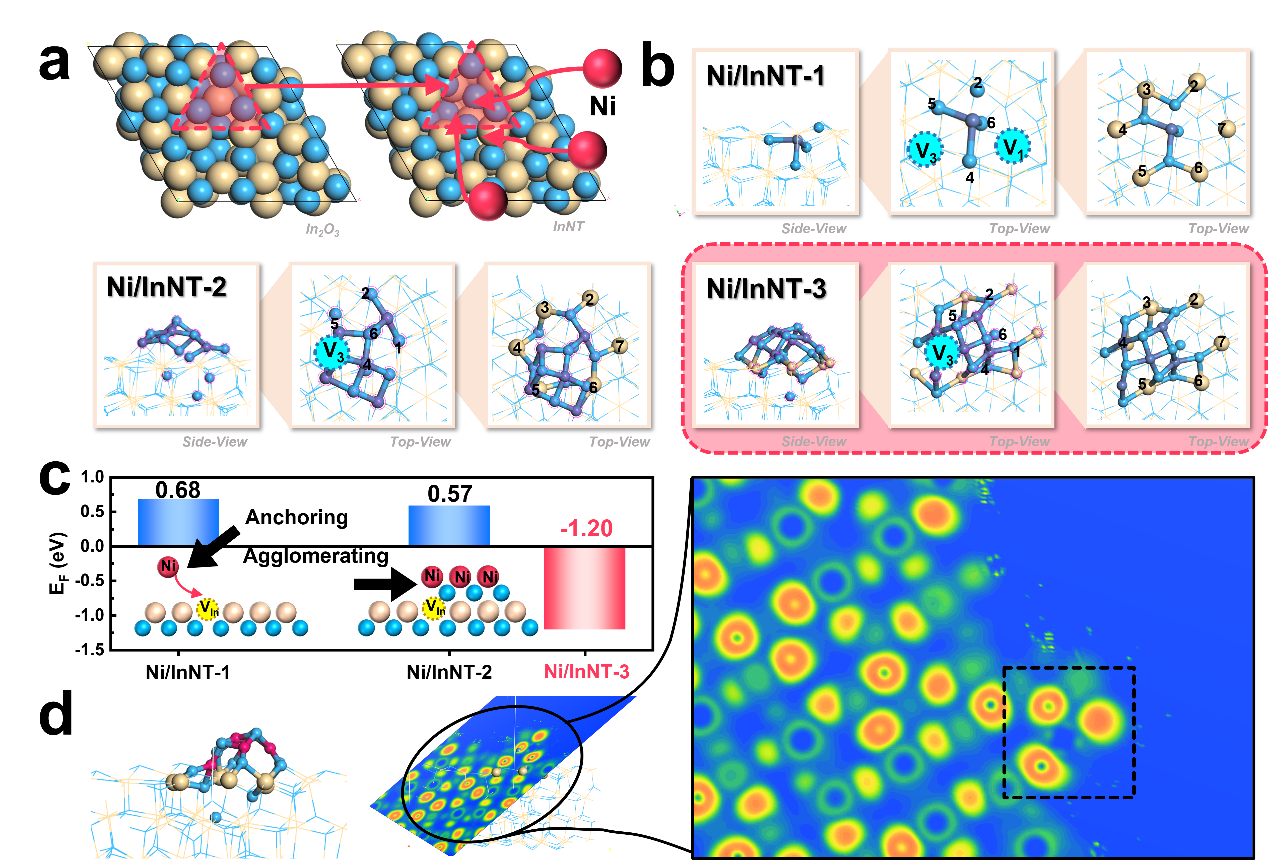


**Supplementary Figure 20: Theoretical structure diagram of Ni/InNT**. **a**, schematic diagram Ni/InNT. **b**, several possible configurations of InNT. **c**, calculated formation energy of Cu/InNT-1 to Cu/InNT-4. **d**, the calculated electron localization function results of Ni/InNT-3.

The formation energies of Ni embedded as single atoms into In vacancies (Ni/InNT-1), loaded as a 5-atom cluster (Ni_5_O_5_) near In vacancies (Ni/InNT-2), and loaded as an irregular 5-atom cluster with oxygen defects (Ni_5_O_4_) near In vacancies were calculated (Ni/InNT-3). It was found that the energy barrier was indeed lowest when existing in the form of imperfect crystalline clusters (Ni/InNT-3). Particularly, the energy barrier was significantly high when Ni grows in the form of single atoms, which is consistent with the characterization results indicating the presence of NiO clusters.


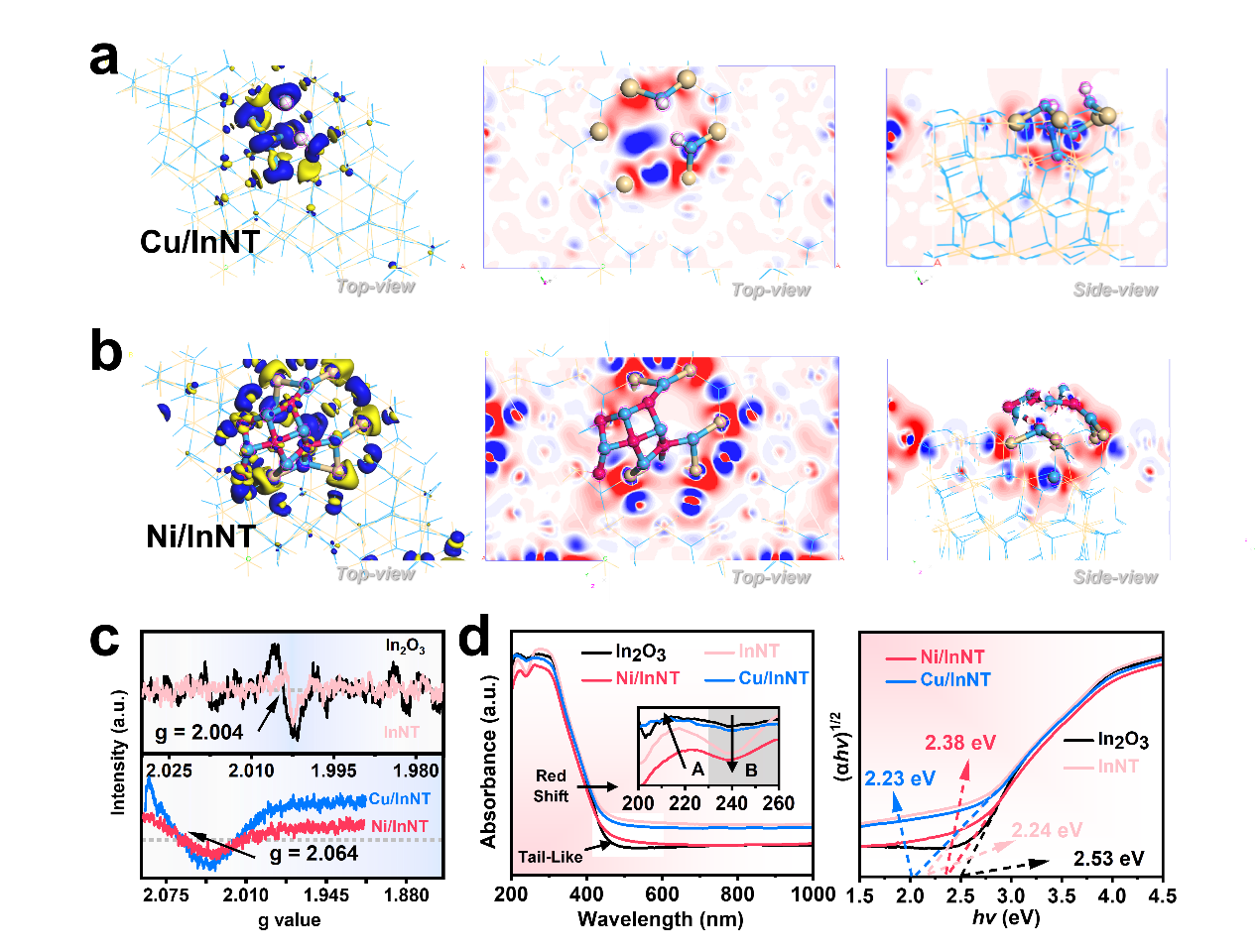


**Supplementary Figure 21: Charge density difference diagram, EPR, and UV-Vis-DRS differences of Cu/InNT and Ni/InNT**. In the first column of Fig. 21a-b, blue represents obtaining electrons, yellow represents donating electrons.

Upon comparing the photo-response EPR spectra of InNT and In_2_O_3_, it was revealed that InNT possessed a significant number of defects (V_In_ and V_O_) and disordered sites. The low-temperature EPR near g=2.00 confirmed the presence of V_O_ in varying concentrations in both materials, suggesting distinct coupling mechanisms between Cu and Ni species with these defect sites. A pronounced peak at g=2.064, attributed to In^2+^, was observed in the single-metal catalysts, with Cu/InNT exhibiting a higher intensity than Ni/InNT, suggesting that both metals would alter electron states of In, while Cu/InNT showed a more pronounced signal, possibly due to Cu could directly alter the local coordination of In. Therefore, Cu was potentially in an intercalated state in Cu/InNT.

UV-Vis-DRS characterization showed the bandgap of In_2_O_3_ was 2.53 eV, which decreased to 2.24 eV in InNT due to carbon residuals and lattice dislocations. Loading Cu further narrowed the band gap (2.23 eV), while loading Ni unexpectedly widened it (2.38 eV), suggesting that forming CuNi/InNT on a Cu/InNT base was more feasible than forming NiCu/InNT. The widened band gap upon Ni/InNT contrasted with the increase in oxygen vacancies (V_O_) observed by XPS because typically, more V_O_ would narrow the bandgap^34^. This discrepancy may arise from the formation of a heterojunction between p-type NiO (with a broad bandgap >3 eV) and n-type In_2_O_3_, leading to a potential charge barrier at the n-In_2_O_3_ and p-NiO interface, which affects the sample's bandgap^35, 36^.

The shape of the In_2_O_3_ tail differed from that of InNT, with InNT exhibiting a redshift, indicating the presence of metal defects. All samples showed a broad peak from 214 to 300 nm, attributed to the ligand-to-metal charge transfer (LMCT). The characteristic peak A of InNT shifted with the loading of active metals, showing a blue shift (InNT:213, Ni/InNT:217, Cu/InNT: 222), primarily due to the presence of M-OH, a trend consistent with the increase in -OH observed by XPS. Peak B attributed to LMCT from unsaturated coordination structures, was prominently observed in the Ni/InNT and InNT. This suggested that the deposition of Ni did not infiltrate the defects and remained on the surface, thus could not fulfill the unsaturated crystal structure of InNT. On contrast, Cu, with a larger ionic radius, was more likely to bond with oxygen near V_In_, integrating into defect sites and compensating for the charge density there, which results in a less pronounced peak.


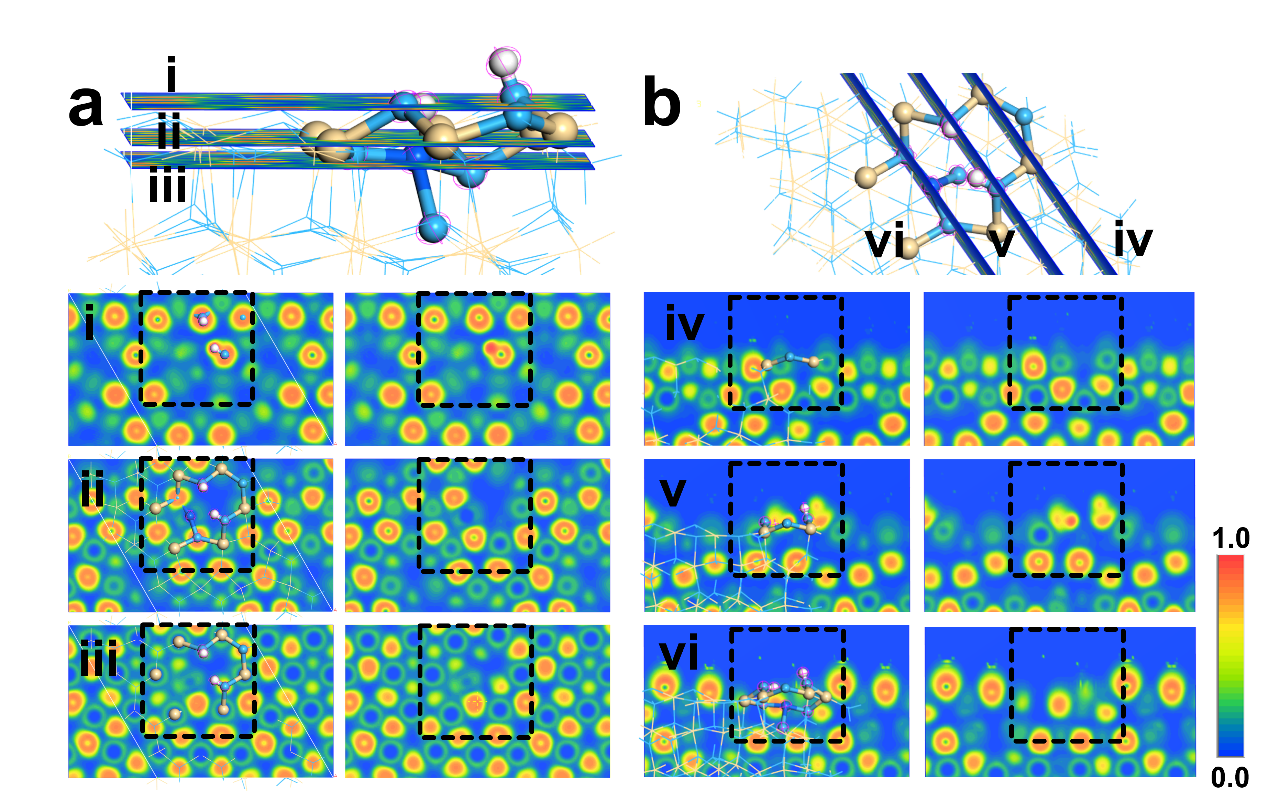


**Supplementary Figure 22: EIF results of Cu/InNT**.

Highly localized electrons typically indicate a greater degree of confinement or trapping of electrons within specific spatial regions. In other words, electrons within such regions tend to remain localized rather than moving freely throughout the entire space. Analysis was conducted on the Cu/InNT configuration by several ELF planes at various angles, revealing that the ELF function was elevated particularly near Cu and, more prominently, around the -OH groups. This suggested a higher electron density in these areas, thereby providing an electron-rich anchoring region for the subsequent adsorption of Ni^3+^ precursor salts.


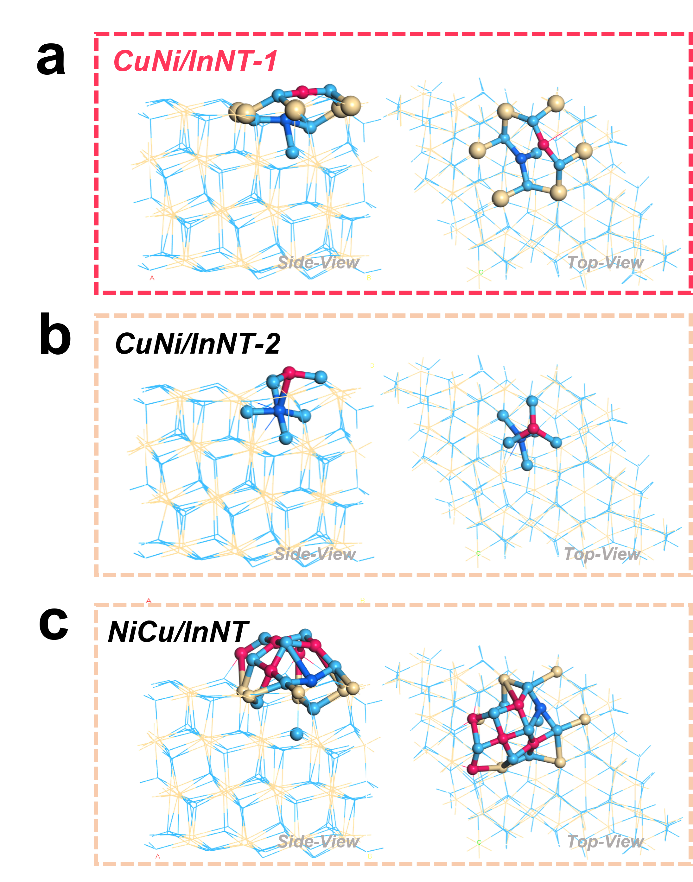


**Supplementary Figure 23: Possible theoretical structure diagram of CuNi/InNT and NiCu/InNT**. **a**, schematic diagram of CuNi/InNT with Cu-O-Ni bond. **b**, schematic diagram of CuNi/InNT with Cu-Ni bond. **c**, schematic diagram of NiCu/InNT.


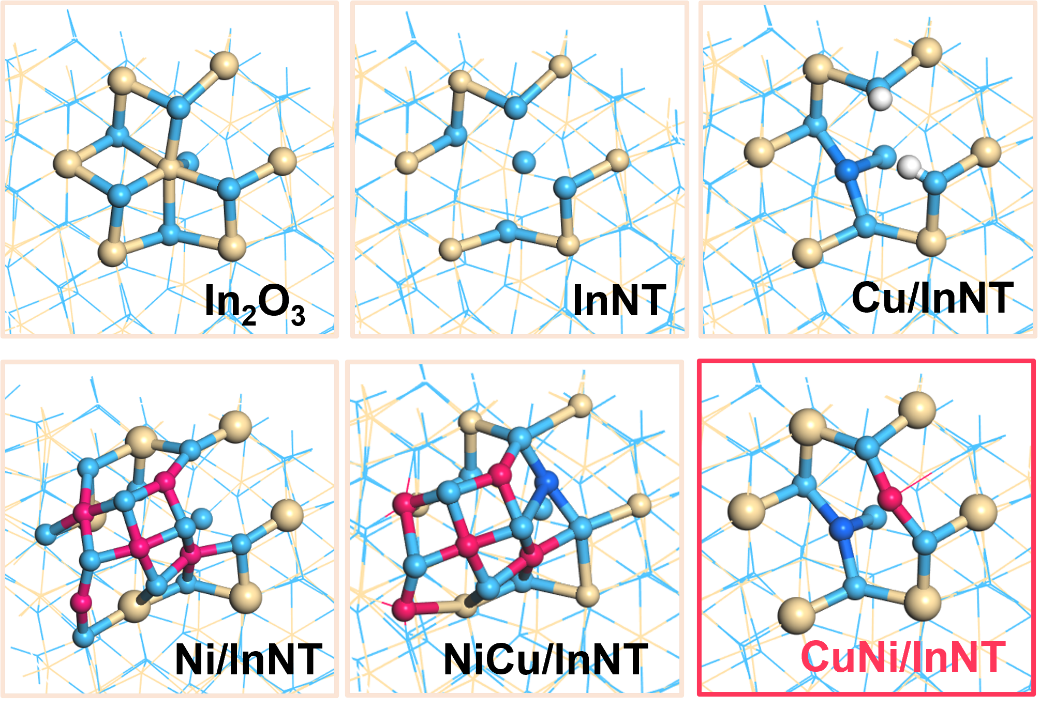


**Supplementary Figure 24: Theoretical structure diagram of InNT-based catalysts**.


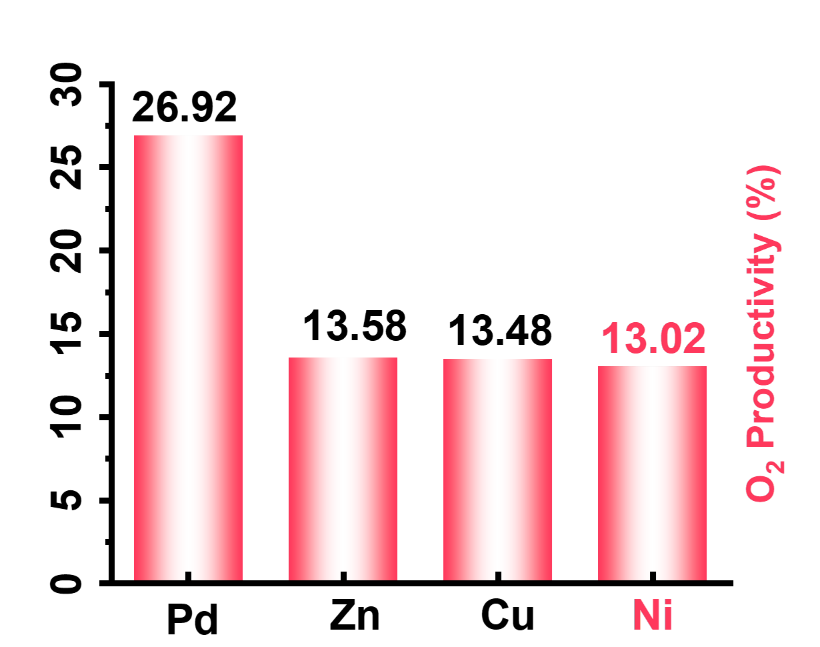


**Supplementary Figure 25: The O_2_ generation ability of CuPd/InNT, CuZn/InNT, and CuCu/InNT.**

Experiments using CO_2_ to replace CH_4_ was redesigned as the reaction conditions of CO_2_+H_2_O_2_ were similar to CH_4_+H_2_O_2_ only that the CO_2_ could not be further oxidized. Therefore, analyzing the decomposition of H_2_O_2_ under this situation could determine the specific mechanism of how the binary site spacing affecting the substrate activation. From the experimental results, except that Pd produced excessed O_2_ due to its strong H absorption ability, the generation tendency of O_2_ production was more than mediocre, but it is still consistent with the metal spacing (the larger the spacing, the more O_2_, which led to less ·OH radicals). However, the gentle change of O_2_ yield was far from the steep change of CH_3_OH yield. The huge change of overall reaction activity cannot be solitary explained by the effect of diatomic spacing on H_2_O_2_ decomposition. Therefore, the heteronuclear diatomic distance must affect the directional tensile polarization of the C-H bonds of CH_4_, and ultimately lead to the variations of reaction performance.


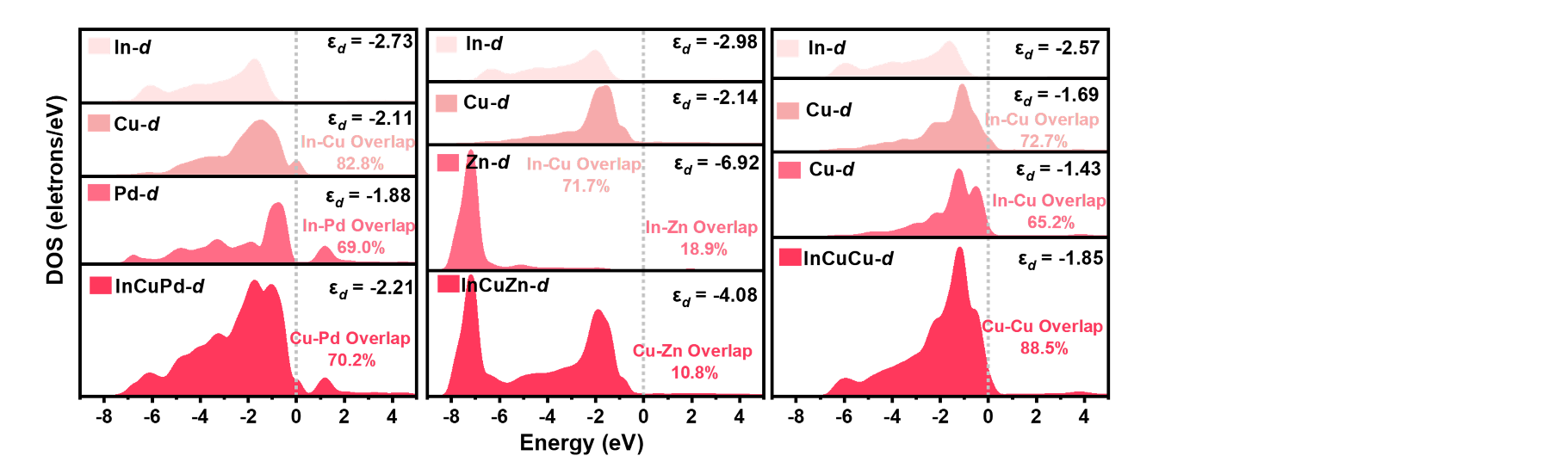


**Supplementary Figure 26: The d-orbital hybridization results of CuPd/InNT, CuZn/InNT, and CuCu/InNT.**


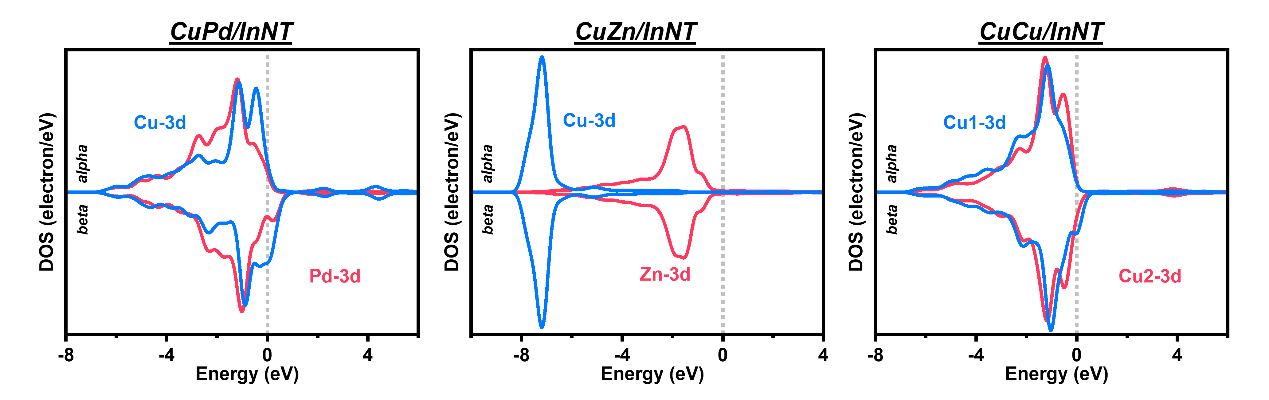


**Supplementary Figure 27: The 3d-orbital situations of CuPd/InNT, CuZn/InNT, and CuCu/InNT.**


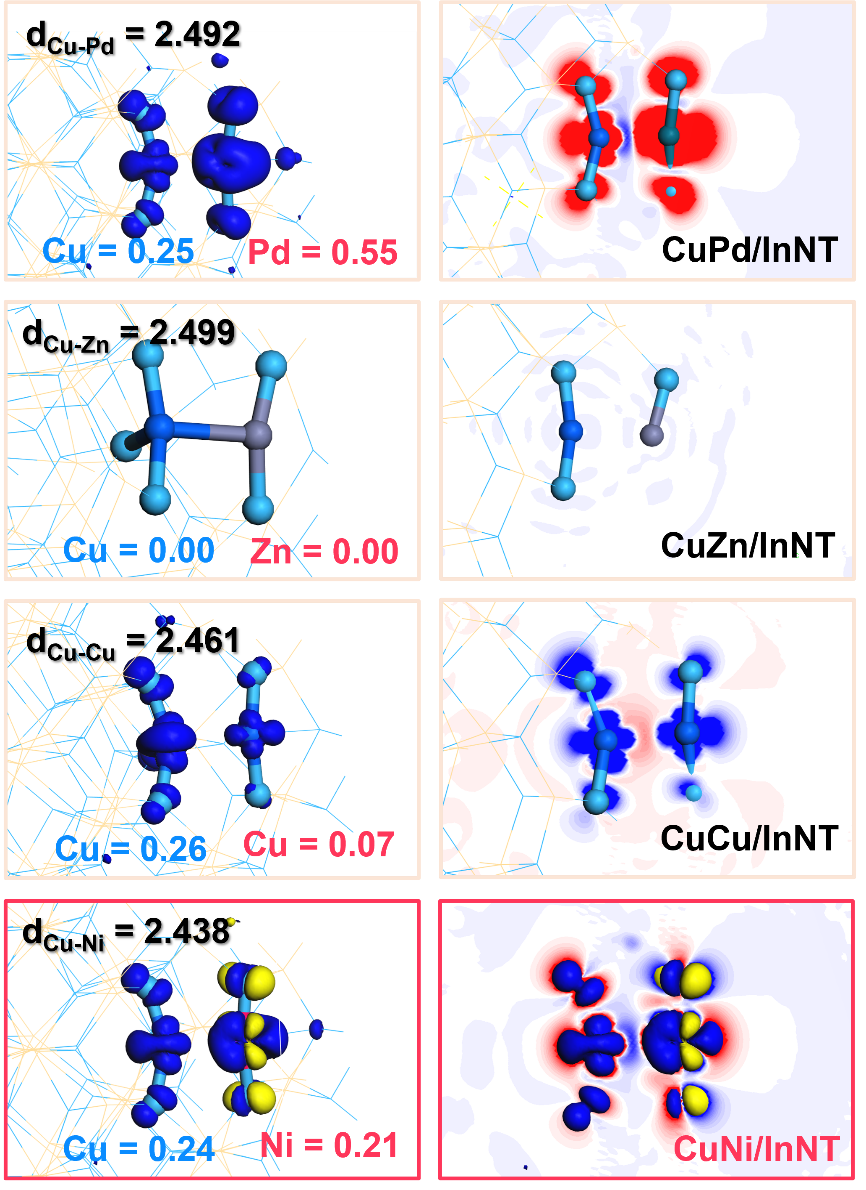


**Supplementary Figure 28: Calculated spin density results of CuPd/InNT, CuZn/InNT, CuZn/InNT, and CuNi/InNT.** Isosurface = 0.01


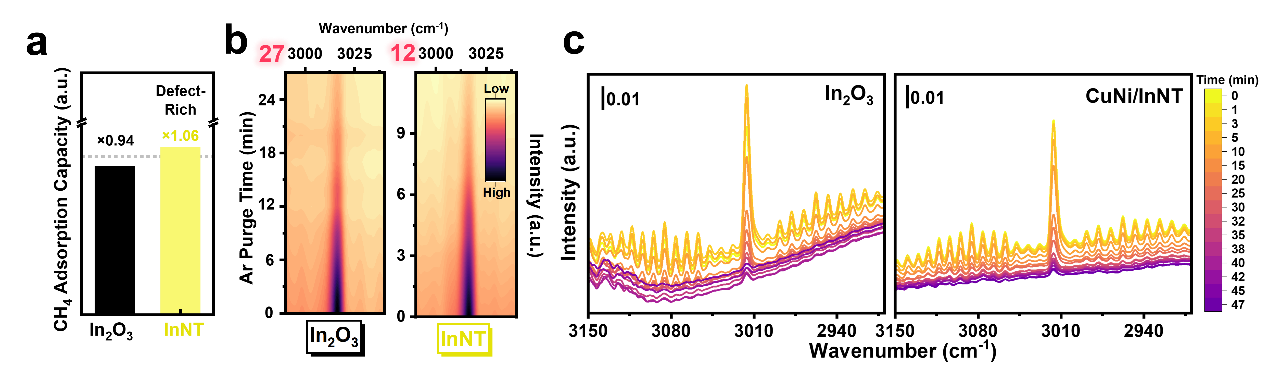


**Supplementary Figure 29: The partial CH_4_-DRIFTS results of samples**. **a**, semi-quantitative analysis for CH_4_ adsorption response of In_2_O_3_ and InNT. **b**, CH_4_ adsorption intensity using Ar purge time as the descriptor. **c**, the zoomed area of *CH_4_ in the DRIFTS spectrum of In_2_O_3_ and CuNi/InNT.

Lattice distortion and abundant defect sites in InNT significantly reduce CH_4_ adsorption response. This may be attributed to active site dilution. As if defect sites were unevenly distributed on the surface, it could lead to effective adsorption site reduction, thereby increasing the adsorption response or decreasing the adsorption capacity. It also could be due to the mismatch of adsorption sites and CH_4_ molecules since the defect sites may present in different sizes or chemical properties that may not be sensitive to CH_4_. The shifts of the d-band center could also result in a similar phenomenon because of strain and defect sites, leading to charge density redistribution near defects. This inference was consistent with the subsequent calculations showing a decrease in In-PDOS in InNT.

In Fig. S25b-c, the CH_4_ adsorption intensity was analyzed using the duration from the most intense adsorption peak to a 90% reduction upon Ar purge after CH_4_ fully adsorption as the descriptor. InNT exhibited the weakest adsorption strength, potentially due to the presence of defects that alter the electronic structure and chemical properties of metal sites, thereby affecting their adsorptive capacity for CH_4_ molecules.


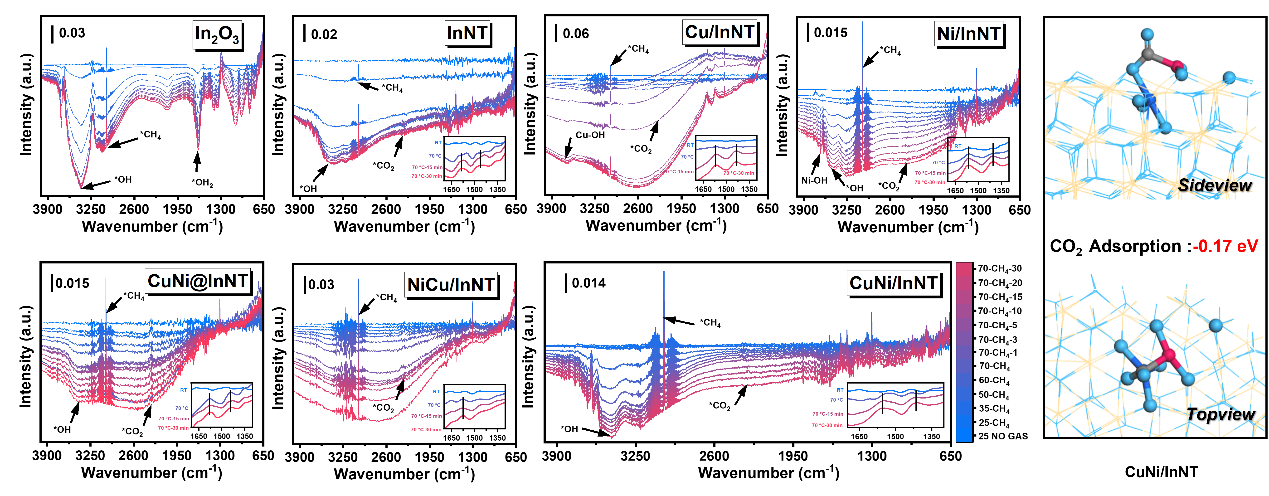


**Supplementary Figure 30: The overall CH_4_-DRIFTS results of samples**. The temperature ramping program was to increase the temperature from room temperature to 70 ℃ under the premise introduction of 2 ppm CH_4_ and maintain it at 70 ℃ for 30 minutes.

In_2_O_3_ exhibited substantial adsorbed H_2_O, correlating with its strong hydrophilicity, which may lead to a decrease in reaction activity due to water occupying active sites. In contrast, all InNT-based catalysts show a significant reduction in this peak, indicating an increasing hydrophobicity. Cu/InNT and NiCu/InNT samples demonstrated the best hydrophobic performance. Combined with product analysis, this hydrophobic feature may promote HCOOH production. Some CO_2_-related intermediate signals were also observed (*COOH, *HCO_3_, *CO_3_, etc.) which came from adsorbing atmospheric CO_2_ due to the strong affinity of InNT and Cu-Ni active species for CO_2_ (which was calculated to be -0.17 eV over CuNi/InNT, evidencing its auto-adsorption)^37, 38^. In particular, the monometallic Cu/InNT and Ni/InNT samples showed high signals suggesting an exceptional CO_2_ adsorption and conversion capability, which may hinder MTM at elevated temperatures, underscoring the importance of bimetallic modification.


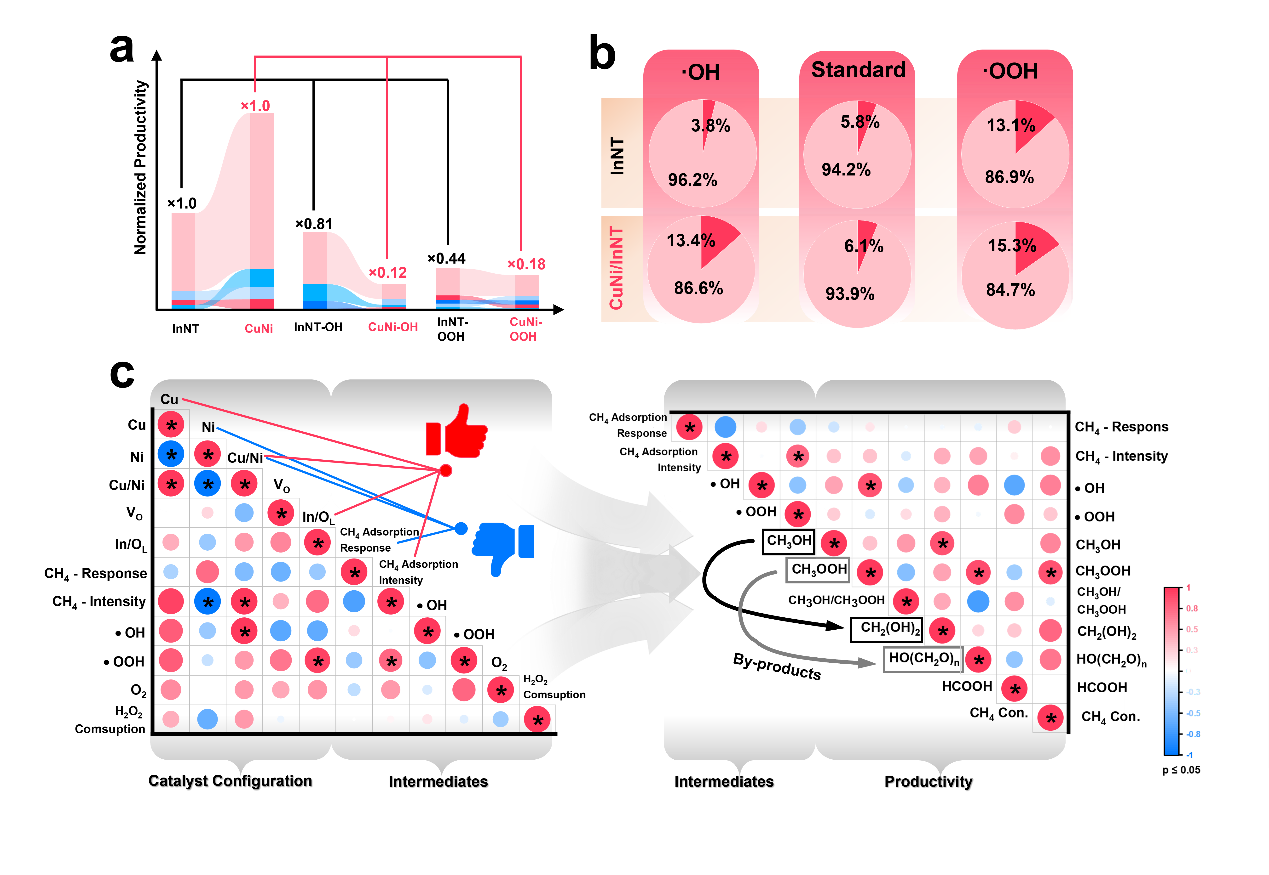


**Supplementary Figure 31: Free radical quenching experiments. a**, yield comparison of free radical quenching experiments on InNT and CuNi/InNT. Note that the yields were normalized based on the yield obtained under standard conditions. **b**, the CH_3_OH/CH_3_OOH ratio of free radical quenching experiments on InNT and CuNi/InNT. **c**, correlation analysis of structure-activity relationship.

InNT maintained 81% of its yield after quenching ·OH, while only 44% was remained after quenching ·OOH, indicating that the ·OOH predominated the formation of CH_3_OOH on InNT. This was further supported by DFT calculations, which show that the presence of adjacent In and O vacancies in InNT indeed favors the generation of ·OOH from H_2_O_2_. Additionally, a significant decrease in yield was observed after quenching ·OOH in CuNi/InNT, suggesting that ·OOH also played a substantial role.

The changes in CH_3_OH/CH_3_OOH ratio on InNT after radical quenching shifts as anticipated, which was quenching ·OH reduced the proportion of CH_3_OH while quenching ·OOH diminished the proportion of CH_3_OOH. This indicated that both pathways can coexist in parallel. Additionally, on CuNi/InNT samples, quenching ·OOH also led to a decrease in CH_3_OOH, suggesting that the pathway involving CH_4_ combining with ·OOH to form CH_3_OOH was also present in CuNi catalysts.

The yield of CH_3_OH in quenching experiments was analyzed (Table S1). After quenching · OH, the yield of methanol decreased by about 79.3% (6500.68 μmol/g_cat_/h → 1346.88 μmol/g_cat_/h), which was largely attributed to the direct effect on *CH_3_ + ·OH → CH_3_OH pathway. However, a methanol yield of 20.7% was still retained. Similarly, when quenching ·OOH, theoretically the CH_3_OH pathway should be merely. However, the actual value decreased to 2365.21 μmol/g_cat_/h, indicating that ·OOH has a secondary contribution to CH_3_OH.


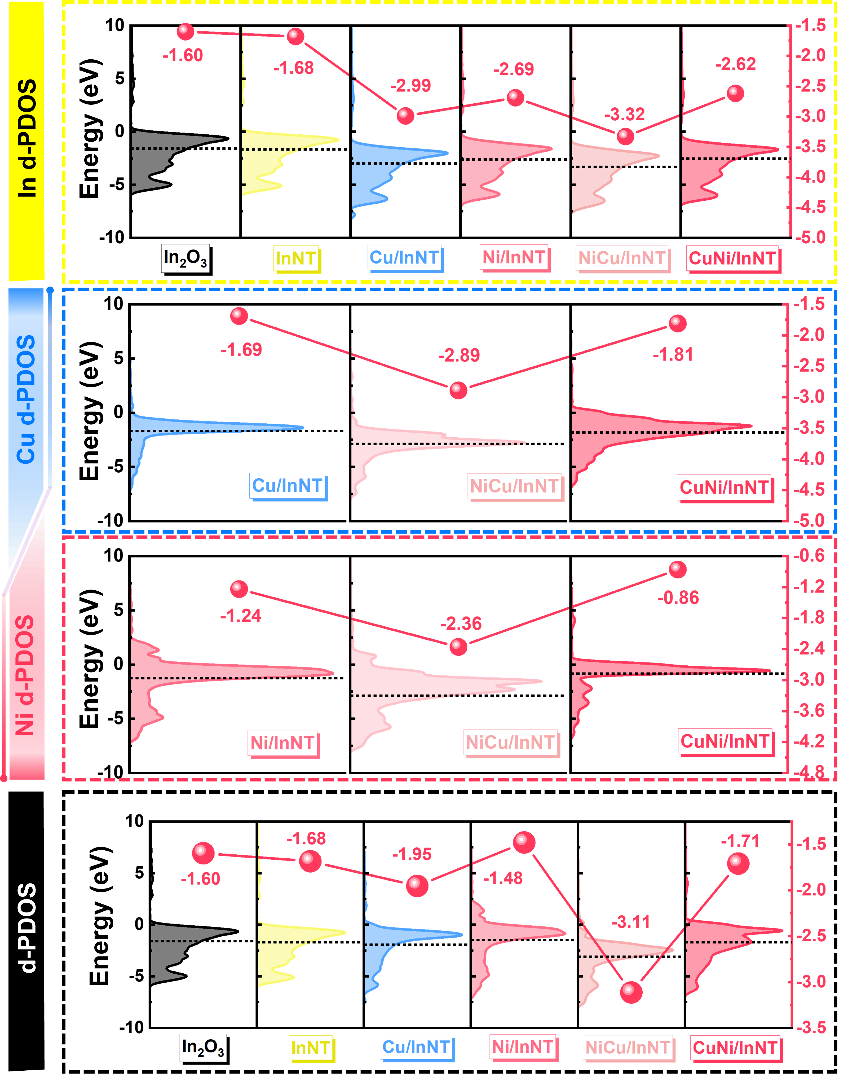


**Supplementary Figure 32: The d-band centers of InNT catalysts.**

In’ d-band center was lower in InNT compared to In_2_O_3_. This was due to the uneven distribution of charges originating from the crystal distortion-induced defects and strain. After loading active metals, they all deceased, suggesting possible charge transfer from In support to active metals, forming a more electron-rich active site configuration, leading to downshifts of the d-band centers. Up to the 3d orbits of Cu and Ni, it was found that the Cu d-band center in the NiCu/InNT sample was the lowest, resulting in the most sluggish adsorption of substrates, causing insufficient activation. Specifically, the total a-band center caused by d-d hybridization was abnormally high over Ni/InNT. This phenomenon indicated a strong interaction between substrates molecular and the catalysts, hindering timely desorption, consistent with its highest O_2_ formation rate.


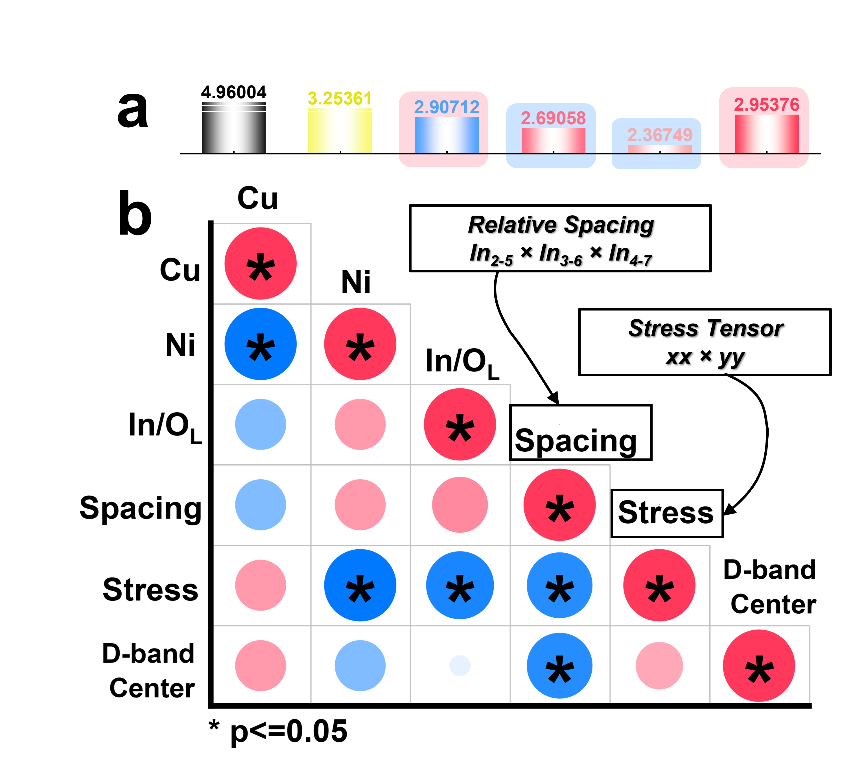


**Supplementary Figure 33: In-depth correction analysis. a**, stress indicator using xx × yy from calculated stress tensor. **b**, correction analysis of Cu, Ni, V_In_, spacing, stress, and d-band center.

Overall, the presence of Cu was beneficial for stress increase (more compression), while the presence of Ni significantly leads to tension. In/O_L_ can be used as an indicator of V_In_ as the lower the In/O_L_ suggested more V_In_. The presence of V_In_ reduced the relative distance, strengthened the compressive strain, and increased the d-band center. The spacing, stress, and d-band center were significantly correlated with each other, especially the most important point was that larger spacing led to more stretching and the lower d-band center, which was unfavorable for the MTM reaction. Therefore, the CuNi/InNT sample with the most compressed strain had the best CH_3_-R productivity.


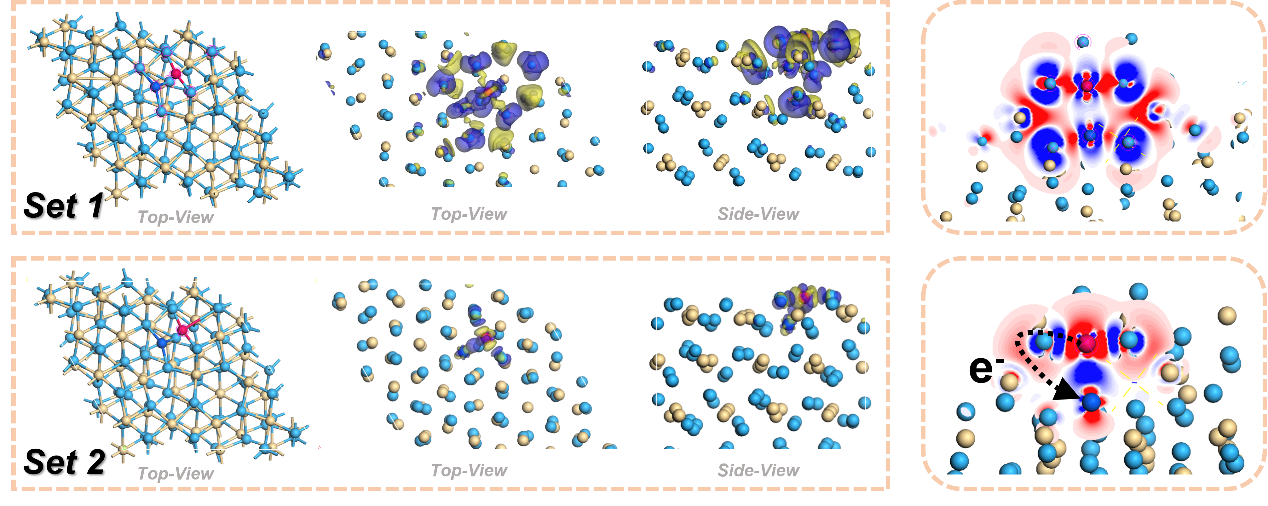


**Supplementary Figure 34: Charge density difference diagram of CuNi/InNT.** In the first column of Fig. 30a-b, blue represents obtaining electrons, yellow represents donating electrons

Firstly, Cu, Ni, and surrounding O were selected as set, and it was found that the surrounding carrier In would transfer electrons to the Cu-Ni active site, which was consistent with the XPS results. Then, the single Ni atom was used as a separate set for analysis, it was found that Ni would transfer charges to Cu.


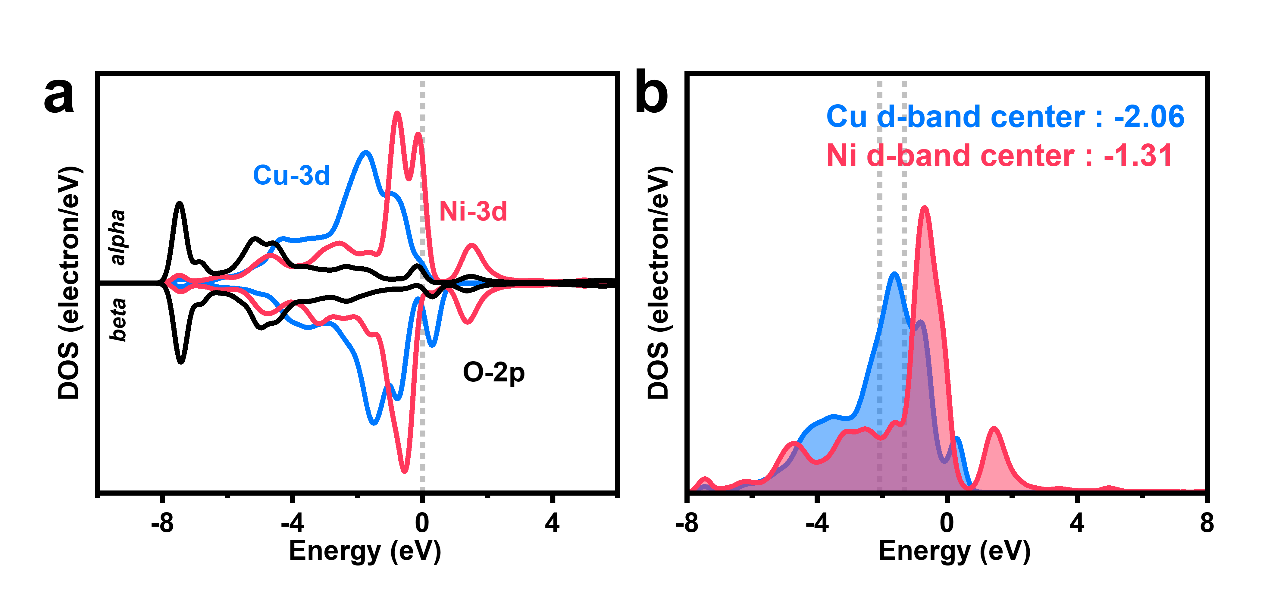


**Supplementary Figure 35:** **The orbital situations of CuNi/InNT in step ii. a**, the specific alpha and beta orbitals of Cu-3d, Ni-3d, and the O-2p in bridged *OH. **b**, the d-band center of Cu and Ni.


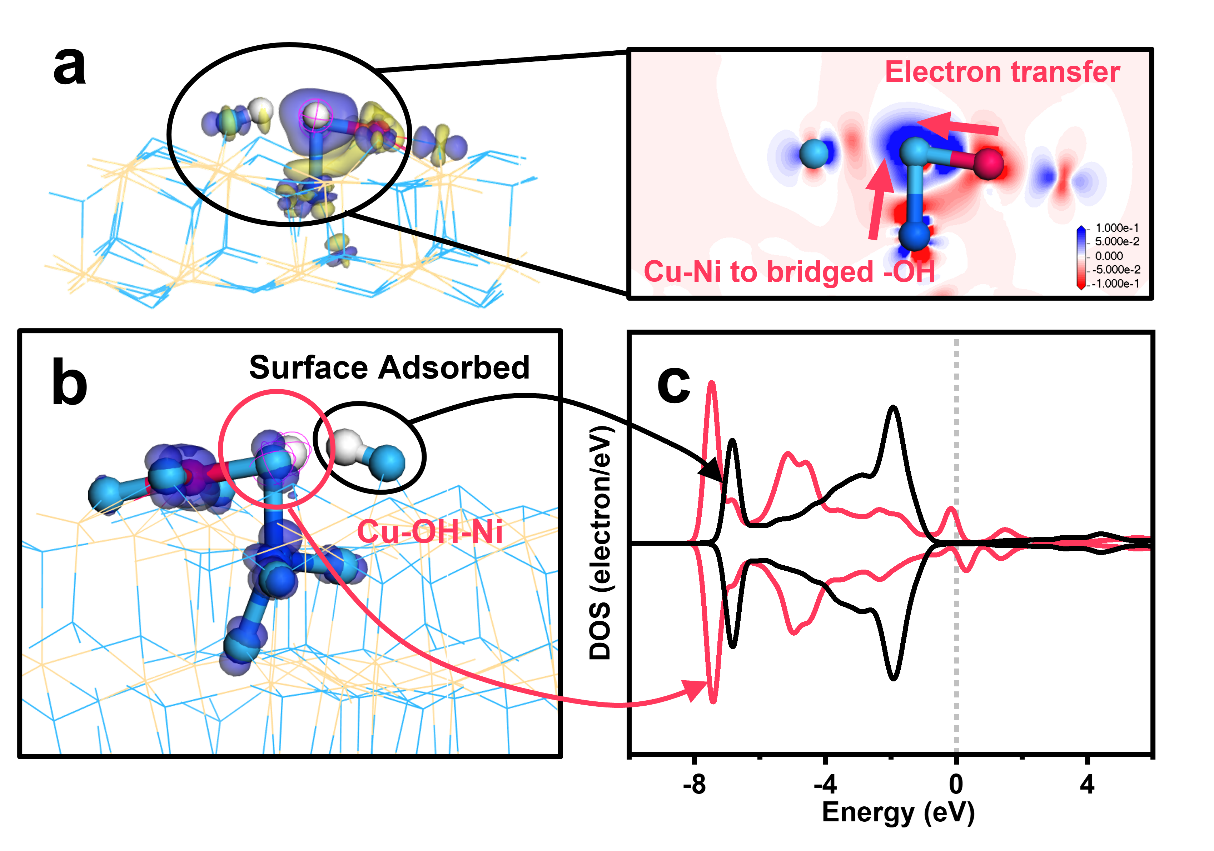


**Supplementary Figure 36: The calculated differential charge density, spin density and orbital difference of O in Cu-OH-Ni and surface adsorbed *OH in step ii. a**, the charge density diagram using the -OH species in Cu-OH-Ni configuration as set. **b**, the spin density diagram. **c**, the specific alpha and beta orbitals of both O-2p.


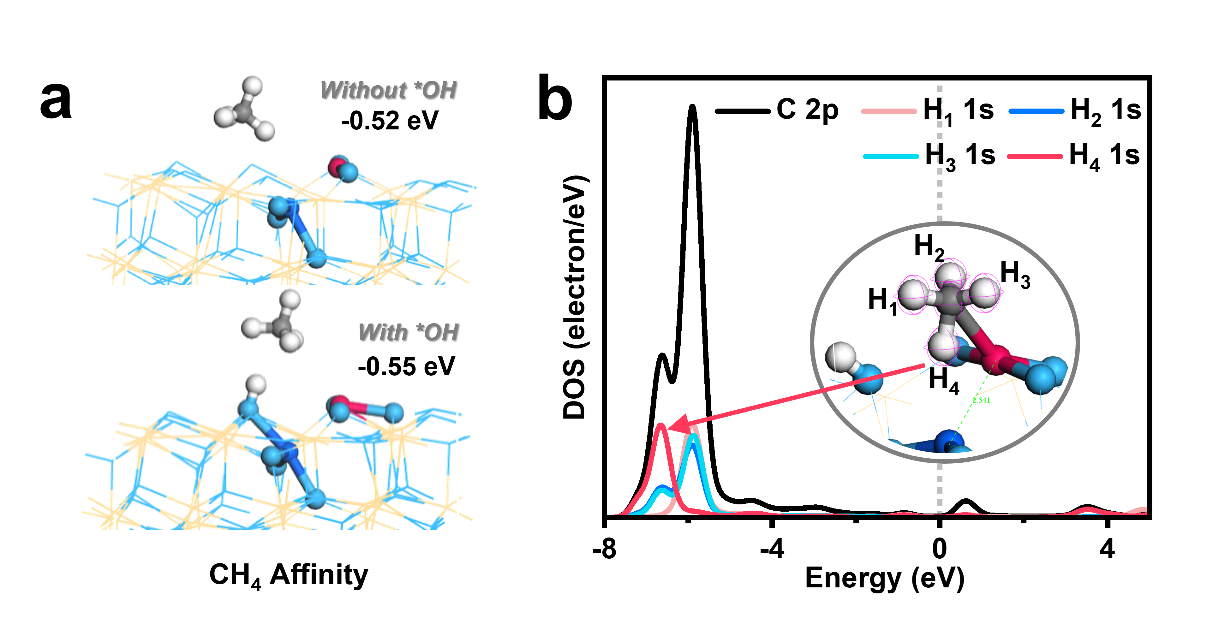


**Supplementary Figure 37: The CH_4_ affinity with or without *OH from H_2_O_2_ decomposition and the overlap situation of C-2p and H-1s total DOS in step v.**

The adsorption of CH_4_ and the decomposition of H_2_O_2_ were not entirely independent processes. They were also likely to occur sequentially at the same active site, with H_2_O_2_ adsorption before CH_4_. This precedence was attributed to the lower adsorption barrier of H_2_O_2_ (-3.55 eV), compared to the CH_4_ affinity of -0.52 eV. Upon adsorption, H_2_O_2_ generates hydroxyl groups (*OH) on the catalyst surface, which in turn further reduce the adsorption barrier for CH_4_ to -0.55 eV. Collectively, these findings indicated that during the adsorption and activation of the two key substrates, CH_4_ and H_2_O_2_, not only do parallel reactions occur, but sequential reactions were also plausible, with H_2_O_2_ preferentially adsorbing at the site before CH_4_.

\
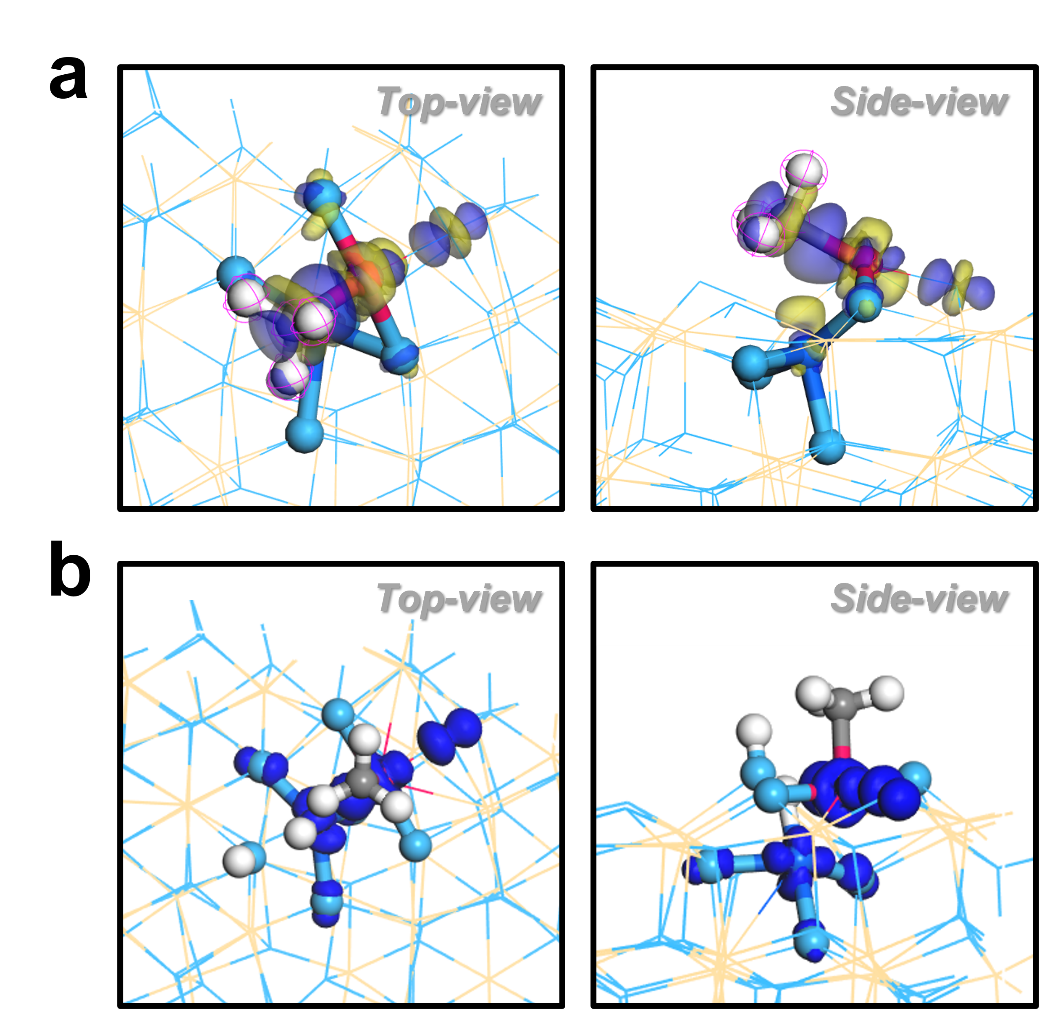


**Supplementary Figure 38: a, the charge density difference using *CH_3_ as set in step vii. b, the spin density results of *CH_3_.** Blue represents obtaining electrons, yellow represents donating electrons. And the spin density isosurface = 0.02.


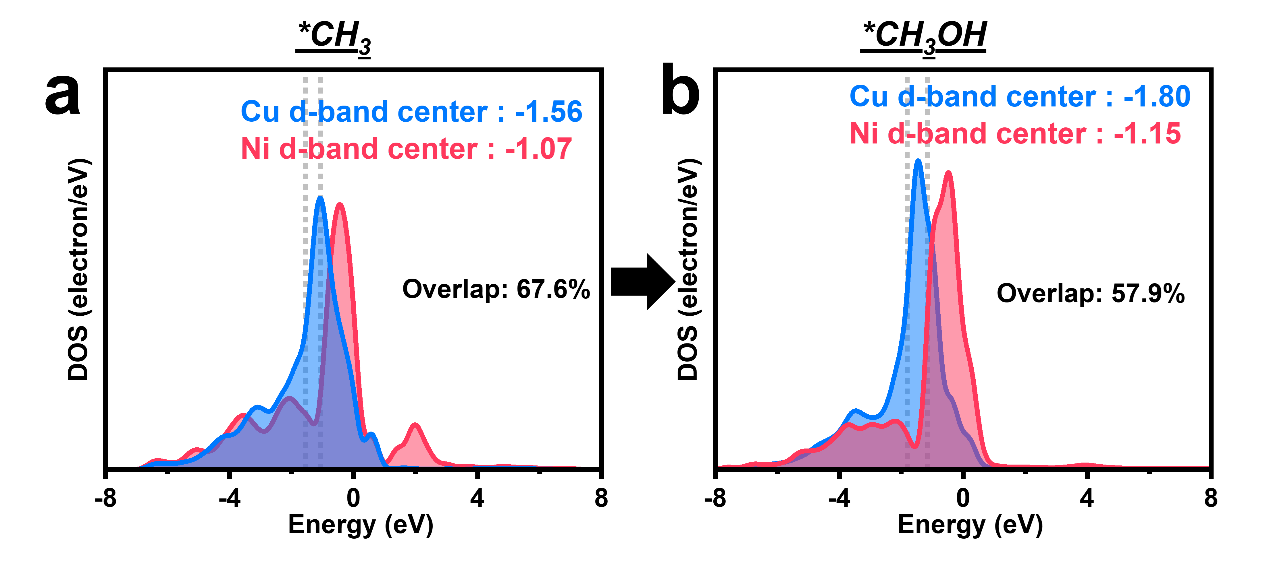


**Supplementary Figure 39: The d-band shift of Cu and Ni in steps vii and viii. a**, the d-band situation in CuNi/InNT-*CH_3_ with calculated Cu-Ni d-band overlap. **b**, the d-band situation in CuNi/InNT-*CH_3_OH with calculated Cu-Ni d-band overlap.


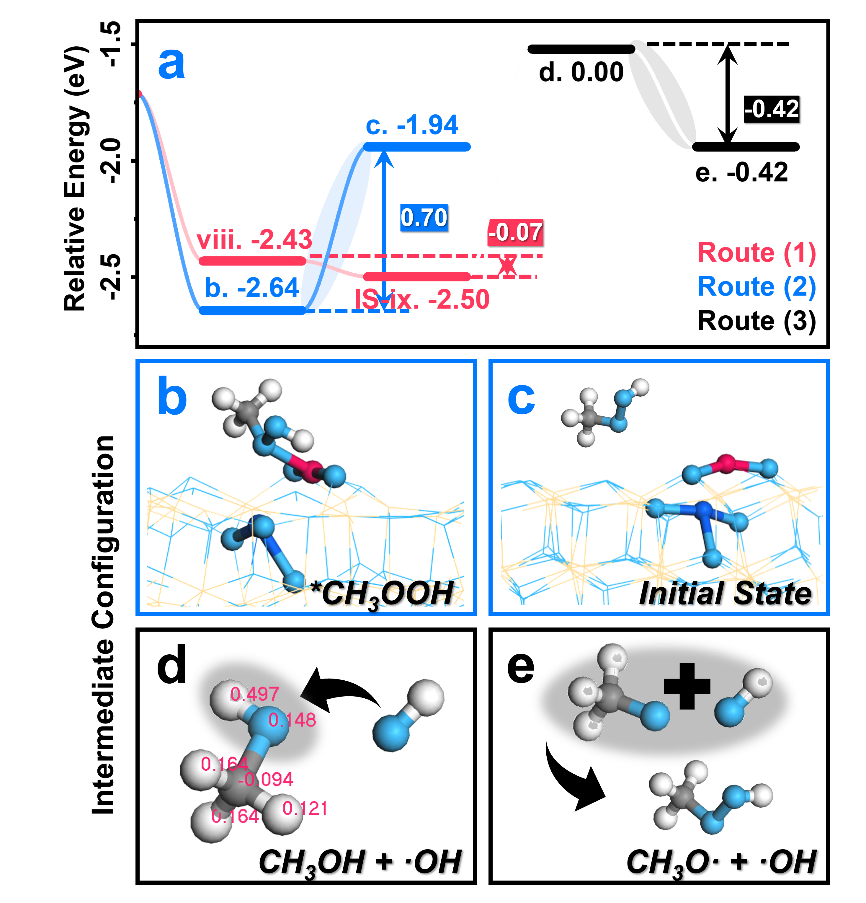


**Supplementary Figure 40: The following reaction steps of CH_3_OH and CH_3_OOH. a**, energy step diagram of CH_3_OH and CH_3_OOH formation and desorption. The right black steps were the energy gap for CH_3_OH to CH_3_OOH in the solution. **b-c**, illustration of *CH_3_OOH adsorbed and desorbed from CuNi/InNT. d, calculated radical Fukui function over CH_3_OH. **e**, illustration of CH_3_O· combined with ·OH to form CH_3_OOH.


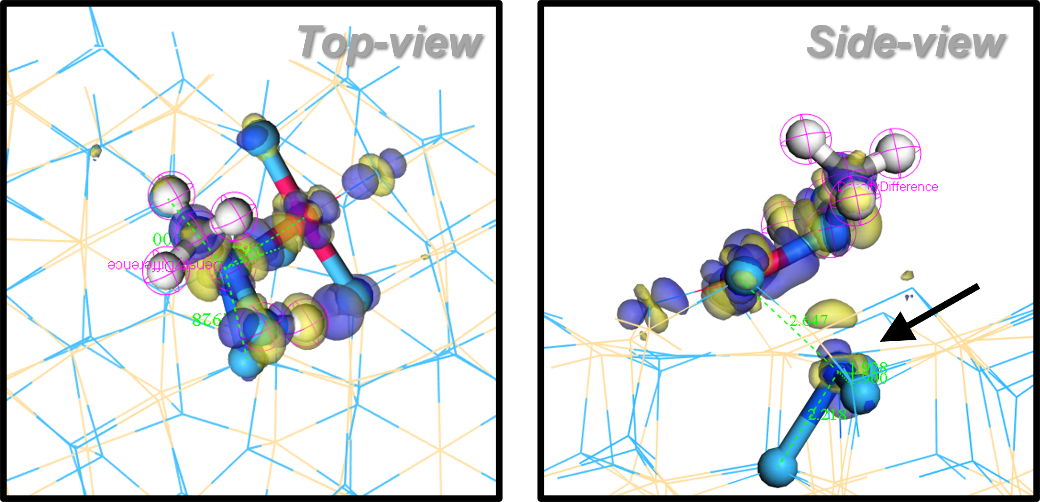


**Supplementary Figure 41: The charge density difference using *CH_3_OOH as set.** Blue represents obtaining electrons, yellow represents donating electrons.


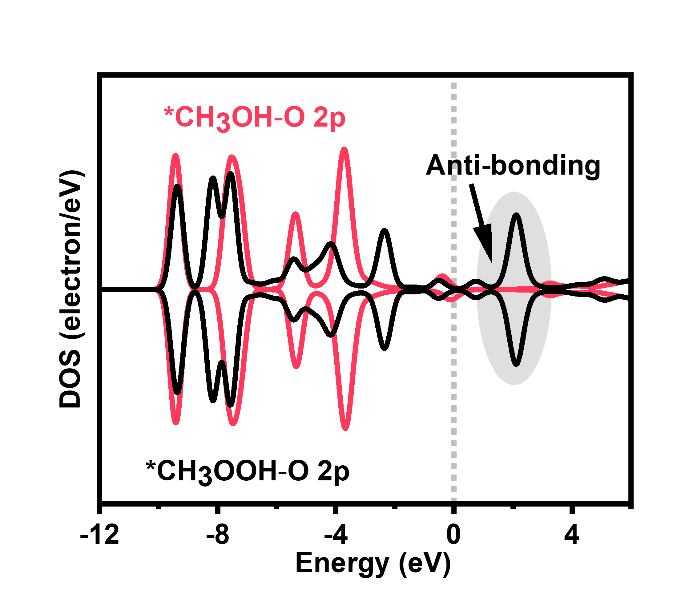


**Supplementary Figure 42: O-2p orbital situations of *CH_3_OH and *CH_3_OOH.**


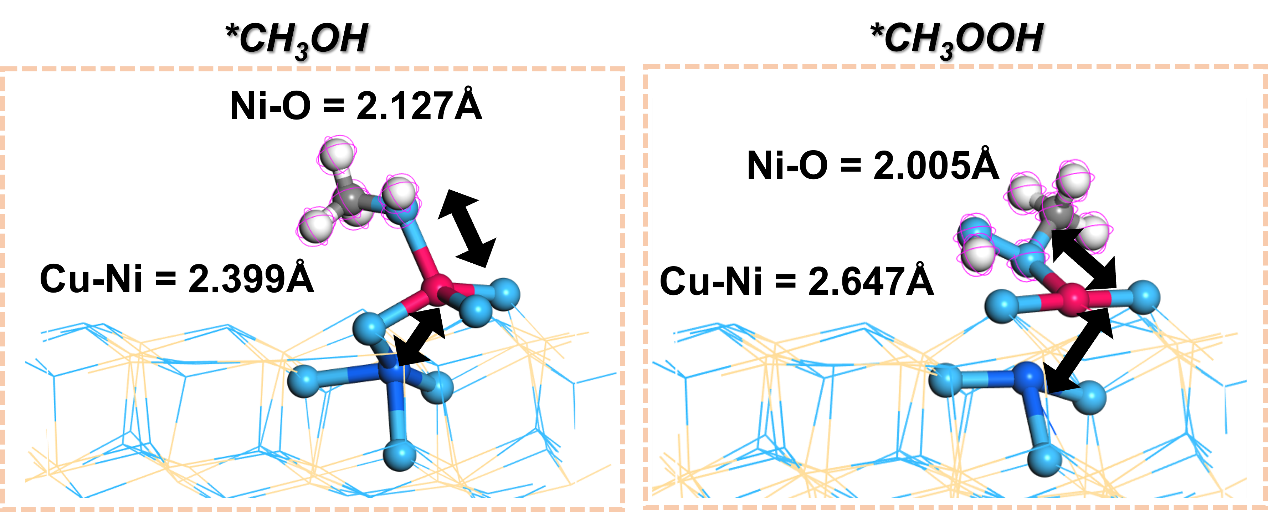


**Supplementary Figure 43: Calculated bond length of *CH_3_OH and *CH_3_OOH.**


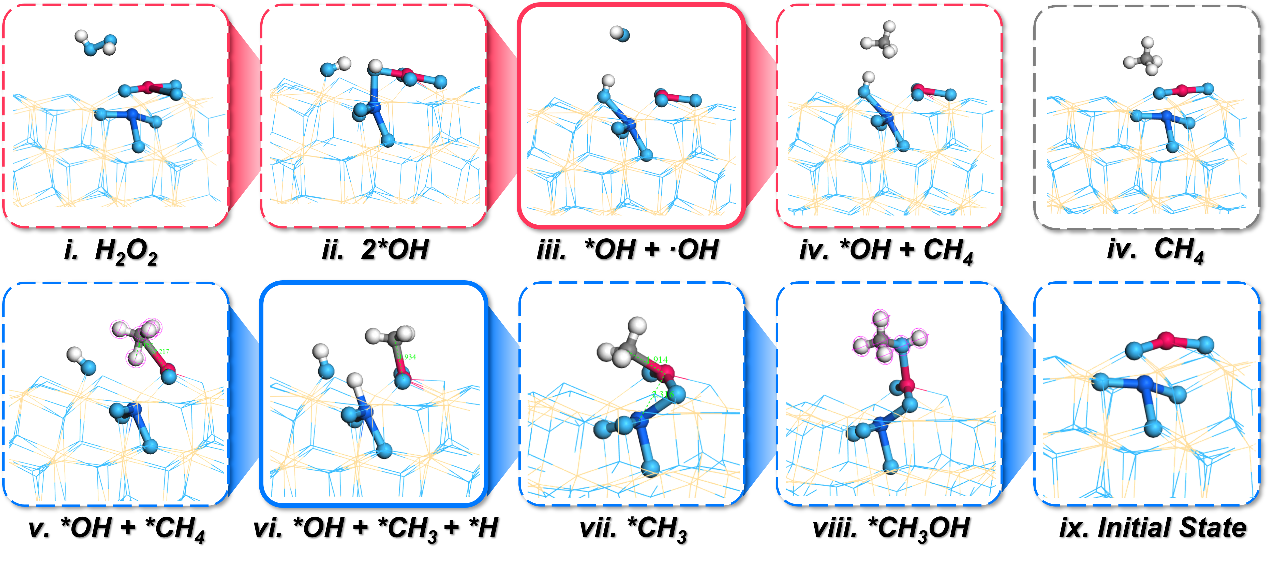


**Supplementary Figure 44: Illustration of involved intermediates.**


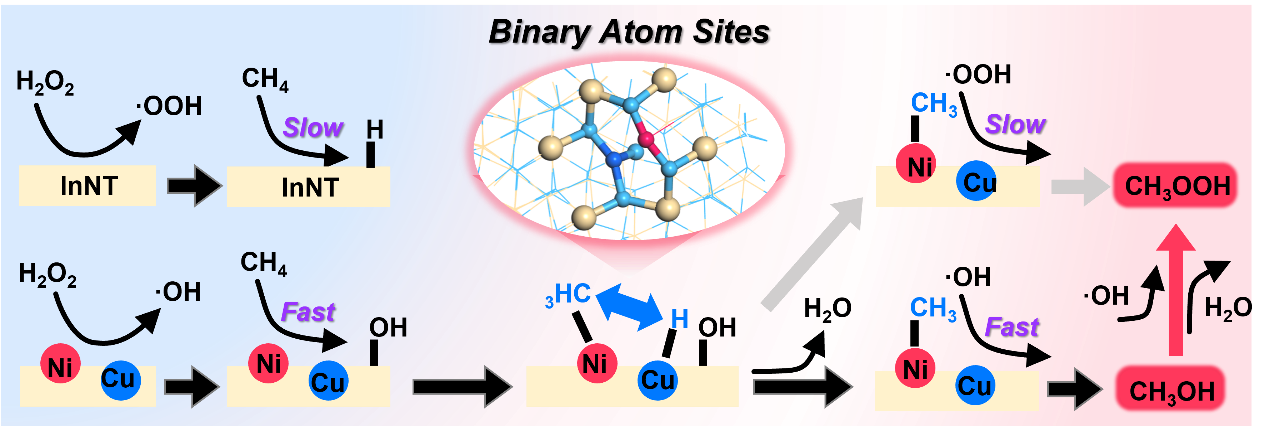


**Supplementary Figure 45: Illustration of all reaction pathways.**


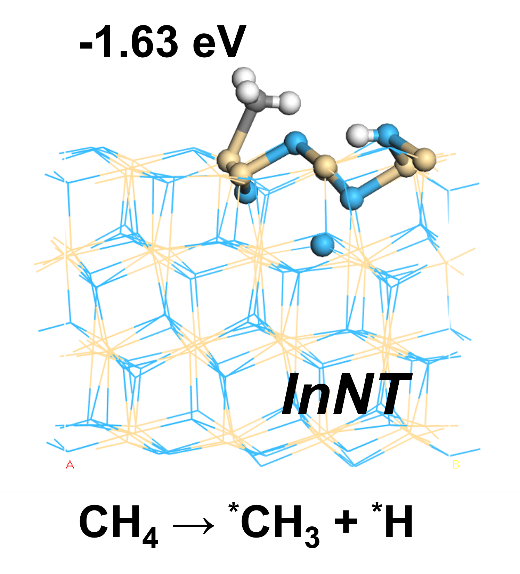


**Supplementary Figure 46: Illustration of CH_4_ dissociation over InNT.**


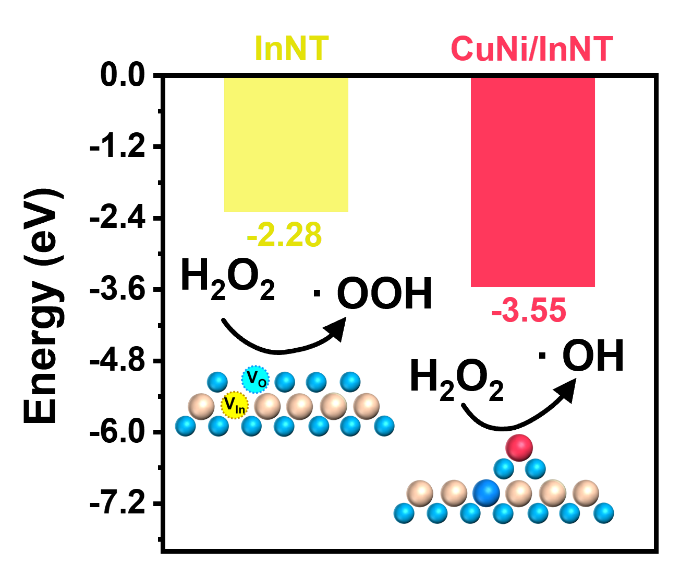


**Supplementary Figure 47: Calculated dissociation of H_2_O_2_ over InNT and CuNi/InNT.**


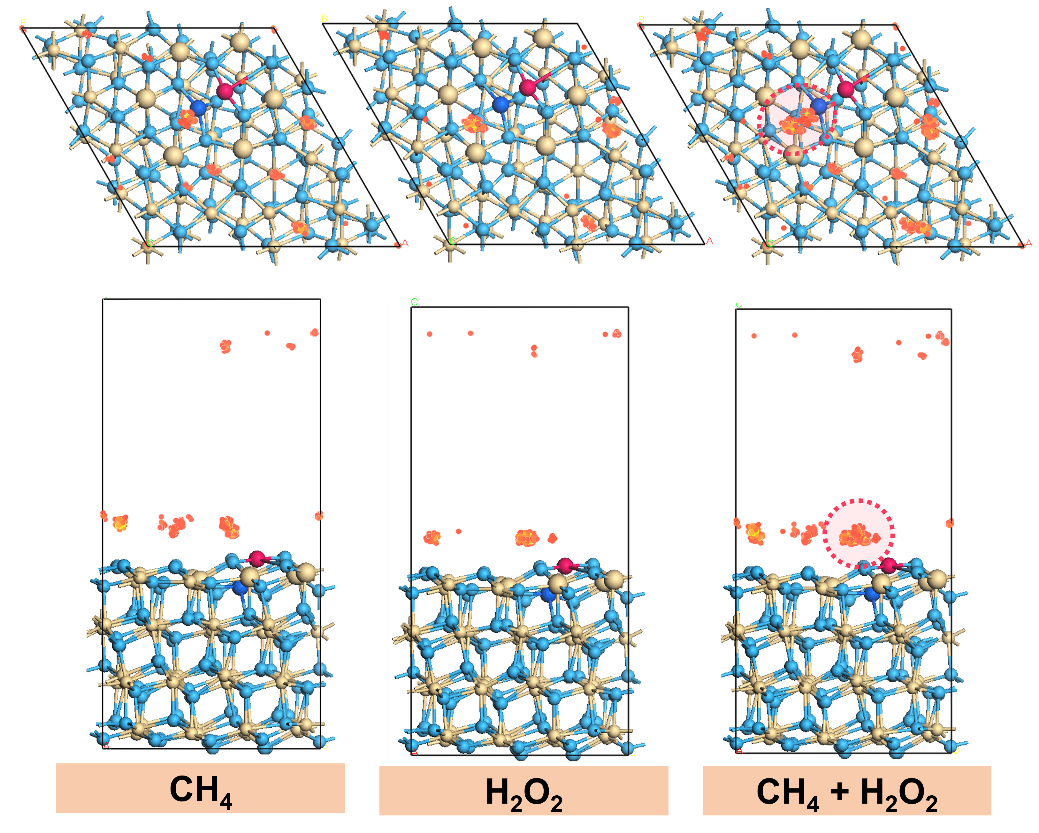


**Supplementary Figure 48: The adsorption location field of CH_4_, H_2_O_2_, and CH_4_+H_2_O_2_ over CuNi/InNT.**


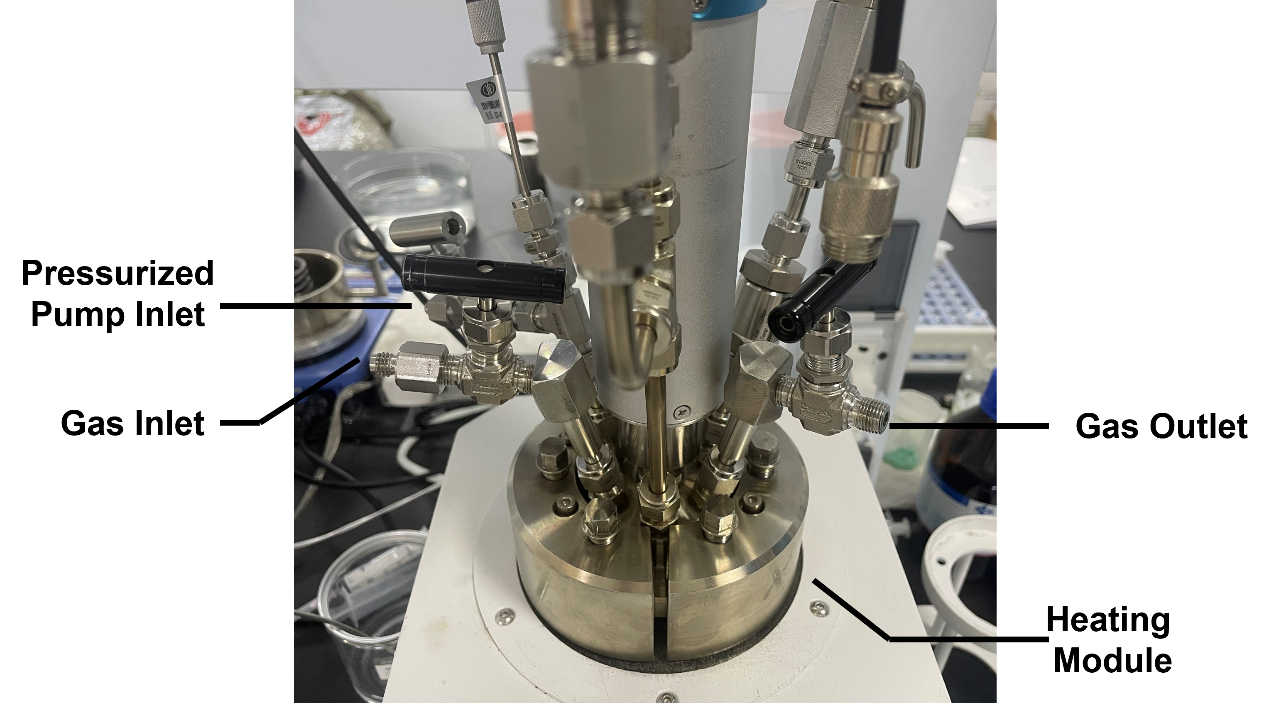


**Supplementary Figure 49: The photograph of 100 mL continuous H_2_O_2_ injection reactor.**

The reaction conditions for 100 mL reactor: 30 bar, 70 °C, 800 rpm, H_2_O_2_ injection rate at 1 mL/min.


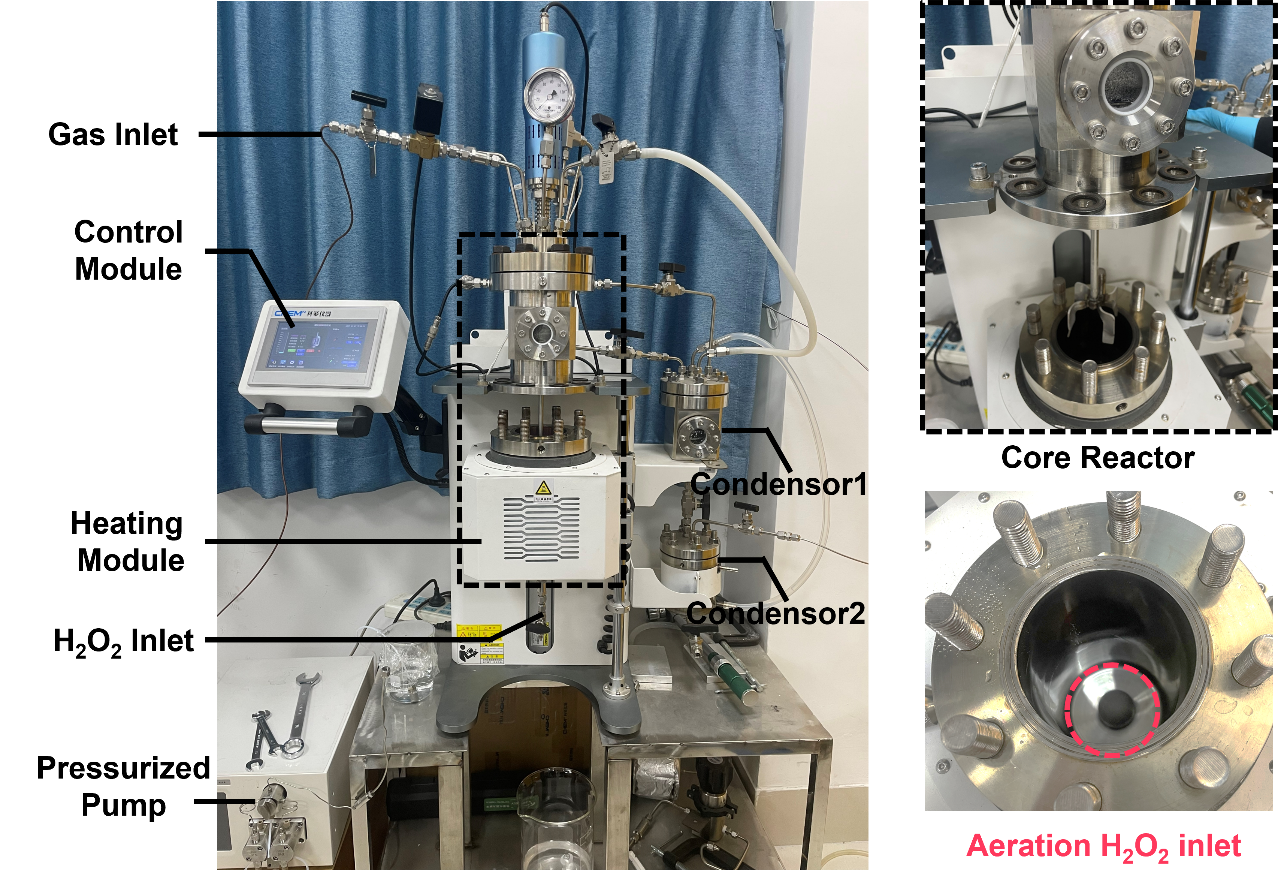


**Supplementary Figure 50: The photograph of 1000 mL continuous H_2_O_2_ injection reactor.**

The reaction conditions for 1000 mL reactor: 15 bar, 70 °C, 500 rpm, H_2_O_2_ injection rate at 6 mL/min.

**Supplementary Table 1. Detailed catalytic performance of In-based catalysts**

| **Entry** | **Sample** | **Productivity (μmol/g_cat_/h)** | | | | | | **CH_3_OH Selectivity**  **(%)** | **CH_3_-R Selectivity (%)** | **CH_3_OH/CH_3_OOH Ratio (%)** | **CH_4_ conversion rate (‰)^b^** | **O_2_ (μmol/h)** | **H_2_O_2_ Comsuption (%/mg)** |
| --- | --- | --- | --- | --- | --- | --- | --- | --- | --- | --- | --- | --- | --- |
|  |  | **CH_3_OH** | **CH_3_OOH** | **CH_2_(OH)_2_** | **HO(CH_2_O)_n_** | **HCOOH** | **CH_3_-R ^a^** |  |  |  |  |  |  |
| 1 | In_2_O_3_ | 4474.77 | 48461.97 | 4880.37 | 3679.76 | b.d.**^c^** | 52.94 | 7.28 | 86.08 | 9.23 | 5.17 | 8.32 | 14.45 |
| 2 | InMOF | 3976.77 | 33369.06 | 1517.88 | 3613.44 | 11363.68 | 37.35 | 7.39 | 69.36 | 11.92 | 4.49 | 6.90 | 14.47 |
| 3 | InNT | 3045.37 | 49597.01 | 2987.20 | 5725.31 | b.d.**^c^** | 52.64 | 4.96 | 85.80 | 6.14 | 4.58 | 6.98 | 16.32 |
| 4 | Cu/InNT | 3470.41 | 51948.81 | 9184.68 | 6410.90 | 7579.87 | 55.42 | 4.42 | 70.51 | 6.68 | 6.09 | 9.72 | 15.51 |
| 5 | Ni/InNT | 2490.35 | 38568.21 | 2300.05 | 4630.98 | 1035.36 | 41.06 | 5.08 | 83.75 | 6.46 | 3.76 | 9.04 | 15.83 |
| 6 | NiCu@InNT | 4144.03 | 32873.16 | 6653.58 | 1021.56 | 1291.55 | 37.02 | 9.01 | 80.50 | 12.61 | 3.68 | 10.74 | 15.29 |
| 7 | NiCu/InNT | 4908.88 | 43029.15 | 9216.81 | 1207.51 | 7768.52 | 47.94 | 7.42 | 72.49 | 11.41 | 5.24 | 14.25 | 15.23 |
| **8** | ***CuNi/InNT*** | ***6500.68*** | ***99499.48*** | ***11462.95*** | ***7906.46*** | ***b.d.^c^*** | ***106.00*** | ***5.19*** | ***84.55*** | ***6.53*** | ***9.77*** | ***10.88*** | ***15.63*** |
| 9 | Blank | b.d.**^c^** | b.d.**^c^** | b.d.**^c^** | b.d.**^c^** | b.d.**^c^** | - | - | - | - | - | 6.15 | - |
| 10 | InNT-N_2_ | b.d.**^c^** | b.d.**^c^** | b.d.**^c^** | b.d.**^c^** | b.d.**^c^** | - | - | - | - | - | 7.69 | 17.82- |
| 11 | InNT-OH | 1221.99 | 30549.84 | 9994.16 | b.d.**^c^** | 4364.26 | 31.77 | 2.65 | 68.87 | 4.00 | 3.72 | 6.37 | 15.03 |
| 12 | InNT-OOH | 2587.75 | 17094.09 | 2127.43 | 2239.40 | 2488.22 | 19.68 | 9.75 | 74.17 | 15.14 | 2.02 | 7.18 | 15.99 |
| 13 | CuNi/InNT-Without H_2_O_2_ | 348.33 | 487.66 | 288.54**^d^** | b.d.**^c^** | b.d.**^c^** | 0.84 | 30.98 | 74.34 | 71.43 | 0.08 | 5.22 | - |
| 14 | CuNi/InNT-OH | 1346.88 | 8674.82 | 1632.24 | 3298.71 | 570.71 | 10.02 | 11.56 | 69.57 | 15.53 | 1.16 | 6.45 | 16.50- |
| 15 | CuNi/InNT-OOH | 2365.21 | 13125.62 | 1364.54 | 2807.06 | 2599.13 | 15.49 | 14.03 | 80.79 | 18.02 | 1.80 | 6.90 | 15.13 |

**a**, CH_3_-R stands for methyl oxygenates (the addition of CH_3_OH and CH_3_OOH), and its unit was mmol/g_cat_/h. **b**, The unit of ‰ was used instead of % to give a more detailed description of the calculated results. **c**, Below detection limit. **d**, Stands for the yield of CH_3_CH_2_OH instead of CH_2_(OH)_2_.

All yields were obtained under standard reaction conditions except the following entries. In entry 9, no catalyst was added into the batch reactor. Only a trace amount of O_2_ was detected, possibly due to H_2_O_2_ auto-decomposition during temperature rises. In entry 10, N_2_ instead of CH_4_ was injected into the batch reactor to exclude the possibility of C in InNT as CH_3_OH’s carbon source. In entries 11 and 14, 100 μL of isopropanol was added as ·OH scavenger. In entry 12 and 15, 1 mL of p-benzoquinone was added as ·OOH scavenger. In entry 13, no H_2_O_2_ was added into the batch reactor. A trace amount of CH_3_CH_2_OH was formed, suggesting C-C coupling was triggered due to the lack of oxidizing agent.

Upon the CH_3_OH/CH_3_OOH ratio and total yield, the Cu/InNT sample showed an increase compared to InNT. While for Ni/InNT this ratio further decreased compared to Cu/InNT which might be due to the deposited NiO clusters inducing more oxygen defects which cause H_2_O_2_ to dissociate to ·OOH instead of ·OH. The overall yield of CH_3_OH and CH_3_OOH in NiCu/InNT sample was 1.3 times that of NiCu@InNT indicating that the active sites generated by bimetallic modification in this case can also affect the generation of ·OH and ·OOH, specifically a great amount of ·OOH was formed over this sample which was validated by EPR. CuNi/InNT sample showed a significant increase in CH_3_OH and CH_3_OOH yield which was up to 2.9 times compared to NiCu@InNT. Yet the CH_3_OH/CH_3_OOH ratio further decreased again, mainly due to the significant increase in the yield of CH_3_OOH caused by the special active sites. But whether this active site configuration was able to increase ·OH or ·OOH could not be determined at this point.

The decomposition of H_2_O_2_ was more pronounced on InNT as compared to In_2_O_3_, while the production of O_2_ was reduced, indicating that the defect sites present on this support were more conducive to the decomposition of H_2_O_2_ into free radicals. The O_2_ yield follows the order of bimetallic > monometallic > pure support, with both bimetallic catalysts exceeding a threshold of 10 μmol/h, which was in line with the pattern that transition metals facilitate the decomposition of H_2_O_2_.

Nevertheless, despite the integral activity of Cu/InNT has been elevated, its CH3-R selectivity was the lowest among all tested samples (70.51%), with a 14% diminutive ascent in CH3OH productivity compared to InNT (3470.41 vs 3045.37 μmol/gcat/h). It is to say that all upgrades came from the unlikely exploitable and less stable POM and CH2(OH)2, to crown all, HCOOH with a yield doubled from that of CH3OH was also formed (7579.87μmol/gcat/h). Previous studies in CO2 electrochemical reduction revealed that the Cu-doped Indium catalysts would accelerate the formation of *COOH intermediate52,53.

**Supplementary Table 2. Catalytic performance of MTM conversion over recently reported promising catalysts in the liquid phase.**

| **Entry** | **Catalyst** | **Reaction Conditions** | | | | **Productivity (μmol/g**cat/h) | | | **Reference** |
| --- | --- | --- | --- | --- | --- | --- | --- | --- | --- |
|  |  | **Reaction Type** | **Oxidant** | **Pressure (bar)** | **Temperature (**oC) | CH3OH | CH3OOH | **CH_3_-R Selectivity** **(%)^a^** |  |
| ***1*** | ***CuNi/InNT*** | ***Thermo-catalysis*** | ***H*2O2** | ***30*** | ***70*** | ***6500.68*** | ***99499.48*** | **84.55** | ***This work*** |
| 2 | Cu-Fe/ZSM-5 | Thermo-catalysis | H2O2 | 30 | 50 | 7985.60 | 614.85 | 86.15 | ^39^ |
| 3 | Cu1/ZSM-5 | Thermo-catalysis | H2O2 | 30 | 70 | ~6000.00 | -**^d^** | ~50.00 | ^40^ |
| 4 | Rh/ZSM-5-2D | Thermo-catalysis | H2O2 | 30 | 50 | 26.20 | 115.00 | 89.90 | ^41^ |
| 5 | Ag_1_−Cu_1_/ZSM-5 | Thermo-catalysis | H2O2 | 30 | 70 | 40230.00 | 708.90 | 82.76 | ^42^ |
| 6 | Au-ZSM-5 | Thermo-catalysis | O2 | 24.2 | 240 | 136.25 | -**^c^** | 77.00 | ^43^ |
| 7 | Pd/CsPMA | Thermo-catalysis | O2 | 11 | RT**^b^** | 67.40 | -**^c^** | ~100.00 | ^44^ |
| 8 | Pd-iC-CeO_2_ | Thermo-catalysis | H2O2 | 4 | 75 | 112.00 | -**^c^** | 100.00 | ^45^ |
| 9 | Pd-def-In_2_O_3_ | Photo-catalysis | O2 | 20 | RT**^b^** | ~637.00 | ~1790.00 | 82.50 | ^18^ |
| 10 | Au_NPs_/In_2_O_3_ | Photo-catalysis | O2 | 30 | RT**^b^** | 1983.33 | -**^c^** | 89.42 | ^46^ |
| 11 | Au/TiO_2_ | Photo-catalysis | O2 | 10 | RT**^b^** | 1098.00 | ~100.00 | ~99.00 | ^47^ |
| 12 | PMOF-RuFe(OH) | Photo-catalysis | O2 | AP**^d^** | RT**^b^** | 8810.00 | -**^c^** | 100.00 | ^48^ |
| 13 | AuPd/GaN | Photo-catalysis | CO2 | AP**^d^** | RT**^b^** | 535.00 | 31.00 | 77.11 | ^49^ |
| 14 | AC-Co_1_/PCN_KOH_ | Photo-catalysis | H_2_O | 8 | RT**^b^** | 59.21 | 0.19 | 94.10 | ^50^ |
| 15 | Cl-(H_2_O)_4_Fe^IV^O | Photo-catalysis | O2 | 20 | RT**^b^** | 6760.00 | -**^c^** | 73.60 | ^51^ |

**a**, Selectivity of methyl oxygenates (CH_3_OH and CH_3_OOH). **b**, Room temperature. **c**, Not mentioned or below the detection limit. **d**, Atmospheric pressure.

**Supplementary Table 3 Crystallinity and Strain of Various Catalysts**

| **Entry** | **Sample** | **Crystallinity (%)** | **(222) 2θ (degree)** | **Strain (%)** | **Relatives Strain (%)** |
| --- | --- | --- | --- | --- | --- |
| 1 | In_2_O_3_ | 55.87 | 30.74 | -0.016 | 100.00% |
| 2 **^a^** | InNT | 53.83 | 30.74 | -0.015 | 100.00% |
| 3 | Cu/InNT | 59.67 | 30.74 | -0.047 | 99.97% |
| 4 | Ni/InNT | 62.75 | 30.8 | 0.046 | 100.06% |
| 5 | CuNi@InNT | 57.21 | 30.72 | 0.015 | 100.03% |
| 6 | NiCu/InNT | 56.57 | 30.62 | 0.046 | 100.06% |
| **7** | **CuNi/InNT** | **54.25** | **30.76** | **-0.115** | **99.90%** |

**a**, the data of InNT sample was set as the benchmark to evaluate the influence of different active metals.

Comparing all metal-loaded samples with pure InNT carriers, it was found that all these samples’ relative crystallinity increased, indicating that metal species were likely to be embedded in defect sites or form small nanoparticles over the surface. By analyzing the simulated straining results, it is found that NiCu@InNT and NiCu/InNT presented stretched strain, while CuNi/InNT was compressed. Combined with the reactivity results, it could be assumed that compressed strain is more beneficial to CH_4_ activation.

**Supplementary Table 4 Surface Concentrations of Elements on Various Catalysts**

| **Entry** | **Sample** | **Indium** | | **Oxygen** | | | | | **In/O_La_ Ratio by XPS (%)^d^** | **Copper** | | **Nikel** | | **Cu/Ni^D^** |
| --- | --- | --- | --- | --- | --- | --- | --- | --- | --- | --- | --- | --- | --- | --- |
|  |  |  |  | **Lattice Oxygen** | | | **Oxygen Vacancy^b^** | **Surface -OH^c^** |  |  |  |  |  |  |
|  |  | **Peak (eV)** | **Deviation (eV)** | **Peak (eV)** | **Deviation (eV)** | **Ratio (%)** | **Ratio (%)** | **Ratio (%)** |  | **Peak (eV)** | **Cu/In (%)^d^** | **Peak (eV)** | **Ni/In (%)^d^** |  |
| 1 | In_2_O_3_ | 444.30 | 0.16 | 529.82 | 0.14 | 67.7 | 22.9 | 9.5 | 84.1 | / | **/** | **/** | **/** | **/** |
| **2 ^a^** | **InNT** | **444.14** | **/** | **529.68** | **/** | **69.1** | **22.0** | **8.9** | **82.2** | **/** | **/** | **/** | **/** | **/** |
| 3 | Cu/InNT | 444.24 | 0.10 | 529.78 | 0.10 | 46.6 | 20.9 | 32.5 | 86.6 | 932.37 | 0.8 | / | **/** | **/** |
| 4 | Ni/InNT | 444.41 | 0.27 | 529.87 | 0.19 | 52.8 | 30.9 | 16.3 | 92.9 | **/** | **/** | 853.23 | 2.7 | **/** |
| 5 | NiCu@InNT | 444.28 | 0.14 | 529.81 | 0.13 | 64.4 | 25.4 | 10.2 | 85.3 | 932.54 | 0.8 | 854.52 | 4.7 | 0.16 |
| 6 | NiCu/InNT | 444.33 | 0.19 | 529.84 | 0.16 | 61.9 | 26.8 | 11.3 | 86.9 | 932.14 | 0.9 | 853.00 | 3.7 | 0.26 |
| **7** | **CuNi/InNT** | **444.27** | **0.13** | **529.81** | **0.13** | **65.1** | **25.2** | **9.7** | **85.5** | **932.52** | **1.2** | **853.00** | **2.7** | **0.44** |

**a,** The data of In_2_O_3_ sample was set as the benchmark for comparison. **b,** Peak assigned to Oxygen Vacancy was at 531.37 eV, except for Cu/InNT and Ni/InNT samples. **c,** Peak assigned to Surface -OH was at 532.45 eV, except for Cu/InNT and Ni/InNT samples. **d,** O_La_ standards for Lattice Oxygen, all ratios were semi-quantification results from XPS fitting curves.

A comparison between InNT and In_2_O_3_ revealed that the In/lattice oxygen ratio in InNT was lower, indicating the presence of indium vacancies. Comparative analysis of Cu and Ni with In suggested that a portion of the metal species were loaded within the tubular structures. The comparison between CuNi/InNT and NiCu/InNT further illustrated the impact of the loading sequence on the generation of active sites.

**Supplementary Table 5 Detailed NO-DRIFTS Peak Position of Indium catalysts**

| **Entry** | **Sample** | **NO_2_ (Adsorbed)** | **Monodentate Nitrite** | **Monodentate Nitrate** | **Bidentate Chelating Nitrate** | **Bidentate Bridging Nitrate** |
| --- | --- | --- | --- | --- | --- | --- |
| 1 | In_2_O_3_ | 1631 |  | 1515 |  |  |
| 2 | InNT | 1612 |  | 1517 |  |  |
| 3 | Cu/InNT |  | 1572 |  | 1436 |  |
| 4 | Ni/InNT |  | 1568 |  |  | 1403 |
| 5 | NiCu@InNT |  | 1583 |  | 1439 |  |
| 6 | NiCu/InNT | 1607 |  |  | 1443 |  |
| **7** | **CuNi/InNT** |  | 1579 |  | 1417 |  |

**Supplementary Table 6 Calculated Mulliken Charge of Different Configurations**

| **Entry** | **Element** | **In** | | | | | | | **O** | | | | | | **Cu** | **Ni** | | | | |
| --- | --- | --- | --- | --- | --- | --- | --- | --- | --- | --- | --- | --- | --- | --- | --- | --- | --- | --- | --- | --- |
|  | **Number** | **1** | **2** | **3** | **4** | **5** | **6** | **7** | **1** | **2** | **3** | **4** | **5** | **6** | **1** | **1** | **2** | **3** | **4** | **5** |
| 1 | In_2_O_3_ | 1.320 | 1.320 | 1.330 | 1.310 | 1.330 | 1.310 | 1.300 | -0.900 | -0.900 | -0.920 | -0.930 | -0.930 | -0.920 | / | / | / | / | / | / |
| 2 | InNT | / | 1.320 | 1.350 | 1.310 | 1.330 | 1.300 | 1.310 | -0.830 | -0.800 | / | -0.930 | -0.920 | -0.930 | / | / | / | / | / | / |
| 3 | Cu/InNT | / | 1.310 | 1.370 | 1.240 | 1.330 | 1.270 | 1.360 | -0.880 | -0.940 | / | -0.880 | -0.890 | -0.900 | 0.270 | / | / | / | / | / |
| 4 | Ni/InNT | / | 1.290 | 1.210 | 1.330 | 1.330 | 1.240 | 1.340 | -0.840 | -0.830 | / | -0.860 | -0.920 | -0.940 | / | 0.850 | 0.740 | 0.820 | 0.830 | 0.490 |
| 5 | NiCu/InNT | / | 1.120 | 1.320 | 1.350 | 1.320 | 1.180 | 1.340 | -0.840 | -0.810 | / | -0.870 | -0.910 | -0.930 | 0.210 | 0.680 | 0.740 | 0.800 | 0.600 | 0.510 |
| **6** | **CuNi/InNT** | **/** | **1.230** | **1.370** | **1.270** | **1.350** | **1.270** | **1.320** | **-0.830** | **-0.810** | **/** | **-0.860** | **-0.870** | **-0.900** | **0.230** | **0.470** | **/** | **/** | **/** | **/** |
| 7 | CuPd/InNT |  | 1.320 | 1.410 | 1.280 | 1.350 | 1.320 | 1.370 | -0.840 | -0.820 |  | -0.860 | -0.860 | -0.900 | 0.290 | Pd=0.150 | | | | |
| 8 | CuZn/InNT |  | 1.330 | 1.400 | 1.280 | 1.380 | 1.290 | 1.360 | -0.890 | -0.900 |  | -0.870 | -0.860 | -0.890 | 0.170 | Zn: 0.630 | | | | |
| 9 | CuCu/InNT |  | 1.280 | 1.360 | 1.270 | 1.360 | 1.280 | 1.300 | -0.870 | -0.860 |  | -0.870 | -0.860 | -0.880 | 0.330 | Cu: 0.340 | | | | |

**Supplementary Table 7 Calculated Formation Energy of Different Configurations**

| **Entry** | **Configurations** | **Origin** | **Formation Energy (eV)** |  | **Entry** | **Configurations** | **Origin** | **Formation Energy (eV)** |
| --- | --- | --- | --- | --- | --- | --- | --- | --- |
| **1** | **In_2_O_3_** | -- | **0.000000000** |  | 8 | Cu/InNT-3 | InNT-3 | -0.444815 |
| 2 | InNT-1 | In_2_O_3_ | 0.717635566 |  | 9 | Cu/InNT-4 | InNT-3 | -0.336815 |
| 3 | InNT-2 | In_2_O_3_ | -0.096364434 |  | 10 | Ni/InNT-1 | InNT-3 | 0.679281 |
| **4** | **InNT-3** | **In_2_O_3_** | **-0.494364434** |  | **11** | **Ni/InNT-2** | **InNT-3** | **-1.2040345** |
| 5 | InNT-4 | In_2_O_3_ | 0.985635566 |  | **12** | **NiCu/InNT** | **Ni/InNT-2** | **2.606245** |
| 6 | Cu/InNT-1 | InNT-3 | 0.41137875 |  | **13** | **CuNi/InNT-1** | **Cu/InNT-2** | **0.4454435** |
| **7** | **Cu/InNT-2** | **InNT-3** | **-1.91941325** |  | 14 | CuNi/InNT-2 | Cu/InNT-2 | 2.024582928 |

**Supplementary Table 8 Detailed In-situ DRIFTS Peak Position of Indium Catalysts**

| **Entry** | **Sample** | ***CH_4_** | ***OH** | ***OH_2_** | **M-OH** | ***CO_2_** | **CO_2_ Related**  **Intermediate^A^** | |
| --- | --- | --- | --- | --- | --- | --- | --- | --- |
| 1 | In_2_O_3_ | 3016/1304 | 3401 | 1633 |  |  |  |  |
| 2 | InNT | 3016/1304 | 3416 |  |  | 2355 | 1581 | 1446 |
| 3 | Cu/InNT | 3016/1304 |  |  | 3658  (Cu-OH) | 2353 | 1565 | 1435 |
| 4 | Ni/InNT | 3016/1304 | 3455 |  | 3647  (Ni-OH) | 2321 | 1563 | 1403 |
| 5 | NiCu@InNT | 3016/1304 | 3441 |  |  | 2333 | 1574 | 1434 |
| 6 | NiCu/InNT | 3016/1304 |  |  |  | 2335 | 1575 |  |
| **7** | **CuNi/InNT** | 3016/1304 | 3455 |  | 3698  (Cu/Ni-OH) | 2324 | 1550 | 1411 |

**Supplementary Table 9 Calculated Atom Distance of Different Configurations**

|  | Element | O | | | In | | |
| --- | --- | --- | --- | --- | --- | --- | --- |
| Entry | Number**^A^** | 1~5 | 2~4 | 3~6 | 2~5 | 3~6 | 4~7 |
| 1 | In_2_O_3_ | 4.463 | 4.194 | 4.476 | 7.270 | 6.656 | 7.160 |
| 2 | InNT | 4.938 | 5.311 | / | 7.515 | 6.592 | 7.266 |
| 3 | Cu/InNT | 4.712 | 4.731 | / | 7.613 | 6.716 | 7.364 |
| 4 | Ni/InNT | 4.548 | 5.215 | / | 7.476 | 6.791 | 7.162 |
| 5 | NiCu/InNT | 4.236 | 5.099 | / | 7.550 | 6.982 | 7.248 |
| 6 | CuNi/InNT | 4.637 | 4.763 | / | 7.602 | 6.682 |  |

**a**, The sequence number of the atoms is the same as the chapter for determining catalyst configuration.

**Supplementary Table 10 Stress Tensor of Different Configurations**

| ***Cartesian Components (GPa)*** | | | | | | | | | | | | | |
| --- | --- | --- | --- | --- | --- | --- | --- | --- | --- | --- | --- | --- | --- |
| **In_2_O_3_** | ***X*** | ***Y*** | ***Z*** |  | **Cu/InNT** | ***X*** | ***Y*** | ***Z*** |  | **NiCu/InNT** | ***X*** | ***Y*** | ***Z*** |
| ***X*** | 2.2267 | -0.0002 | 0.0023 |  | ***X*** | 1.7275 | -0.0419 | -0.0004 |  | ***X*** | 1.5968 | -0.0496 | -0.0031 |
| ***Y*** | -0.0002 | 2.2275 | -0.0164 |  | ***Y*** | -0.0419 | 1.6829 | 0.0018 |  | ***Y*** | -0.0496 | 1.4827 | -0.0019 |
| ***Z*** | 0.0023 | -0.0164 | 0.0879 |  | ***Z*** | -0.0004 | 0.0018 | -0.1098 |  | ***Z*** | -0.0031 | -0.0019 | -0.0970 |
| ***Pressure*** | ***-1.514*** | | |  | ***Pressure*** | ***-1.100*** | | |  | ***Pressure*** | ***-0.994*** | | |
|  |  |  |  |  |  |  |  |  |  |  |  |  |  |
| **InNT** | ***X*** | ***Y*** | ***Z*** |  | **Ni/InNT** | ***X*** | ***Y*** | ***Z*** |  | **CuNi/InNT** | ***X*** | ***Y*** | ***Z*** |
| ***X*** | 1.9221 | -0.0465 | -0.0026 |  | ***X*** | 1.7716 | -0.0321 | -0.0013 |  | ***X*** | 1.7756 | -0.0467 | 0.0103 |
| ***Y*** | -0.0465 | 1.6927 | 0.0008 |  | ***Y*** | -0.0321 | 1.5188 | 0.0046 |  | ***Y*** | -0.0467 | 1.6635 | -0.0013 |
| ***Z*** | -0.0026 | 0.0008 | -0.1083 |  | ***Z*** | -0.0013 | 0.0046 | -0.1057 |  | ***Z*** | 0.0103 | -0.0013 | -0.0862 |
| ***Pressure*** | ***-1.169*** | | |  | ***Pressure*** | ***-1.062*** | | |  | ***Pressure*** | ***-1.118*** | | |

**Supplementary Table 11 Calculated Mulliken Charge of Different Configurations during Reaction**

| **Entry** | **Motif** | **Cu** | **Ni** | **Cu-Ni** | ***OH^a^** | | | ***CH_4_^b^** | | | ***CH_3_OH^b^** | | | |
| --- | --- | --- | --- | --- | --- | --- | --- | --- | --- | --- | --- | --- | --- | --- |
|  | **Element** | **Cu** | **Ni** | **Total** | **O** | **H** | **Total** | **C** | **H** | **Total** | **C** | **H** | **O** | **Total** |
| **1** | **CuNi/InNT** | **0.230** | **0.470** | 0.700 | / | / | / | / | / | / | / | / | / | / |
| 2 | CuNi/InNT ~ 2*OH | 0.550 | 0.760 | 1.310 | -0.740 | 0.370 | -0.370 | / | / | / | / | / | / | / |
| 3 | CuNi/InNT ~ *OH + *CH_4_ | 0.410 | 0.830 | 1.240 | / | / | / | -0.930 | 0.600 | -0.330 | / | / | / | / |
| 4 | CuNi/InNT ~ *CH_3_OH | 0.400 | 0.640 | 1.040 | / | / | / | / | / | / | -0.510 | 1.020 | -0.670 | -0.160 |

**a**, Only the charge of the Cu-Ni bridged *OH was considered, for the other *OH species would not form ·OH radical, instead it would form H_2_O with *H from subsequently adsorbed CH_4_. **b**, the charges of H in those configurations were not equal. This table only showed the total values, and the specific values related to the reaction mechanism have been provided in the main text.

**Supplementary Table 12. Detailed catalytic performance of Continuous H_2_O_2_ Injection Reactions**

| **Entry** | **Sample** | **H_2_O_2_ Concentration (%)** | **Productivity (μmol/g_cat_/h)** | | | | | | **CH_3_OH Selectivity**  **(%)** | **CH_3_-R Selectivity (%)** | **CH_3_OH/CH_3_OOH Ratio** |
| --- | --- | --- | --- | --- | --- | --- | --- | --- | --- | --- | --- |
|  |  |  | **CH_3_OH** | **CH_3_OOH** | **CH_2_(OH)_2_** | **HO(CH_2_O)_n_** | **HCOOH** | **CH_3_-R^a^** |  |  |  |
| 1 | CuNi/InNT | 0.1 | 4093.33 | 803.51 | b.d.**^c^** | b.d.**^c^** | b.d.**^c^** | 4896.84 | 83.59 | 100 | 5.09 |
| 2 | CuNi/InNT | 0.5 | 10558.97 | 5279.49 | 3801.23 | b.d.**^c^** | b.d.**^c^** | 15838.46 | 53.76 | 80.65 | 2.00 |
| 3 | CuNi/InNT | 1 | 18628.43 | 6706.24 | 6892.52 | 1676.56 | b.d.**^c^** | 25334.67 | 54.95 | 74.73 | 2.78 |
| 4 | CuNi/InNT | 2 | 31192.46 | 23082.42 | 15596.23 | b.d.**^c^** | b.d.**^c^** | 54274.88 | 44.64 | 77.68 | 1.35 |
| **5^b^** | **CuNi/InNT** | **1** | **36818.84** | **2577.32** | **6627.39** | **368.19** | **b.d.^c^** | **39396.16** | **79.37** | **84.92** | **14.29** |

**a**, CH_3_-R stands for methyl oxygenates (the addition of CH_3_OH and CH_3_OOH). **b**, This reaction was conducted in a 1000 mL reactor with 15 bar CH_4_, 70 °C, 500 rpm for 0.5 hour (300 mL H_2_O + 6 mL/min diluted H_2_O_2_ for 45 min), while other reactions were conducted in a 100 mL reactor with 30 bar CH_4_, 70 °C, 500 rpm for 0.5 hour (20 mL H_2_O + 1 mL/min diluted H_2_O_2_ for 45 min). **c**, Below detection limit.

**Reference.**

1. Segall, M. D.; Philip, J. D. L.; Probert, M. J.; Pickard, C. J.; Hasnip, P. J.; Clark, S. J.; Payne, M. C., First-principles simulation: ideas, illustrations and the CASTEP code. *Journal of Physics: Condensed Matter* **2002**.

2. Perdew, n.; Burke, n.; Ernzerhof, n., Generalized Gradient Approximation Made Simple. *Physical Review Letters* **1996**.

3. Kim, K. C.; Moschetta, E. G.; Jones, C. W.; Jang, S. S., Molecular Dynamics Simulations of Aldol Condensation Catalyzed by Alkylamine-Functionalized Crystalline Silica Surfaces. *Journal of the American Chemical Society* **2016**.

4. Xia, Y.; Li, Q.; Lv, K.; Tang, D.; Li, M., Superiority of graphene over carbon analogs for enhanced photocatalytic H2-production activity of ZnIn2S4. *Applied Catalysis B: Environment and Energy* **2017**.

5. Zhang, Y.; Zhang, N.; Tang, Z.-R.; Xu, Y.-J., Graphene Transforms Wide Band Gap ZnS to a Visible Light Photocatalyst. The New Role of Graphene as a Macromolecular Photosensitizer. *ACS Nano* **2012**.

6. Pan, Y.-X.; You, Y.; Xin, S.; Li, Y.; Fu, G.; Cui, Z.; Men, Y.-L.; Cao, F.-F.; Yu, S.-H.; Goodenough, J. B., Photocatalytic CO2 Reduction by Carbon-Coated Indium-Oxide Nanobelts. *Journal of the American Chemical Society* **2017**.

7. Yang, Y.; Pan, Y.-X.; Tu, X.; Liu, C.-j., Nitrogen doping of indium oxide for enhanced photocatalytic reduction of CO2 to methanol. *Nano Energy* **2022**.

8. Wang, Q.; Chen, Y.; Liu, X.; Li, L.; Du, L.; Tian, G., Sulfur doped In2O3-CeO2 hollow hexagonal prisms with carbon coating for efficient photocatalytic CO2 reduction. *Chemical Engineering Journal* **2021**.

9. Cao, Y.-D.; Yin, D.; Li, S.; Dong, X.-Y.; Feng, Y.; Liu, H.; Fan, L.-L.; Gao, G.-G.; Zang, S.-Q., Substituent Effect to Fine-Tune Energy Levels of Atom-Precise [MoOS3]2− Modified Copper(I) Thiolate Clusters Boosting Recyclable Photocatalysis. *Angewandte Chemie International Edition* **2023**.

10. Chao, W.; Li, W.; Jun, J.; Jing, L.; Yu, L.; Min, W.; Lihua, C.; Binjie, W.; Xiaoyu, Y.; Bao-Lian, S., Probing effective photocorrosion inhibition and highly improved photocatalytic hydrogen production on monodisperse PANI@CdS core-shell nanospheres. *Applied Catalysis B: Environment and Energy* **2016**.

11. Mao, S.; Shi, J.-W.; Sun, G.; Zhang, Y.; Ji, X.; Lv, Y.; Wang, B.; Xu, Y.; Cheng, Y., Cu (II) decorated thiol-functionalized MOF as an efficient transfer medium of charge carriers promoting photocatalytic hydrogen evolution. *Chemical Engineering Journal* **2020**.

12. Li, M.; Zhao, Z.; Xia, Z.; Luo, M.; Zhang, Q.; Qin, Y.; Tao, L.; Yin, K.; Chao, Y.; Gu, L.; Yang, W.; Yu, Y.; Lu, G.; Guo, S., Exclusive Strain Effect Boosts Overall Water Splitting in PdCu/Ir Core/Shell Nanocrystals. *Angewandte Chemie International Edition* **2021**.

13. Wu, T.; Sun, M.; Huang, B., Probing the Irregular Lattice Strain-Induced Electronic Structure Variations on Late Transition Metals for Boosting the Electrocatalyst Activity. *Small* **2020**.

14. Jie, C.; Zhenzi, L.; Haitao, Y.; Xiuwen, W.; Ying, X.; Wei, Z., Periodic quantum well mediated oriented charge separation in Cd0.3Zn0.7S twin crystal towards optimized photocatalytic hydrogen evolution. *Materials Science and Engineering: R: Reports* **2024**.

15. Albani, D.; Capdevila-Cortada, M.; Vilé, G.; Mitchell, S.; Martin, O.; López, N.; Pérez-Ramírez, J., Semihydrogenation of Acetylene on Indium Oxide: Proposed Single-Ensemble Catalysis. *Angewandte Chemie International Edition* **2017**.

16. Chang, X.; Liu, J.; Guo, Z.; Cheng, Y.; Yan, Q.; Li, Y.-Y., Enhanced photocatalytic performance of Bi2MoO6 via strain engineering through collaborative optimization of indium doping and oxygen vacancies. *Applied Catalysis B: Environment and Energy* **2024**.

17. Ye, F.; Zhang, S.; Cheng, Q.; Long, Y.; Liu, D.; Paul, R.; Fang, Y.; Su, Y.; Qu, L.; Dai, L.; Hu, C., The role of oxygen-vacancy in bifunctional indium oxyhydroxide catalysts for electrochemical coupling of biomass valorization with CO2 conversion. *Nature Communications* **2023**.

18. Luo, L.; Fu, L.; Liu, H.; Xu, Y.; Xing, J.; Chang, C.-R.; Yang, D.-Y.; Tang, J., Synergy of Pd atoms and oxygen vacancies on In2O3 for methane conversion under visible light. *Nature Communications* **2022,** *13*, 2930.

19. Zhihao, F.; Mengyuan, H.; Bing, L.; Feng, J.; Yuebing, X.; Xiaohao, L., Identifying the crucial role of water and chloride for efficient mild oxidation of methane to methanol over a [Cu2(μ-O)]2+-ZSM-5 catalyst. *Journal of Catalysis* **2021**.

20. Zhu, C.; Kleimeier, N. F.; Turner, A. M.; Singh, S. K.; Fortenberry, R. C.; Kaiser, R. I., Synthesis of methanediol [CH2(OH)2]: The simplest geminal diol [Chemistry]. *Proceedings of the National Academy of Sciences of the United States of America* **2022**.

21. Wang, W.; Wang, X.; Ma, Z.; Wang, Y.; Yang, Z.; Zhu, J.; Lv, L.; Ning, H.; Tsubaki, N.; Wu, M., Carburized In2O3 Nanorods Endow CO2 Electroreduction to Formate at 1 A cm–2. *ACS Catalysis* **2022**.

22. Jiehong, H.; Pin, L.; Bo, J.; Shaoshuai, C.; Haoran, D.; Jian, Z.; Hexing, L., A novel amorphous alloy photocatalyst (NiB/In2O3) composite for sunlight-induced CO2 hydrogenation to HCOOH. *Applied Catalysis B: Environment and Energy* **2021**.

23. Naik, A. J. T.; Gruar, R.; Tighe, C. J.; Parkin, I. P.; Darr, J. A.; Binions, R., Environmental sensing semiconducting nanoceramics made using a continuous hydrothermal synthesis pilot plant. *Sensors and Actuators B: Chemical* **2014**.

24. Jacukowicz-Sobala, I.; Ciechanowska, A.; Kociołek-Balawejder, E.; Gibas, A.; Zakrzewski, A., Photocatalytically-assisted oxidative adsorption of As(III) using sustainable multifunctional composite material – Cu2O doped anion exchanger. *Journal of Hazardous Materials* **2022**.

25. Wang, O.; Kong, J.; Xue, Z.; An, B.; Xu, J.; Wang, X., Tailoring the Ni–O Microenvironment in Amorphous-Dominated Highly Active and Stable Zn/NiO for Hydrogen Sulfide Detection. *ACS Sensors* **2024**.

26. Chade, L.; Lixiang, Z.; Hengjie, L.; Zhiwei, F.; Chunshuang, Y.; Mengxin, C.; Yi, K.; Carmen, L.; Daobin, L.; Shuzhou, L.; Jiawei, L.; Li, S.; Gang, C.; Qingyu, Y.; Guihua, Y., Selective electrocatalytic synthesis of urea with nitrate and carbon dioxide. *Nature Sustainability* **2021**.

27. Jiang, X.; Lis, B. M.; Purdy, S. C.; Paladugu, S.; Fung, V.; Quan, W.; Bao, Z.; Yang, W.; He, Y.; Sumpter, B. G.; Page, K.; Wachs, I. E.; Wu, Z., CO2-Assisted Oxidative Dehydrogenation of Propane over VOx/In2O3 Catalysts: Interplay between Redox Property and Acid–Base Interactions. *ACS Catalysis* **2022**.

28. Maleki, B.; Kalanakoppal Venkatesh, Y.; Siamak Ashraf Talesh, S.; Esmaeili, H.; Mohan, S.; Balakrishna, G. R., A novel biomass derived activated carbon mediated AC@ZnO/NiO bifunctional nanocatalyst to produce high-quality biodiesel from dairy industry waste oil: CI engine performance and emission. *Chemical Engineering Journal* **2023**.

29. Derikvandi, H.; Nezamzadeh-Ejhieh, A., Synergistic effect of p-n heterojunction, supporting and zeolite nanoparticles in enhanced photocatalytic activity of NiO and SnO2. *Journal of Colloid and Interface Science* **2016**.

30. Eagleton, A. M.; Ko, M.; Stolz, R. M.; Vereshchuk, N.; Meng, Z.; Mendecki, L.; Levenson, A. M.; Huang, C.; MacVeagh, K. C.; Mahdavi-Shakib, A.; Mahle, J. J.; Peterson, G. W.; Frederick, B. G.; Mirica, K. A., Fabrication of Multifunctional Electronic Textiles Using Oxidative Restructuring of Copper into a Cu-Based Metal–Organic Framework. *Journal of the American Chemical Society* **2022**.

31. Gao, F.; Tang, X.; Yi, H.; Chu, C.; Li, N.; Li, J.; Zhao, S., In-situ DRIFTS for the mechanistic studies of NO oxidation over α-MnO2, β-MnO2 and γ-MnO2 catalysts. *Chemical Engineering Journal* **2017**.

32. Guo, F.; Mao, C.; Liang, C.; Xing, P.; Yu, L.; Shi, Y.; Cao, S.; Wang, F.; Liu, X.; Ai, Z.; Zhang, L., Triangle Cl−Ag1−Cl Sites for Superior Photocatalytic Molecular Oxygen Activation and NO Oxidation of BiOCl. *Angewandte Chemie International Edition* **2023**.

33. Lin, B.; Bao, Z.; Wang, A.; Ding, Y.; Zhan, W.; Wang, L.; Guo, Y.; Dai, Q.; Guo, Y.; Gao, F., An efficient Co-Ni hydrous oxide catalyst for elimination of NO pollutant in semi-enclosed spaces at ambient temperature. *Applied Catalysis B: Environment and Energy* **2023**.

34. Han, P.; Zhang, J.; Zhang, W.; Niu, Z.; Wang, G.; Li, X.; Li, J.; Wang, N.; Wang, X.; Wei, H.; Chen, Y.; Li, X., Promotional role of Ni photodepositing on Ru confined TiO2 nanotubes catalyzed CO2 methanation. *Chemical Engineering Journal* **2024**.

35. Lyu, C.; Li, Y.; Cheng, J.; Yang, Y.; Wu, K.; Wu, J.; Wang, H.; Lau, W.-M.; Tian, Z.; Wang, N.; Zheng, J., Dual Atoms (Fe, F) Co-Doping Inducing Electronic Structure Modulation of NiO Hollow Flower-Spheres for Enhanced Oxygen Evolution/Sulfion Oxidation Reaction Performance. *Small* **2023**.

36. Wang, J.; Huang, L.; Sun, B.; Zhang, H.; Hou, D.; Qiao, X.-q.; Ma, H.; Li, D.-S., Efficient photothermal catalytic CO2 reduction over in situ construction ZnIn2S4@Ni(OH)2/NiO Z-scheme heterojunction. *Chemical Engineering Journal* **2023**.

37. Aurnob, A. K. M. K.; Ding, K.; Kauffman, D. R.; Spivey, J. J., Low temperature catalytic conversion of CH4, CO2, and C2H4 to value-added C3 oxygenates and olefins via C1-C2 coupling on Pd-Au/CeO2. *Applied Catalysis B: Environment and Energy* **2022**.

38. Azancot, L.; Bobadilla, L. F.; Centeno, M. A.; Odriozola, J. A., IR spectroscopic insights into the coking-resistance effect of potassium on nickel-based catalyst during dry reforming of methane. *Applied Catalysis B: Environment and Energy* **2020**.

39. Yu, T.; Li, Z.; Lin, L.; Chu, S.; Su, Y.; Song, W.; Wang, A.; Weckhuysen, B. M.; Luo, W., Highly Selective Oxidation of Methane into Methanol over Cu-Promoted Monomeric Fe/ZSM-5. *ACS Catalysis* **2021**.

40. Tang, X.; Wang, L.; Yang, B.; Fei, C.; Yao, T.; Liu, W.; Lou, Y.; Dai, Q.; Cai, Y.; Cao, X.-M.; Zhan, W.; Guo, Y.; Gong, X.-Q.; Guo, Y., Direct Oxidation of Methane to Oxygenates on Supported Single Cu Atom Catalyst. *Applied Catalysis B: Environment and Energy* **2020**.

41. Li, H.; Shen, Y.; Xiao, X.; Jiang, H.; Gu, Q.; Zhang, Y.; Lin, L.; Luo, W.; Zhou, S.; Zhao, J.; Wang, A.; Zhang, T.; Yang, B., Controlled-Release Mechanism Regulates Rhodium Migration and Size Redistribution Boosting Catalytic Methane Conversion. *ACS Catalysis* **2023**.

42. Yu, B.; Cheng, L.; Dai, S.; Jiang, Y.; Yang, B.; Li, H.; Zhao, Y.; Xu, J.; Zhang, Y.; Pan, C.; Cao, X.-M.; Zhu, Y.; Lou, Y., Silver and Copper Dual Single Atoms Boosting Direct Oxidation of Methane to Methanol via Synergistic Catalysis. *Advanced Science* **2023**.

43. Guodong, Q.; Thomas, E. D.; Ali, N.; Mala, A. S.; Alexander, G. R. H.; Richard, J. L.; Matthew, Q.; Catlow, C. R. A.; David, J. W.; Qian, H.; Donald, B.; Mark, J. H.; Barry, A. M.; Brian, H.; Christopher, J. K.; Xingling, Z.; Feng, D.; Jun, X.; Graham, J. H., Au-ZSM-5 catalyses the selective oxidation of CH4 to CH3OH and CH3COOH using O2. *Nature Catalysis* **2022**.

44. Wang, S.; Fung, V.; Hülsey, M. J.; Liang, X.; Yu, Z.; Chang, J.; Folli, A.; Lewis, R. J.; Hutchings, G. J.; He, Q.; Yan, N., H2-reduced phosphomolybdate promotes room-temperature aerobic oxidation of methane to methanol. *Nature Catalysis* **2023**.

45. Jiménez, J. D.; Lustemberg, P. G.; Danielis, M.; Fernández-Villanueva, E.; Hwang, S.; Waluyo, I.; Hunt, A.; Wierzbicki, D.; Zhang, J.; Qi, L.; Trovarelli, A.; Rodriguez, J. A.; Colussi, S.; Ganduglia-Pirovano, M. V.; Senanayake, S. D., From Methane to Methanol: Pd-iC-CeO2 Catalysts Engineered for High Selectivity via Mechanochemical Synthesis. *Journal of the American Chemical Society* **2024**.

46. Jiang, Y.; Li, S.; Wang, S.; Zhang, Y.; Long, C.; Xie, J.; Fan, X.; Zhao, W.; Xu, P.; Fan, Y.; Cui, C.; Tang, Z., Enabling Specific Photocatalytic Methane Oxidation by Controlling Free Radical Type. *Journal of the American Chemical Society* **2023**.

47. Li, X.; Li, C.; Xu, Y.; Liu, Q.; Bahri, M.; Zhang, L.; Browning, N. D.; Cowan, A. J.; Tang, J., Efficient hole abstraction for highly selective oxidative coupling of methane by Au-sputtered TiO2 photocatalysts. *Nature Energy* **2023**.

48. An, B.; Li, Z.; Wang, Z.; Zeng, X.; Han, X.; Cheng, Y.; Sheveleva, A. M.; Zhang, Z.; Tuna, F.; McInnes, E. J. L.; Frogley, M. D.; Ramirez-Cuesta, A. J.; S Natrajan, L.; Wang, C.; Lin, W.; Yang, S.; Schröder, M., Direct photo-oxidation of methane to methanol over a mono-iron hydroxyl site. *Nature Materials* **2022**.

49. Su, H.; Han, J.-T.; Miao, B.; Salehi, M.; Li, C.-J., Photosynthesis of CH3OH via oxygen-atom-grafting from CO2 to CH4 enabled by AuPd/GaN. *Nature Communications* **2024**.

50. Ding, J.; Teng, Z.; Su, X.; Kato, K.; Liu, Y.; Xiao, T.; Liu, W.; Liu, L.; Zhang, Q.; Ren, X.; Zhang, J.; Chen, Z.; Teruhisa, O.; Yamakata, A.; Yang, H.; Huang, Y.; Liu, B.; Zhai, Y., Asymmetrically coordinated cobalt single atom on carbon nitride for highly selective photocatalytic oxidation of CH4 to CH3OH. *Chem* **2023**.

51. Hailong, T.; Yongqing, M.; Ganhong, Z.; Chuhong, Z.; Meiling, W.; Yilin, L.; Xiao, S., In-situ generation of highly-reactive FeIV=O and its contribution during CH4 conversion to CH3OH. *Applied Catalysis B: Environment and Energy* **2024**.
